# Colorectal cancer risk stratification on histological slides based on survival curves predicted by deep learning

**DOI:** 10.1038/s41698-023-00451-3

**Published:** 2023-09-26

**Authors:** Julia Höhn, Eva Krieghoff-Henning, Christoph Wies, Lennard Kiehl, Martin J. Hetz, Tabea-Clara Bucher, Jitendra Jonnagaddala, Kurt Zatloukal, Heimo Müller, Markus Plass, Emilian Jungwirth, Timo Gaiser, Matthias Steeg, Tim Holland-Letz, Hermann Brenner, Michael Hoffmeister, Titus J. Brinker

**Affiliations:** 1 Digital Biomarkers for Oncology Group, German Cancer Research Center (DKFZ), Heidelberg, Germany; 2 Medical Faculty, University Heidelberg, Heidelberg, Germany; 3 School of Population Health, Faculty of Medicine and Health, UNSW Sydney, Kensington, NSW, Australia; 4 Diagnostic and Research Center for Molecular BioMedicine, Diagnostic & Research Institute of Pathology, Medical University of Graz, Graz, Austria; 5 Institute of Pathology, University Medical Center Mannheim, University of Heidelberg, Mannheim, Germany; 6 Institute of Applied Pathology, Speyer, Germany; 7 Department of Biostatistics, German Cancer Research Center (DKFZ), Heidelberg, Germany; 8 Division of Clinical Epidemiology and Aging Research, German Cancer Research Center (DKFZ), Heidelberg, Germany; 9 Division of Preventive Oncology, German Cancer Research Center (DKFZ) and National Center for Tumor Diseases (NCT), Heidelberg, Germany; 10 German Cancer Consortium (DKTK), German Cancer Research Center (DKFZ), Heidelberg, Germany

**Keywords:** Prognostic markers, Colorectal cancer, Mathematics and computing

## Abstract

Studies have shown that colorectal cancer prognosis can be predicted by deep learning-based analysis of histological tissue sections of the primary tumor. So far, this has been achieved using a binary prediction. Survival curves might contain more detailed information and thus enable a more fine-grained risk prediction. Therefore, we established survival curve-based CRC survival predictors and benchmarked them against standard binary survival predictors, comparing their performance extensively on the clinical high and low risk subsets of one internal and three external cohorts. Survival curve-based risk prediction achieved a very similar risk stratification to binary risk prediction for this task. Exchanging other components of the pipeline, namely input tissue and feature extractor, had largely identical effects on model performance independently of the type of risk prediction. An ensemble of all survival curve-based models exhibited a more robust performance, as did a similar ensemble based on binary risk prediction. Patients could be further stratified within clinical risk groups. However, performance still varied across cohorts, indicating limited generalization of all investigated image analysis pipelines, whereas models using clinical data performed robustly on all cohorts.

## Introduction

Colorectal cancer (CRC) remains among the cancer entities with the highest incidence, especially in developed countries^1^. When CRC is detected early, prognosis is usually good and the cancer can be cured by surgery alone^2,3^. At later stages, prognosis is much worse and (neo-)adjuvant treatment is required to increase cure rates^4^. Most patients with locally advanced CRC and thus clinically high risk (CHR, stages IIb and III) receive chemotherapy, whereas patients with CRC up to stage IIa with clinically low risk (CLR) usually do not. However, this binary staging that is currently implemented in the clinic is not an accurate predictor of prognosis. Some tumors are capable of spreading in early stages and thus require adjuvant therapy. Other tumors have limited spreading ability and patients might be able to forgo adjuvant chemotherapy even at later stages^4,5^. This calls for more specific biomarkers to avoid under- and overtreatment. Deep learning (DL) has already been applied to Hematoxylin-Eosin-(H&E-)stained tissue sections in several studies to identify and/or quantify previously unknown characteristics for CRC risk stratification including survival prediction^6–12^. However, so far, no CRC histology based prognostic algorithm is integrated into clinical practice as yet.

The studies that used DL for risk estimation of CRC patients from whole slide images (WSIs) (see summary in Supplementary Table 1) so far mostly build on a similar approach: the WSIs are tiled and a DL model is used to extract tile features, which are then fed into a “survival network” that provides a prognosis/risk estimation based on the merged tiles’ information. The datasets of the existing studies overlap as well. The differences between the studies mostly lie in variations of elements within this approach. First, studies differ in the input tissue by either restricting the region of interest to tumor tissue^10,11^, tumor and stroma tissue^9^ or a set of different non-tumorous tissue types^8^. Second, the studies used different DL feature extractors mostly based on convolutional neural networks (CNNs), albeit with different pre-trainings. The (pre-)training ranged from approaches that trained the extractor in a supervised manner from scratch on the survival task^10,11^ over approaches that used domain-specifically pre-trained extractors^8,9^ to approaches that applied self-supervised pre-training on large datasets^6,7^. Third, the approaches predict a risk score either by using survival analysis-adjusted loss functions^7,9,12^ or by binary classification, e.g., five-year survival assuming a constant risk and not including censored data^6,11^. Together with a variety of inclusion criteria, evaluation metrics and endpoints, this renders the study landscape in this field quite heterogeneous. At least as importantly, the studies do not investigate extensively how well the proposed approaches generalize to new, independent cohorts. Thus, it is still hard to conclude which approach is the most relevant for an accurate and robust prognosis prediction, which is a prerequisite for a future successful clinical implementation.

We extend the existing studies by predicting a five-year survival curve instead of a single risk score, as a survival curve might contain more information of the individual disease course of a patient than such a single risk score and might therefore be better suited for refining the current CRC risk stratification into CHR and CLR subgroups. Our pipeline was built in a modular way that builds on those constructed in the studies so far, allowing us to exchange each module individually. This enabled us to also investigate how variations regarding input tissue and feature extractor influence the discrimination and calibration of the approach on four cohorts. We also benchmarked our survival curve-based approach against the binary approach that predicts a risk score instead of a survival curve.

## Results

### Patient characteristics

**Fig. 1:**
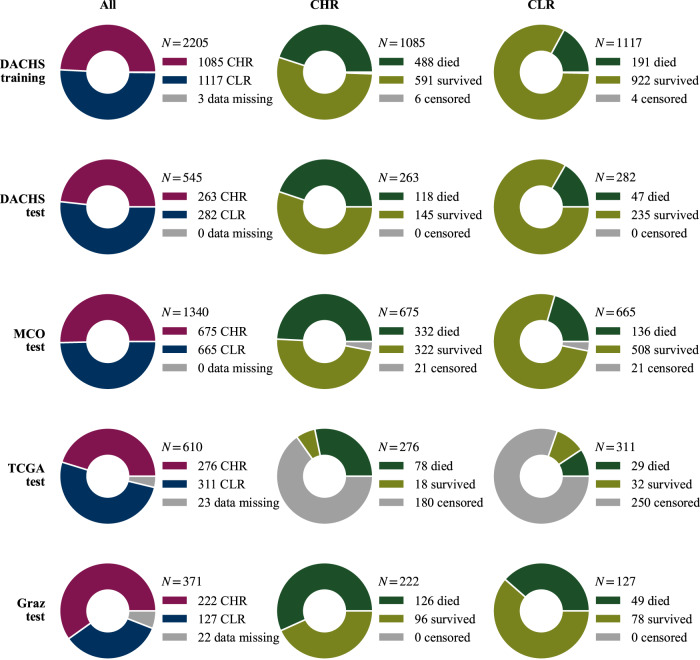
Training and test (sub)cohorts. Entire cohorts (All), clinical high (CHR) and low risk (CLR) subcohorts of all datasets. The term “data missing” refers to information necessary to stratify patients into the CHR or CLR subcohorts. Note that “censored”/”survived” is short for “censored within/survived the first five years after diagnosis”.

Four independent cohorts of stage I-IV CRC cases with clinical follow-up data were used in the study. Our inclusion criteria yielded a training set of 2205 patients from the Darmkrebs: Chancen der Verhütung durch Screening (DACHS) cohort^13–15^ and test sets of 545 patients from DACHS, 1340 patients from the Molecular and Cellular Oncology (MCO) cohort^16^, 610 patients from TCGA^17^ (open evaluations) and 371 patients from Graz^18^ (blinded evaluation). We refer to the Methods section and Supplementary Fig. 1 for cohort details and inclusion criteria. As we wanted to analyze CHR and CLR patients separately, to test directly whether we can further refine the current clinical classification, a few more patients were excluded because of missing clinical parameters (Fig. 1). Clinical characteristics of the patients are shown in Supplementary Table 2.

Follow-up was at least five years and censoring rates during the first five years were low in the DACHS, MCO and Graz cohorts (Table 1), whereas in the TCGA cohort, censoring rates were high and median duration of follow-up was much shorter. Event rates were highest in the TCGA CHR subcohort and in the TCGA and Graz CLR subcohorts compared to the other CHR and CLR subcohorts, respectively.

**Table 1:** Survival data quality in the different datasets.

Dataset	Median overall follow-up time [months]			Censoring rate [%]^a^			Event rate [%]^b^		
	All	CHR	CLR	All	CHR	CLR	All	CHR	CLR
DACHS training	74	62	107	0.5	0.6	0.4	31	45	17
DACHS test	77	62	109	0	0	0	30	45	17
MCO test	60	60	60	3	3	3	36	51	21
TCGA test	21	19	22	73	65	80	70	81	48
Graz test	60	44	64	0	0	0	49	57	39

*CHR* clinical high risk, *CLR* clinical low risk.

^a^Censoring rate describes the percentage of patients that left the study without event during the first five years.

^b^Event rate describes the percentage of cases that experienced the event (=death) within the first five years in relation to all cases that were not censored within the first five years.

### Establishing the modular image analysis pipeline

An image analysis pipeline basically has a modular architecture, where the modules can be varied individually. How the different components influence each other and thereby the performance of the entire pipeline is difficult to predict. As we wanted to compare an optimized pipeline where the risk stratification was based on the prediction of individual patient survival curves with the standard binary approach, we kept this component constant while varying the input tissue types as well as the feature extractor, which were components that the previous studies had also varied. That way, we could investigate how these different variations impact our pipeline’s discrimination and calibration ability across all four cohorts.

**Fig. 2:**
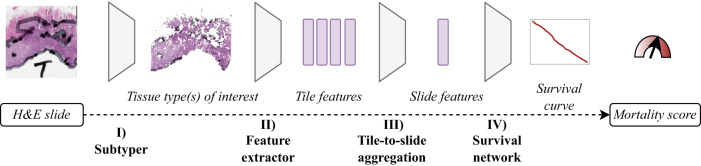
Image analysis pipeline. The pipeline results in an image-based mortality score using H&E slides with deep learning survival curve prediction. In step I, after an H&E slide is segmented into image tiles, a subtyper assigns each tile to one of nine colorectal tissue type classes and only tiles of tissue type(s) of interest are analyzed further. In step II the image tiles are reduced to simplified tile features by a pre-trained feature extractor. In step III all tile features are aggregated to slide features by an attention mechanism. In step IV the slide features are used to predict the patient’s survival curve. The mortality score then aggregates the survival curve in one single value.

Our pipeline contains a subtyper that allows us to select tiles that were classified as (predominantly) a specific tissue type (Fig. 2, step I). Individual tiles are reduced to tile features by a feature extractor (Fig. 2, step II). Through attention-based aggregation, all tile features are combined to slide features (Fig. 2, step III), which are then fed into a survival network to predict a five-year survival curve (Fig. 2, step IV).

We used a piecewise constant hazard function^19^. The survival network predicts the hazard of dying for each month. The pipeline was optimized using the negative log likelihood as loss function. To benchmark our survival-curve based approach against the standard binary approach, our pipeline was also trained for binary classification of five-year overall survival using a binary cross entropy loss weighted with the inverse-probability-of-censoring weighting method^20^.

We investigated different tissue type combinations as input, namely tumor tissue only, stroma tissue only, a combination of stroma and tumor tissue, a combination of non-tumor tissue types (stroma, lymphocytes, mucus) that were previously reported to carry prognostic value, and a combination of all mentioned tissue types (tumor, stroma, lymphocytes, mucus).

The investigated feature extractors included a randomly initialized and untrained ResNet18 model (Rand) to assess the value of any kind of pre-training. As pre-trained feature extractors, we included a standard ResNet18 pre-trained on ImageNet (IM1K), a ResNet18 pre-trained on histologic breast cancer slides to distinguish tumorous from normal tissue (Cam)^21^, the subtyper that we already used to select the tissue types (Sub), a ResNet18 (Ciga)^22^ and a ResNet50 (Retccl)^23^ that were trained on multiple histological datasets in a self-supervised fashion, two small vision transformers that we trained in-house in a self-supervised way on DACHS tiles of different tissue types (DINO-dachs) and on all TCGA slides (DINO-tcga), as well as a hybrid model of a ResNet and a vision transformer pre-trained on ImageNet (R26-ViT).

### Discrimination and calibration of the survival curve versus the binary approach

**Fig. 3:**
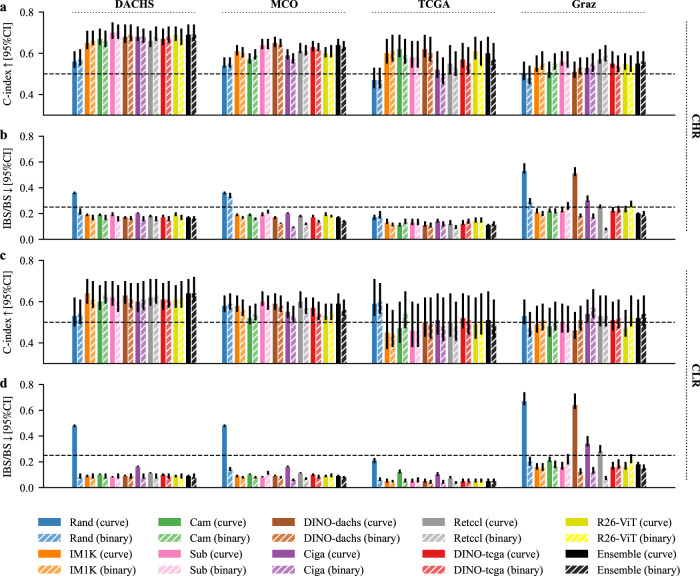
Comparison of the survival curve (curve) and binary approach (binary). **a** C-indices on the four CHR test sets. **b** IBS/BS of the four CHR test sets. **c** C-indices on the four CLR test sets. **d** IBS/BS of the four CLR test sets for all investigated feature extractors and the ensembles. Tumor tissue was used as input tissue in all cases. Note that in case of the single risk score prediction (binary), time independent C-indices and the Brier score were calculated. Arrows indicate whether high or low values are better. The dashed line in each subfigure represents a random performance. In case of the C-index, a value above 0.5 is better than random, in case of the IBS/BS a value below 0.25 is better than random. 95% confidence intervals are shown. BS Brier score, CHR clinical high risk, CI confidence interval, CLR clinical low risk, IBS integrated Brier score.

To compare the discrimination and calibration ability of the survival curve approach against the binary approach thoroughly, we trained 40 models altogether for each approach (5 input tissue combinations, 8 pre-trained feature extractors). We calculated the mean (time-dependent) C-indices and (integrated) Brier scores separately across the four CHR and CLR subcohorts for each model and assessed the distribution differences (Supplementary Fig. 2). Concerning the C-index, we observed no systematic performance difference between both approaches (Supplementary Fig. 2a). Calibration, however, was somewhat worse with the survival curve approach than for the binary approach (Supplementary Fig. 2b). To investigate the impact of the input tissue types, we further split the experiments by input tissue and again illustrated the models’ mean C-indices and (integrated) Brier scores (Supplementary Fig. 3). Models performed better with tumor tissue input alone than with non-tumor tissue(s) and on par with tumor/non-tumor combinations (for numerical values of the performance of all individual models see Supplementary Tables 3 and 4). Figure 3 shows the impact of the investigated feature extractors on the four CHR and CLR subcohorts, separately, using tumor tissue only as input – with the survival curve approach in direct comparison to the binary approach. For both approaches, the models with pre-trained feature extractors performed better than the random model in nearly all cases, suggesting that whatever patterns the pre-trained models identified, these patterns could be used to some extent on all cohorts while the random patterns were clearly much less useful on the external subcohorts. However, there was an usually small, but notable decrease of the models’ discrimination ability (C-index) across all investigated feature extractors on the (external) MCO subcohorts compared to the (internal) DACHS subcohorts. On the smaller external subcohorts of TCGA and Graz, this decrease was even larger. While this general trend was similar for both approaches, they differed in their discrimination ability on single cohorts. Most of the models performed better within the survival curve approach on the MCO and TCGA, but worse on the Graz subcohorts.

Regardless of the approach and in addition to this general decrease in performance, we observed that there were models that still performed very well on two of the external subcohorts, but very poorly on the third, e.g., DINO-dachs in Graz or Ciga in TCGA. At present, one cannot exclude that this might also occur for the models that so far showed good performances on all of our four cohorts on a fifth cohort. Therefore, we decided to also generate ensembles for both approaches by averaging the predictions of the eight models with different feature extractors, respectively and to evaluate their potential to increase prediction robustness. With the survival curve approach, the ensemble yielded a performance broadly similar to the best individual models on all CHR and CLR subcohorts: C-indices ranged from 0.69 (0.67–0.74) in DACHS to 0.55 (0.53–0.61) in Graz for CHR and from 0.64 (0.59–0.71) in DACHS to 0.51 (0.44–0.65) in TCGA for CLR (Fig. 3, Table 2). Results of the ensemble with the binary approach were similar (Fig. 3, Supplementary Table 4).

**Table 2:** Comparison of clinical, image and combined models based on the survival curve approach.

UICC risk group	Model	DACHS	MCO	TCGA	Graz
CHR	Clinical model (Age groups, TNM stages)	0.75 (0.73–0.79)	0.72 (0.71–0.75)	**0**.**70 (0**.**66**–**0**.**77)**	**0**.**65 (0**.**63**–**0**.**71)**
	Image model (Ensemble)	0.69 (0.67–0.74)	0.64 (0.62–0.67)	0.60 (0.56–0.69)	0.55 (0.53–0.61)
	Combined model (Mortality score, age groups, TNM stages)	**0**.**77 (0**.**75**–**0**.**81)**	**0**.**74 (0**.**73**–**0**.**76)**	**0**.**70 (0**.**66**–**0**.**78)**	**0**.**65 (0**.**62**–**0**.**70)**
CLR	Clinical model (Age groups, T stage)	0.65 (0.60–0.73)	**0**.**68 (0**.**65**–**0**.**72)**	**0**.**68 (0**.**63**–**0**.**79)**	0.64 (0.60–0.73)
	Image model (Ensemble)	0.64 (0.59–0.71)	0.59 (0.56–0.64)	0.51 (0.44–0.65)	0.52 (0.47–0.61)
	Combined model (Mortality score, age groups, T stage)	**0**.**68 (0**.**63**–**0**.**76)**	**0**.**68 (0**.**66**–**0**.**73)**	0.64 (0.58–0.75)	**0**.**68 (0**.**64**–**0**.**76)**

*CHR* clinical high risk, *CLR* clinical low risk.

Mortality score is based on the ensemble model (with recalibration on external test sets). Clinical risk factors were the same as used for multivariable Cox regression analysis in Supplementary Fig. 20. C-indices with 95% confidence intervals on internal and external test sets are shown. Best results are highlighted in bold.

### Survival curve separation within the clinical risk groups

**Fig. 4:**
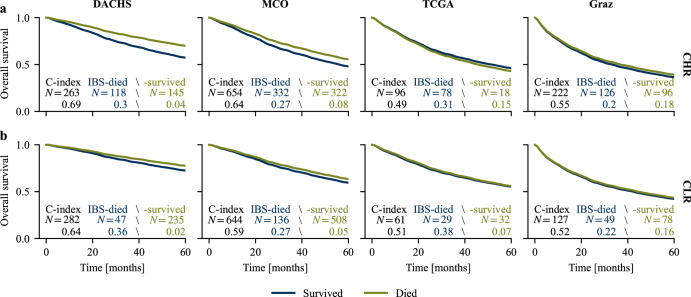
Predicted survival curves of the ensemble of the survival curve approach across subcohorts. Mean predicted survival curves of the ensemble model for patients that died (blue) and survived (green) the first five years for the (**a**), CHR and (**b**), CLR subcohorts. Note that all curves and metrics were calculated without censored cases. Results can therefore differ from the metrics reported in Table 2 and Supplementary Table 3. Mean predicted survival curves of the individual models included in the ensemble are shown in Supplementary Figs. 4–11. CHR clinical high risk, CLR clinical low risk, IBS integrated Brier score.

To investigate the ability of the survival curve approach to further discriminate patient prognoses within the clinically defined CLR and CHR subcohorts, we plotted the mean predicted survival curves of patients that survived or died during the first five years after diagnosis in the CHR and CLR subcohorts. We observed that for the ensemble (Fig. 4a, b) as well as most individual models (Supplementary Figs. 4–11), the curves did indeed separate in all subcohorts. Generally, curve separation was better in CHR than in CLR subcohorts, and in the larger subcohorts DACHS and MCO than in TCGA and Graz. The curves for TCGA CHR were inverted for most models. This may be due to a bias introduced by the (necessary) exclusion of the censored cases (65% of the total) in this analysis, since the C-index generated with the entire subcohort was better than random (see Table 2 and Supplementary Table 3). In general, models performed notably better at predicting the survival curves of surviving patients. For patients that died, the IBS was above 0.25 in most cases, indicating insufficient model calibration. The risk scores of the binary approach showed a similar behavior when analyzed for the patients that survived and died during the first five years (Supplementary Fig. 12). Of note, the risk scores in the TCGA subcohorts were inverted using the binary approach, too.

### Risk group assignment using an image-based mortality score

To assign patients to risk groups within the CHR and CLR subcohorts based on the individual survival predictions, we calculated a mortality score based on the ensemble of the survival curve approach (Fig. 2). We determined separate mortality score thresholds for CHR and CLR on the DACHS training set to distribute the patients into nested, refined risk groups within these subcohorts. Note that we used five-fold cross-validation on the training set for these evaluations. We refer to the Method section and Supplementary Fig. 13 for more details regarding the threshold determination.

For the ensemble (Fig. 4a, b) as well as for the individual models (Supplementary Figs. 4–11), there was an overall tendency towards more pessimistic survival curve predictions in the external test sets for both the CHR and the CLR subcohorts. Calibration curves confirmed a moderate need for recalibration in MCO and a strong need for recalibration in TCGA and Graz (Supplementary Fig. 14). We therefore recalibrated the ensemble on all external test sets individually as described in the Methods, yielding substantially improved calibration curves (Supplementary Fig. 15, also see the mean predicted survival curves for the recalibrated ensemble in Supplementary Fig. 16). Recalibration also substantially improved the IBS in the Graz CLR subcohort, where calibration was the worst, but was negligible in the other cases (see Supplementary Table 5).

**Fig. 5:**
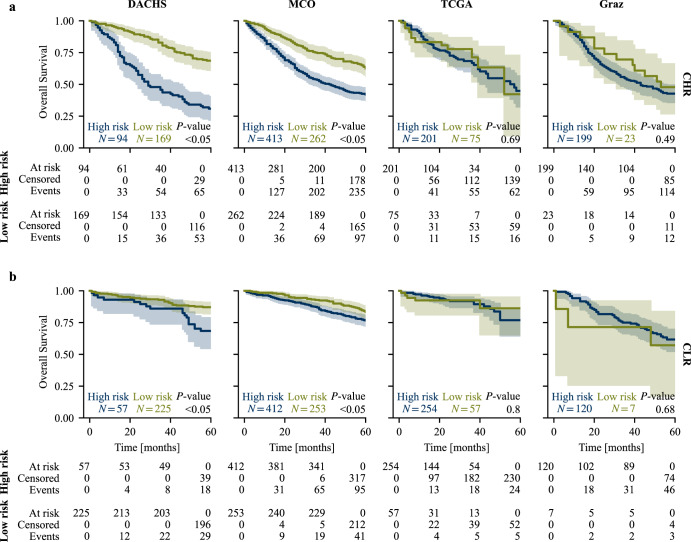
Kaplan-Meier curves of nested risk groups as defined by the survival curve-based ensemble. **a** CHR subcohorts; **b** CLR subcohorts. Recalibration was performed on external test sets. Mortality score cutoffs were determined according to the procedure described in Methods and Supplementary Fig. 13$({M}_{EnsembleCu{t}_{CHR}}=14.9,{M}_{EnsembleCu{t}_{CLR}}=10.5)$. CHR clinical high risk, CLR clinical low risk.

Nested risk stratification (Fig. 5a for CHR, b for CLR subcohorts) was statistically significant within the DACHS and MCO CHR subcohorts (*P* < 0.05 of logrank test, respectively). It was worse in the smaller TCGA and Graz CHR subcohorts, which had higher event rates and for TCGA also a more limited follow-up (Table 1) (*P* = 0.69 and *P* = 0.49 of logrank tests). In the CLR subcohorts, risk stratification was significant for DACHS and MCO (*P* < 0.05 of logrank test, respectively), but not for TCGA (*P* = 0.80) and Graz (*P* = 0.68). For comparison, see the nested risk groups without recalibration in Supplementary Fig. 17.

For the binary approach we proceeded in a similar manner, and with broadly similar results. We determined thresholds directly from the ensemble’s risk score for CHR and CLR subcohorts, respectively (Supplementary Fig. 18). Since the risk scores showed no significant difference between the test sets, no recalibration was done. Kaplan-Meier curves of the nested risk groups are shown in Supplementary Fig. 19. The nested risk stratification of the benchmark ensemble was statistically significant within nearly all CHR subcohorts (DACHS, MCO and TCGA all *P* < 0.05, but Graz *P* = 0.18). In the CLR subcohorts, risk stratification was significant only in MCO (*P* < 0.05), but not in DACHS (*P* = 0.12), TCGA (*P* = 0.16) and Graz (*P* = 0.87).

### Comparison and combination with risk assignment based on clinical data

For the following analyses, we used the ensemble model with the respective recalibration on the external test sets.

In a first step, we performed multivariable Cox regression analysis including the mortality score and known clinical risk factors, namely age and T stage, complemented by N and M stages for the CHR subcohort (see Methods and Supplementary Table 2 for univariate analyses) to check whether the mortality score is a relevant prognostic factor within the clinical risk groups. The mortality score was a significant prognostic factor in the DACHS and MCO subcohorts (DACHS: CHR *P* < 0.05, CLR *P* < 0.05; MCO: CHR *P* < 0.05, CLR *P* < 0.05), but not in the TCGA and Graz subcohorts (TCGA: CHR *P* = 0.06, CLR *P* = 0.4; Graz: CHR *P* = 0.17, CLR *P* = 0.53) (Supplementary Fig. 20).

We then proceeded to compare the performance of the image analysis ensemble with that of a clinical prognostic model based on clinical data known to correlate with prognosis. To do this, we fitted a Cox proportional hazard model (“clinical model”) on CHR and CLR subcohorts of the DACHS training set using the clinical risk factors of the multivariable analyses (Supplementary Fig. 20). In addition, we trained a separate Cox proportional hazard model where we also integrated the mortality score of the image model, to investigate whether a combination of the traditional clinical parameters and the DL-based image analysis could yield a more accurate prognostic biomarker. We refer to the latter as the “combined” model. Details of the fitted models are provided in Supplementary Table 6, ablation studies for other combinations in Supplementary Table 7. With the clinical model alone, we achieved C-indices ranging from 0.75 (0.73–0.79) in DACHS to 0.65 (0.63–0.71) in Graz for the CHR and from 0.68 (0.65–0.72) in MCO to 0.64 (0.60–0.73) in Graz for the CLR subcohorts (see Table 2). If the mortality score was included, the combined model showed numerically, albeit not statistically significant, higher C-indices in four cases, constant C-indices in three and a worse C-index in one case (Table 2).

Results of a similar multivariable Cox regression analysis and combined model using the score of the binary approach instead of the mortality score can be found in Supplementary Fig. 21 and Table 8. The score was a significant prognostic factor only in the DACHS subcohorts (CHR *P* < 0.05, CLR *P* < 0.05) and MCO CHR subcohort (*P* < 0.05) (Supplementary Fig. 21). Also with the combined model based on the binary approach, performance could not be significantly improved compared to the clinical model alone.

## Discussion

Especially for the CHR patients, the predicted survival curves showed a clear difference within patients with good or worse prognosis. With the mortality score that condenses the survival curve prediction in a single value, a statistically significant risk refinement could be achieved in the larger DACHS and MCO subcohorts. However, the approach based on the survival curve prediction did not result in a systematically better risk refinement than the conventional “single risk score” approach. In particular, we found that survival curve-based models show slightly more overall model calibration deficits for individual cohorts, necessitating more effort for recalibration.

A central motivation of predicting survival curves instead of a single risk score is its higher complexity. A single risk score treats the hazard of dying within the first five years as constant. For the survival curve, our model predicts monthly hazards, which was the smallest possible interval within all four cohorts, and thus has the potential to learn a much more time dependent hazard function. However, the exponential decrease of survival curves that could be observed in all models indicates that for this particular use case the models learned a more or less constant hazard within the first five years. In such cases, the greater complexity of the proposed new pipeline is of no direct benefit for the risk refinement. However, our analyses indicate that with appropriate calibration, it is a good method for CRC risk stratification and yields comparable results to the binary approach. For other prognostic tasks where the hazards might be more time-dependent or in case of longer observations or higher event rates, we consider it possible that survival curve prediction may provide an advantage.

Our data show that both the survival curve as well as the binary approach still have a limited ability to generalize, largely independently of the input tissue and feature extractors we employed. Especially regarding the feature extractor, we think that this is a valuable finding. The variety of extractors included a tissue subtyper (Sub), which was similarly used in previous studies^8,9^ as well as models trained on large amounts of histological data in a self-supervised manner (Ciga, Retccl, DINO-tcga). The latter are usually considered to have good generalization capabilities, even though or especially because they are not fine-tuned. In our setting, these extractors did not generalize significantly better than for instance a ResNet18 pre-trained on ImageNet. Furthermore, we also observed that many models performed worse on specific cohorts, even if they worked very well for others. Altogether, performance was still strongly dependent on the individual cohort, with a generally better performance on DACHS and MCO largely independently of the input tissue, feature extractor or survival network we employ, pointing to systematic differences between cohorts. Regarding the Graz cohort, for instance, one obvious difference pertaining to the image analysis is that these CRC slides were scanned with another slide scanner (3D histech) than all the other cohorts (Aperio).

The fact that we performed a very broad investigation of many models on many cohorts is one of the major strengths of this study, since it allowed us to observe differences and outliers in model performance that might have been overlooked otherwise. The ensembles we have included in our analysis, triggered by these observations, show a comparatively robust performance across cohorts and may therefore be a way to compensate for outliers in individual model/cohort constellations. Moreover, combination of such image analysis ensembles with clinical data, which are known to be relatively well-generalizing biomarkers per se, may lead to even better and more robust biomarkers. As only some combined models showed numerically, but not statistically significantly higher C-indices, the relevant prognostic information content of the clinical data and the histological image features may overlap. Therefore, the potential benefit of combining these data entities in a single biomarker remains to be investigated in more detail. Other options to achieve a robust performance on new datasets could be to increase the diversity of the training data, or to perform re-training of the model with a limited amount of data from the new cohort.

As mentioned before, CRC survival prediction based on histological slides has been tried previously (see Supplementary Table 1). Considering that medical images are hard to collect, the studies are quite large, ranging from 1000 to 6000 histological images. This is possible because several large CRC cohorts exist that contain histological images as well as patients and sometimes molecular data. In fact, in our current study, we also made use of some of these cohorts. Although approaches employed in the CRC survival studies conducted using DL-based image analysis with or without clinical data so far are heterogenous, one can approximate that the results we achieved with our survival curve-based image model are broadly similar to those obtained in these earlier studies, more or less independently from the diverse types and pre-trainings of the feature extractors we used (see Supplementary Table 1). This may be due to the fact that survival is not only based on tumor biology, but also on age, comorbidities, chance events etc., i.e., factors that have a very limited impact or no impact at all on tumor tissue morphology.

Due to computational power limitations, our slide-level image analysis pipeline cannot be learned end-to-end. Only the last part of the pipeline could be trained on fixed tile-level features, precluding an extensive usage of common augmentations and bearing a somewhat higher risk for overfitting. Solutions to that problem could be smart feature-level augmentations. Furthermore, we did not perform a comparison with weakly supervised tile-level pipelines or approaches that restrict the number of tiles per sample down to a number that allows end-to-end training, and thus a training of the feature extractor directly for the survival task. This remains to be done in future studies.

In general, patient stratification was better in the larger cohorts and/or cohorts with a higher number of survival events. As expected, the number of cases in the CLR subcohorts was lower than in the CHR cohorts across all investigated study cohorts. Additionally, two of our external test sets were rather small, and at least for the TCGA cohort, follow-up information was very limited. Thus, the results we obtained in the CLR subcohorts and in the TCGA and Graz cohorts in general may have to be considered somewhat preliminary and should be confirmed in larger studies. Moreover, we could not identify a universal threshold that is ideal for all cohorts tested so far, particularly without recalibration, and the predicted survival is not yet accurate in absolute terms. Finally, for recalibration of the survival curve-based models, due to the small size of our external test sets, we did not split the sets into a calibration and test set, but used the whole external test set to estimate the new baseline hazard. Thus, the recalibration effect we showed is a best case scenario and should be re-investigated using separate calibration sets derived from larger test cohorts.

Our results suggest that using DL-based image analysis on histopathological slides and prediction of patient survival curves can further stratify CRC patient prognosis within the UICC-based risk groups that are currently used to a similar extent as when employing a standard binary risk classification. However, as opposed to a clinical classifier, none of the investigated DL image analysis models or ensembles performed equally well on all cohorts. Further attempts must be made to improve model generalization.

## Methods

### Ethics

Data and digitized slides were provided in accordance with the approval of the ethics committees of the Medical Faculty of the University of Heidelberg and the Medical Chambers of Baden-Württemberg and Rhineland-Palatinate for DACHS and in accordance with the approval of the Secure Research Environment for Digital Health (SREDH) Consortium for MCO. TCGA is open source. For Graz, Institutional Review Board approval for this retrospective study using de-identified slides was obtained from the Medical University of Graz (Protocol now. 30–184 ex 17/18). Written informed consent was obtained from each participant in all study cohorts used in this work. The study adheres to the transparent reporting of multivariable prediction models for individual prognosis or diagnosis (TRIPOD) statement^24^. The checklist is shown in Supplementary Table 9.

### Study cohorts and patient flow

The DACHS patient cohort was recruited within a population-based case-control study from southwestern Germany between 2003 and 2014. The MCO cohort is a collection of imaging, specimen, clinical and genetic data from Australian individuals who underwent curative resection for CRC from 1994 to 2010. The Cancer Genome Atlas (TCGA) cohort (TCGA-READ and -COAD) is an international multicentre cohort mainly from the United States with cancers diagnosed between 1998 and 2013. The Graz cohort consists of pathology slides from the Institute of Pathology and the BioBank at the Medical University in Graz of CRC resection cases between 1985 and 2016, while 80% of the resections were diagnosed after 1997.

Cases were eligible if at least one representative pre-treatment diagnostic slide and information on an event or censoring time was available. No (other) image quality checks to exclude cases were undertaken (see Supplementary Fig. 1).

### Predicting survival curves with DL image analysis

We developed DL-based image analysis models to predict the patient’s five-year survival curve directly from diagnostic histological whole slide image(s) (WSIs) without manual annotation or expert analysis (Fig. 2). The chapters “Tiling“ to “Recalibration of the survival curve based image models“ describe the pipeline in more detail.

### Tiling

The slides of the DACHS, MCO and TCGA cohort were scanned with Leica Aperio scanners. The slides of the Graz cohort were scanned with a 3D Histech P1000 scanner. The resolution of the scanned slides differed in between as well as within the cohorts (DACHS ~ 0.5 μm/pixel, MCO ~ 0.25 μm/pixel, TCGA varying between 0.23–0.25 μm/pixel, Graz ~0.12 μm/pixel). To ensure consistent information on tiles from different slides, we decided in favor of a fixed area of 113 μm x 113 μm. This resulted in tiles with different numbers of pixels. The tiles were resized to the required number of pixels (e.g., 224 × 224 pixels) before being provided to the DL feature extractors. The script for the tiling is available under https://github.com/DBO-DKFZ/wsi_preprocessing.

### Subtyper

No manual annotations were performed and the whole slide was tiled. To select tissue of interest, we implemented a colorectal tissue type classifier (“subtyper”, Fig. 2, Step I) adapted from Kather et al.^8^ that classifies all tiles into nine colorectal tissue types (adipose tissue, background, colorectal adenocarcinoma epithelium, debris, lymphocytes, mucus, smooth muscle, normal colon mucosa and stroma). For the subtyper’s backbone we used a ResNet18 initialized with weights provided by Nvidia Clara^21^. They had pre-trained the model on histological images from the CAMELYON16 challenge. We went on to train the backbone and classifier on the non-normalized NCT-CRC-HE-100K set for the nine-tissue-detection task^25^. The NCT-CRC-HE-100K set consists of 100,000 histological image tiles of human colorectal cancer and healthy tissue extracted from 86 H&E stained slides from FFPE samples from the NCT (National Center for Tumor Diseases) Biobank and the UMM (University Medical Center Mannheim) pathology archive and was split by us in a training (66,666 tiles), a validation (16,667 tiles) and an internal test set (16,667 tiles) with similar class distributions. During training we randomly applied the following augmentations with a probability of *p*: colorjitter (brightness = 0.25, contrast = 0.75, saturation = 0.25, hue = 0.5; *p* = 0.9), random horizontal and vertical flips (both with *p* = 0.5), random gray scaling (*p* = 0.1) and gaussian blurring (kernel_size = (5,5), sigma = (0.1,5); *p* = 0.3). The subtyper was further fine-tuned on 8,592 tiles derived from 27 slides of the DACHS cohort that were not used for the survival task (see inclusion criteria in Supplementary Fig. 1). Similar augmentations as above were used for fine-tuning including a random resize crop to compensate for changes in resolution. To ensure a sufficient quality of the subtyper, its performance was evaluated on an internal test set, the “official” validation set CRC-VAL-HE-7K^25^, as well as separate test sets for the DACHS, MCO and subsets of the TCGA cohort. To account for the domain shift of the different submitting sites included in the TCGA cohort, we evaluated the subtyper’s performance on the TCGA set not on all submitting sites together but chose representative submitting sites and analyzed the performance on these sets separately. We used the following submitting sites for evaluation: Harvard (TCGA-H), Greater Poland Cancer Center (TCGA-GPCC) and Christiana Healthcare (TCGA-CH) due to their differences in visual appearance (e.g., different staining). The additional DACHS set for fine-tuning the subtyper as well as the additional test sets for DACHS, MCO and TCGA were created by us using manual annotation (see Supplementary Table 10). Using this subtyper, we investigated different tissue inputs reported as useful in earlier studies (tumor, stroma, mucus and lymphocytes)^8–11,26–28^.

### Feature extractors

Tiles that predominantly contain tissue type(s) of interest were analyzed further by a neural network that generates simplified representations or “features” of all tiles (Fig. 2, Step II). Different feature extractors were investigated, including conventional convolutional neural networks and modern vision transformers (ViT) with supervised and self-supervised pre-training. Models were pre-trained on task-agnostic (natural images, different organ tissue) or domain-specific datasets (colorectal tissue). Details of the investigated extractors are provided in the following. Models trained in-house are marked by^*^. Some models used TCGA slides (partly including CRC cases) for pre-training. In these cases our TCGA cohort can not purely be counted as an external test set. These models are marked by^†^.

#### Rand

A random initialized ResNet18 that served as a baseline since it was not pre-trained for a specific task or with a specific dataset to assess the benefit of any kind of training.

#### IM1K

A ResNet18 pre-trained in a supervised manner (1000 classes) on ImageNet-1K.

#### Cam

A ResNet18 pre-trained in a supervised manner (2 classes) on histological images from the CAMELYON16 challenge by Nvidia Clara to detect breast cancer^21^.

#### Sub^*^

The subtyper developed in this work. It is a ResNet18, pre-trained in a supervised manner to classify nine colorectal tissue types using histological images from different domains. It was first trained on the NCT-CRC-HE-100k cohort^25^ and further fine-tuned on 8592 tiles derived from 27 slides of the DACHS cohort that were not used for the survival task (see inclusion criteria in Supplementary Fig. 1).

#### DINO-dachs*

A ViT-small network trained with the DINO (self-distillation with no labels)^29^ on 0.1 M image tiles of the NCT-CRC-HE-100k cohort^25^ and ~1.2 M images of the DACHS training set containing roughly the same amount of the eight different tissue types (no background class), mimicking the size of ImageNet-1K.

#### Ciga^†^

A ResNet18 pre-trained on 57 histopathological datasets (including multi-organ, different types of staining and resolution properties) with contrastive self-supervised learning method SimCLR^22^.

#### Retccl^†^

A ResNet50 pre-trained on two large histological datasets (TCGA and PAIP) with clustering-guided contrastive learning^23^.

#### DINO-tcga^*†^

A ViT-small network pre-trained with DINO on 10,454 diagnostic, histological slides of TCGA.

#### R26-ViT

A hybrid of a ResNet and ViT-small network as described by Dosovitskiy et al.^30^ pre-trained in a supervised manner on ImageNet-1K.

### Tile-to-slide aggregation

It is unknown if and possibly which tile-level features on a slide carry information that enables a risk stratification regarding overall survival. As a potentially important characteristic might be present on a single, a few or all tiles of a slide, we applied the attention mechanism introduced by Ilse et al.^31^ (Fig. 2, Step III). It combines all *K* tile-level features of a patient $F=\{{f}_{1},\ldots ,{f}_{K}\}$ to slide-level features

1 $$z=\mathop{\sum }\limits_{k=1}^{K}{a}_{k}{f}_{k}$$

with

2 $${a}_{k}=\frac{\exp \{{w}^{T}\,\tanh (V{f}_{k}^{T})\}}{{\sum }_{j=1}^{K}\exp \{{w}^{T}\,\tanh (V{f}_{j}^{T})\}}$$

where $w\in {{\mathbb{R}}}^{Lx1}$ and $V\in {{\mathbb{R}}}^{LxM}$ are trainable parameters.

### Survival network – survival curve

The slide-level features were fed into a small fully connected network (“survival network”) (Fig. 2, Step IV) to predict the patient’s five-year survival curve. The survival network parametrizes the hazards of a Cox proportional hazard model. We applied the approach suggested by Kvamme et al. that assumes piecewise constant continuous-time hazards^19^. Since the patient’s hazard of dying may vary over time, we treated the hazard as constant for the smallest possible time interval available within all four cohorts (one month) and set the survival network’s output nodes to 60.

### Survival network – binary prediction

The slide-level features were fed into a small fully connected network with the same layer constellation as when predicting the survival curve. Instead of 60 output neurons, this survival network has only 1 output neuron.

### Training scheme of image models

We decided in advance that our image models should see all available tiles and be trained on slide-level, since we wanted to avoid any biases due to a selection of tiles or a potential label noise that might come along with weakly supervised training. Histological slides can, however, give rise to thousands of tile images, which cannot be computationally processed in our pipeline at once. To enable a slide-level approach despite computational limitations, the feature extractor was used in a frozen fashion. The tile-to-slide aggregation and the survival network were trained specifically on fixed features. No tile or feature normalizations were used. Overfitting was a problem we had to address. During training, we randomly altered the amount of tiles per sample. We drew features of at least 1000 up to the maximum amount of tiles per sample. If the sample had less than 1000 tiles, we took all tiles. During validation and testing, the features of all tiles per sample were used. We further used dropout with *p* = 0.5 between every layer of the survival network and a weight decay of wd = 1 × 10^−6^. The batch size was always 1. The learning rate warmed up for the first 20 epochs to a learning rate of lr = 1 × 10^−5^ and annealed according to a cosine schedule afterwards. We scheduled a total of 100 epochs, but by applying early stopping, we only took the model with the lowest loss on the validation set, respectively.

For the survival curve approach, we trained the image models with a loss function suggested by Kvamme et al.^19^ implemented in the pycox package. The loss function was defined as the mean negative log likelihood over a batch of *n* patients as follows

3 $$loss=-\frac{1}{n}\mathop{\sum }\limits_{i=1}^{n}({d}_{i}\,\log ({\eta }_{k({t}_{i})})-{\eta }_{k({t}_{i})}\rho ({t}_{i})-\mathop{\sum }\limits_{j=1}^{k({t}_{i})-1}{\eta }_{j})$$

where *d*_*i*_ is the event indicator and *t*_*i*_ is the time of event or censoring of patient *i*, *k*(*t*_*i*_) the time interval *t*_*i*_ falls into (e.g., first month, second month), ${\eta }_{k({t}_{i})}$ is the network’s predicted constant hazard for the time interval *k*(*t*_*i*_) and *ρ*(*t*_*i*_) is the fraction of the last time interval before an event or censoring. Of note, if follow-up was longer than five years, data were administratively censored after five years in this approach.

For the binary approach, we used the binary cross entropy loss. Patients that died within the first five years were labeled “1”, while patients with a follow-up longer than five years and without a death-event were labeled “0”. 11 patients in the training cohort had a follow-up of less than five years. These patients/slides had to be excluded during training. However, to account for these censored cases, we weighted the loss with the inverse-probability-of-censoring-weighting method^20^.

### Validation of image models

For image model development and hyperparameter tuning of both approaches, the DACHS training cohort (*N* = 2205) was split randomly into five-folds for cross-validation (N_fold1-5_ = 441). For each fold, we selected the model with the lowest loss. For the final prediction, we averaged the predictions of the five models of the cross-validation.

### Recalibration of the survival curve based image models

With the survival network, we parameterized the hazards of a Cox proportional hazard model. For recalibration we therefore relied on recalibration methods recommended for Cox regression models^32^. To recalibrate a trained model, we adjusted the predicted hazard function $\hat{h}(t|X)={\hat{h}}_{0}(t){e}^{{\hat{\beta }}^{T}X}$ so that the predicted hazard-rates fit to the observed hazard-rates within our external test sets. We estimated the baseline-hazard function ${\hat{h}}_{0,new}(t)$ based on our external test sets, respectively. To adjust the model predictions, we afterwards adjusted the predicted hazard-rates and obtained a new hazard function ${\hat{h}}_{recalibrated}(t|X)$ with ${\hat{h}}_{recalibrated}(t|X)={\hat{h}}_{0,new}(t)\cdot {e}^{{\hat{\beta }}^{T}X}$. To estimate the baseline hazard of our image models, we fitted a Cox proportional hazard model (CoxPHFitter) included in the lifelines package^33^ on the DACHS training set. Similarly, we estimated the new baseline hazards of all external test sets, respectively. We used those baseline hazard functions to estimate ${\hat{h}}_{recalibrated}(t|X)$.

### Clinical risk groups and DL-based refinement of patient stratification

To analyze the CHR and CLR subcohorts separately, patients were subdivided according to clinical stage: patients with a UICC stage of I to IIa were included in CLR, patients with a UICC stage of IIb to IV in CHR. Based on the predicted survival curve of a patient, we calculated the mortality score

4 $$M=\mathop{\sum }\limits_{t=1}^{T}(H(k(t)|z))$$

with *H* being the cumulative hazards for each time interval *k*(*t*) of the period of observation *T* and *z* being the aggregated slide features^34^. The mortality score condenses the survival curve in one single value. For the binary approach, we used the output score of the image model directly, which can be seen as the risk of dying within the first five years. By defining suitable thresholds for the mortality and risk scores on the DACHS validation sets, patients were assigned to “nested” risk groups within CHR and CLR.

We determined the optimal thresholds within the five-fold cross-validation (Supplementary Fig. 13 for the survival curve approach and Supplementary Fig. 18 for the binary approach). For each validation set of the five-fold cross-validation, we tested all possible mortality/risk scores as thresholds except for the ten lowest and highest scores to ensure a sufficiently large sample size for the logrank test. For each split, we calculated the Kaplan-Meier curves and documented the *P*-value of the logrank test (Step 1). We interpolated the mortality scores for values ranging from minimum to maximum scores with a step size of 0.1 for the overlapping mortality scores among the five validation sets (Step 2). Since the risk score only ranges between 0 and 1, which is lower than the value range of the mortality score, we selected a smaller step size of 0.01. The interpolated curves were averaged (Step 3) and the resulting averaged curve was further smoothed by a gaussian filter with a standard deviation of 1 (Step 4). As the final mortality threshold, we took the mortality score that achieved the lowest *P*-value on the smoothed curve. This procedure was performed for the CHR and CLR cases within the validation sets separately (see Supplementary Figs. 13a, 18a for CHR and 13b/18b for CLR). For the CLR cases, none of the possible mortality score/risk score thresholds achieved a split within the significance level of *P* < 0.05 independently of the approach.

### Multivariable Cox regression analysis, clinical model, combined model

We investigated the benefit of the DL-based image analysis compared to and in addition to already existing risk factors and how the prognostic accuracy and robustness of our risk stratification tool(s) could be further maximized. To do so, we additionally trained a Cox proportional hazard model fitted on clinical data alone and a second, similar model that also encompassed the image-based mortality score.

For these analyses, we used the Cox proportional hazard model (CoxPHFitter) included in the lifelines package^33^. We added a penalizer term with β = 0.1 (l1_ratio = 0.0) to improve stability of the estimates and control for high correlation between covariates. We only included clinical characteristics that were statistically significant in univariate analysis for overall survival in general (Supplementary Table 2), namely age and T stage for the CLR subcohort, complemented by N and M stages for the CHR subcohort. Similarly to Supplementary Table 2, all clinical characteristics were handled as categorical variables. Age was therefore grouped into intervals (<60, 60–69, 79-79, >80). Patients were only considered if there were no missing values in any of the variables (complete case analysis). For the Graz cohort, we imputed missing values for the M stage (provided as MX) as M0, since the MX stage usually results from low risk cases where a full staging is not deemed necessary by the treating physicians. For the combined models, the mortality score or risk score of the ensemble models of the respective approach were added as continuous variables. In case of the survival curve approach, the mortality scores after recalibration on the test sets were used directly and were not normalized.

For multivariable Cox regression analyses, we fitted the models separately on the CHR and CLR subcohort test sets of DACHS (N_CHR_ = 262, N_CLR_ = 282), MCO (N_CHR_ = 675, N_CLR_ = 665), TCGA (N_CHR_ = 245, N_CLR_ = 311) and Graz (N_CHR_ = 200, N_CLR_ = 125).

For the clinical and combined model, the Cox proportional hazard model was fitted on the DACHS CHR and CLR training samples (N_CHR_ = 1075, N_CLR_ = 1117). We predicted survival curves (predict_survival_function) with the clinical and combined models and evaluated their performance with the time-dependent C-index on all test sets to enable consistent comparison with the predictions of the image models of the survival curve approach in Table 2/Supplementary Table 3.

### Evaluation and statistics

We used all available cases to enable optimal training and model building. All models were trained on the DACHS cohort. A hold-out test set was prepared for internal validation through random sampling. The MCO, TCGA and Graz cohorts served as external test sets. Investigation was open in the DACHS, MCO and TCGA cohorts and blinded in the Graz cohort. Blinding was achieved by running the pipeline designed in the DKFZ lab without further changes on the slides and clinical data of the Graz cohort by the partners in Graz.

For the survival curve approach, we used the time dependent C-index^35^ to describe the discrimination of the predicted survival curves and the integrated Brier score (IBS) to evaluate their calibration. We additionally analyzed calibration curves that graphically compare observed and predicted event probabilities to check the models’ validity on different cohorts. As models cannot be used reliably on the new cohort that they are poorly calibrated for, we implemented recalibration to counteract this problem. Evaluation metrics for the binary approach were the C-index^36^ and Brier score^37^.

All metrics are reported with 95% confidence intervals (95% CI), computed with 1000 bootstrap replicates of the model’s predictions on the test samples. To evaluate the quality of our risk stratification, Kaplan-Meier curves for the refined risk groups were calculated and compared using logrank testing. When *P-*values were <0.05, the differences between the groups were considered statistically significant.

### Software

All code was written in Python (3.10.6). The image analysis pipeline used PyTorch (1.13.0+cu117), Pytorch Lightning (1.7.7), NumPy (1.23.3), Pandas (1.5.0) and Scipy (1.9.1). The negative log likelihood loss and functions for the piecewise constant hazard method and to transform the models’ output into a survival curve as well as the time-dependent C-index were taken from pycox (0.2.3). The integrated Brier score and (conventional) Brier score were calculated using sksurv (0.18.0). For the C-index calculation of the binary approach, the Kaplan Meier fitter, Cox proportional hazard fitter and the logrank tests we used the respective functions of lifelines (0.27.2). For visualizations we used matplotlib (3.7.2). Image augmentations (training of the subtyper) were done with torchvision (0.14.0+cu117). Accuracy of the subtyper was calculated with torchmetrics (0.9.3). Pre-trained models were taken from timm (0.6.11), if not stated otherwise.

### Reporting summary

Further information on research design is available in the Nature Research Reporting Summary linked to this article.

### Supplementary information

Supplementary data 1

Supplementary Info

Reporting summary

## Data Availability

This study used archived pseudonymized pathology slides, clinicopathologic variables and corresponding outcome data from the DACHS, MCO, TCGA and Graz cohorts. The DACHS, MCO and Graz cohorts cannot be made publicly available due to general data protection regulations and institutional guidelines. Interested researchers should contact M.H. in case of the DACHS patient cohort and K.Z. in case of the Graz cohort. For access to the MCO cohort visit 10.4225/53/5559205bea135. The TCGA cohort used in this study consists of the TCGA COAD and READ cohorts (both dbGaP accession: phs000178.v11.p8) and original data of both cohorts are publicly available under https://portal.gdc.cancer.gov/ and http://www.cbioportal.org/. Included case IDs of the TCGA cohort are provided in Supplementary Data 1.

## Code availability

The tiling pipeline is available at: https://github.com/DBO-DKFZ/wsi_preprocessing.

Code for the image analysis pipeline is available at: https://github.com/DBO-DKFZ/CRC_DL_Survival_Curves.
