# Supplementary material for: Colorectal cancer risk stratification on histological slides based on survival curves predicted by deep learning
Source: NPJ Precis Oncol. 2023 Sep 26;7:98. doi: 10.1038/s41698-023-00451-3 (PMC10522577; doi:10.1038/s41698-023-00451-3)
Supplement: Supplementary file 2 — Supplementary Info [file 41698_2023_451_MOESM2_ESM.pdf]

Supplementary Material

Supplementary Tables:

Supplementary Table 1: Literature overview of CRC survival prediction by DL-mediated image analysis of H&E slides
Supplementary Table 2: Descriptive characteristics of and univariate Cox regression on all cohorts to calculate overall survival
Supplementary Table 3: Impact of feature extractor and input tissue type on the survival curve approach
Supplementary Table 4: Impact of feature extractor and input tissue type on the binary approach
Supplementary Table 5: Discrimination and calibration of the ensemble model of the survival curve approach with and without recalibration
Supplementary Table 6: Coefficients, P-values and hazard ratios with 95% confidence intervals of all input variables of the clinical and combined models including the mortality score
Supplementary Table 7: Ablation study for the clinical and combined model including the mortality score
Supplementary Table 8: Comparison of clinical, image and combined models of the survival curve approach complemented by the image and combined model of the binary approach
Supplementary Table 9: TRIPOD checklist
Supplementary Table 10: Performance of the subtyper

Supplementary Figures:

Supplementary Figure 1: Flowchart of patient inclusion
Supplementary Figure 2: Impact of risk prediction
Supplementary Figure 3: Impact of input tissue type
Supplementary Figure 4: Mean predicted survival curves of the IM1K model (without recalibration)
Supplementary Figure 5: Mean predicted survival curves of the Cam model (without recalibration)
Supplementary Figure 6: Mean predicted survival curves of the Sub model (without recalibration)
Supplementary Figure 7: Mean predicted survival curves of the DINO-dachs model (without recalibration)
Supplementary Figure 8: Mean predicted survival curves of the Ciga model (without recalibration)
Supplementary Figure 9: Mean predicted survival curves of the Retccl model (without recalibration)
Supplementary Figure 10: Mean predicted survival curves of the DINO-tcga model (without recalibration)
Supplementary Figure 11: Mean predicted survival curves of the R26-ViT model (without recalibration)
Supplementary Figure 12: Risk scores of the binary approach for the patients that died or survived the first five years
Supplementary Figure 13: Cutoff determination for the clinical risk groups on five-fold cross-validation sets of ensemble of the survival curve approach
Supplementary Figure 14: Calibration curves of the all investigated image models of the survival curve approach on the test sets
Supplementary Figure 15: Effect of recalibration on the ensemble of the survival curve approach
Supplementary Figure 16: Mean predicted survival curves of the ensemble of the survival curve approach (with recalibration)
Supplementary Figure 17: Kaplan-Meier curves of “nested” refined risk groups as defined by the ensemble of the survival curve approach within clinical risk groups without recalibration on test sets
Supplementary Figure 18: Cutoff determination for the clinical risk groups on five-fold cross-validation sets of ensemble of the binary approach
Supplementary Figure 19: Kaplan-Meier curves of “nested” refined risk groups as defined by the ensemble of the binary approach within clinical risk groups on test sets

Supplementary Figure 20: Multivariable Cox regression analyses including mortality score and known risk factors for clinical risk groups

Supplementary Figure 21: Multivariable Cox regression analyses including the score of the binary approach and known risk factors for clinical risk groups

Supplementary Tables

Supplementary Table 1: Literature overview of CRC survival prediction by DL-mediated image analysis of H&E slides. For direct comparison, we added the performance of our ensemble of the survival curve approach on our entire four test sets.

Study	Reference in main text	Number of samples	Datasets	DL backbone	Target	Results internal/external
Kather et al.	(8)	2,211	TCGA, DACHS	VGG19 (CNN) pre-trained on CRC tissue types	OS	Hazard ratios
Yao et al.	(12)	1,146	MCO	ImageNet pre-trained VGG (CNN) + multiple instance fully convolutional networks	OS	C-index 0.652/-
Skrede et al.	(10)	4,515	Akerhus University Hospital Norway, Aker University Hospital Norway, Gloucester UK, VICTOR trial, QUASAR 2	Ensemble of ten MobileNetV2 (CNN), supervised training from scratch	CSS	C-index -/0.674[1]
Wulczyn et al.	(11)	5,629	Medical University of Graz	MobileNet-based architecture (CNN)	DSS	C-index 0.663/0.660[2]
Li et al.	(9)	1,117	MCO, TCGA	Xception model (CNN) pre-trained on CRC tissue types	OS	C-index 0.599/0.621[3]
Azizi et al.	(6)	4,496	Medical University of Graz	ResNet architecture (CNN) with large-scale natural image pre-training + contrastive self-supervised learning	DSS	AUC 0.748/0.798[2]
Chen et al.	(7)	588	TCGA	Vision Transformer with hierarchical self-supervised pre-training	OS	C-index 0.608/-
This study		5,071	DACHS, MCO, TCGA, Medical University of Graz	Survival curve-based ensemble image model (with recalibration)	OS	C-index 0.71/0.67/ 0.61/0.56

[1] Skrede et al. used a subset of the Gloucester UK cohort as a test set (internal) and the QUASAR 2 cohort as a validation set (external).
[2] The two test sets are from the same cohort and contain cases based on their respective years to analyze temporal generalization.
[3] Li et al. fitted Cox regression models with the score of the DL model for MCO and TCGA separately and these C-indices were reported (see Supplementary Table 1 of their study).

AUC=Area under the receiver operating curve; CSS=cancer specific survival; CRC=colorectal cancer; CNN=convolutional neural network; DL=deep learning; DSS=disease specific survival; H&E= Hematoxylin and eosin; OS=overall survival.

Supplementary Table 2: Descriptive characteristics of and univariate Cox regression on all cohorts to calculate overall survival. Numbers in bold highlight statistical significance. Hazard ratios (HR) are reported with 95% confidence intervals.

		DACHS training (N=2205)			DACHS test (N=545)			MCO test (N=1340)			TCGA test (N=610)			Graz test (N=371)		
**Characteristic**		**N**	**N%**	**HR**	**N**	**N%**	**HR**	**N**	**N%**	**HR**	**N**	**N%**	**HR**	**N**	**N%**	**HR**
Age	<60	402	18.2	1.0 (reference)	100	18.3	1.0 (reference)	308	23.0	1.0 (reference)	172	28.2	1.0 (reference)	112	30,2	1.0 (reference)
	60-69	651	29.5	**1.3 (1.0-1.68)**	166	30.5	1.44 (0.83-2.51)	372	27.8	1.14 (0.86-1.51)	174	28.5	0.99 (0.55-1.78)	142	38.3	1.05 (0.72-1.54)
	70-79	769	34.9	**1.47 (1.15-1.89)**	192	35.2	**1.82 (1.08-3.09)**	399	29.8	**1.4 (1.07-1.83)**	166	27.2	**2.05 (1.23-3.42)**	87	23.5	**1.88 (1.26-2.8)**
	80+	383	17.4	**2.97 (2.3-3.84)**	87	16.0	**3.95 (2.3-6.81)**	261	19.5	**2.02 (1.53-2.66)**	98	16.1	**2.46 (1.39-4.34)**	30	8.1	**2.68 (1.61-4.47)**
Sex	Female	907	41.1	1.0 (reference)	250	45.9	1.0 (reference)	612	45.7	1.0 (reference)	290	47.5	1.0 (reference)	145	39.1	1.0 (reference)
	Male	1298	58.9	0.97 (0.83-1.13)	295	54.1	0.84 (0.62-1.14)	728	54.3	**1.37 (1.14-1.65)**	320	52.5	1.0 (0.69-1.44)	226	60.9	1.02 (0.76-1.38)
CRC location	Colon	1537	69.7	1..0 (reference)	387	71.0	1.0 (reference)	867	64.7	1.0 (reference)	445	73.0	1.0 (reference)	174	46.9	1.0 (reference)
	Rectum	668	30.3	0.98 (0.83-1.16)	158	29.0	0.93 (0.66-1.31)	450	33.6	1.04 (0.85-1.26)	161	26.4	0.82 (0.53-1.28)	197	53.1	0.88 (0.66-1.18)
	Missing	-	-	-	-	-	-	23	1.7	-	4	0.6	-	-	-	-
Sided-ness	Proximal	1296	58.8	1.0 (reference)	329	60.4	1.0 (reference)	781	58.3	1.0 (reference)	298	48.9	1.0 (reference)	46	12.4	1.0 (reference)
	Distal	879	39.9	1.06 (0.9-1.23)	206	37.8	1.21 (0.88-1.65)	508	37.9	0.89 (0.73-1.08)	212	34.8	1.36 (0.92-2.02)	325	87.6	1.07 (0.68-1.71)
	Missing	30	1.3	-	10	1.8	-	51	4.0	-	100	16.4	-	-	-	-
T stage	1	154	7.0	**0.36 (0.23-0.56)**	30	5.5	0.32 (0.1-1.01)	110	8.2	**0.53 (0.33-0.83)**	19	3.1	0.57 (0.14-2.33)	15	4	1.48 (0.75-2.94)
	2	399	18.1	**0.4 (0.31-0.53)**	92	16.9	**0.48 (0.27-0.83)**	221	16.5	**0.53 (0.38-0.74)**	106	17.4	**0.46 (0.22-0.96)**	39	10.5	**0.4 (0.19-0.82)**
	3	1364	61.9	1.0 (reference)	336	61.7	1.0 (reference)	696	51.9	1.0 (reference)	416	68.2	1.0 (reference)	223	60.1	1.0 (reference)
	4	284	12.9	**1.97 (1.64-2.37)**	86	15.8	**2.7 (1.92-3.8)**	313	23.4	**2.59 (2.13-3.15)**	67	11.0	**3.16 (2.02-4.95)**	66	17.8	**2.44 (1.75-3.42)**
	Missing	4	0.2	-	1	0.1	-	-	-	-	2	0.3	-	28	7.6	-
N stage	N0	1227	55.6	1.0 (reference)	311	57.1	1.0 (reference)	769	57.4	1.0 (reference)	347	56.9	1.0 (reference)	132	35.6	1.0 (reference)
	N1	539	24.4	**1.91 (1.58-2.31)**	126	23.1	**1.91 (1.29-2.84)**	334	24.9	**2.03 (1.63-2.54)**	145	23.8	**1.91 (1.18-3.10)**	107	28.8	1.27 (0.86-1.88)
	N2	412	18.7	**4.03 (3.38-4.81)**	102	18.7	**4.61 (3.23-6.58)**	237	17.7	**3.82 (3.07-4.75)**	115	18.9	**4.43 (2.89-6.80)**	80	21.6	**2.27 (1.55-3.33)**

	Missing	27	1.2	-	6	1.1	-	-	-	-	352	0.514.0	-	52	14.0	-
M stage	M0	1899	86.1	1.0 (reference)	462	84.8	1.0 (reference)	1170	87.3	1.0 (reference)	456	74.8	1.0 (reference)	62	16.7	1.0 (reference)
	M+	303	13.7	**6.66 (5.68-7.81)**	83	15.2	**5.65 (4.11-7.76)**	170	12.7	**6.52 (5.33-7.98)**	86	14.1	**4.08 (2.71-6.15)**	32	8.6	**2.6 (1.5-4.49)**
	Missing	3	0.1	-	-	-	-	-	-	-	68	11.1	-	277	74.7	-
MSI	Stable	1292	58.6	1.0 (reference)	324	59.4	1.0 (reference)	1136	84.8	1.0 (reference)	428	70.2	1.0 (reference)			
	Unstable	161	7.3	**0.66 (0.47-0.92)**	45	8.3	0.82 (0.45-1.49)	197	14.7	**0.7 (0.53-0.93)**	71	11.6	0.66 (0.34-1.28)			
	Missing	752	34.1	-	176	32.3	-	7	0.5	-	111	18.2	-			
BRAF	Wildtype	1393	63.2	1.0 (reference)	338	62.0	1.0 (reference)	1163	86.8	1.0 (reference)	299	49.0	1.0 (reference)			
	Mutated	110	5.0	1.31 (0.95-1.82)	36	6.6	**2.12 (1.28-3.50)**	168	12.5	0.9 (0.67-1.19)	37	6.1	1.06 (0.46-2.47)			
	Missing	702	31.8	-	171	31.4	-	9	0.7	-	274	44.9	-			
KRAS	Wildtype	983	44.6	1.0 (reference)	258	47.3	1.0 (reference)	901	67.2	1.0 (reference)	217	35.6	1.0 (reference)			
	Mutated	495	22.4	1.05 (0.87-1.27)	121	22.2	0.84 (0.57-1.25)	432	32.2	**1.3 (1.07-1.56)**	119	19.5	0.95 (0.56-1.62)			
	Missing	727	33.0	-	166	30.5	-	7	0.5	-	274	44.9	-			

Supplementary Table 3: Impact of feature extractor and input tissue type on the survival curve approach (without recalibration on test sets). For all metrics, the 95% confidence intervals are reported next to the expected values. Arrows indicate whether high or low values are better.

UICC risk group	Feature extractor	Input tissue type	DACHS		MCO		TCGA		Graz	
			C-index ↑	IBS ↓	C-index ↑	IBS ↓	C-index ↑	IBS ↓	C-index ↑	IBS ↓
**CHR**	**Rand**	**TUM**	0.56 (0.53-0.61)	0.36 (0.35-0.37)	0.54 (0.53-0.58)	0.36 (0.35-0.37)	0.47 (0.43-0.53)	0.17 (0.15-0.19)	0.5 (0.47-0.56)	0.53 (0.51-0.59)
		**STR**	0.59 (0.56-0.64)	0.18 (0.17-0.21)	0.49 (0.48-0.53)	0.53 (0.52-0.56)	0.46 (0.42-0.54)	0.15 (0.14-0.18)	0.51 (0.48-0.56)	0.4 (0.38-0.44)
		**TUM+STR**	0.59 (0.56-0.64)	0.18 (0.17-0.21)	0.52 (0.5-0.55)	0.44 (0.43-0.47)	0.46 (0.42-0.53)	0.21 (0.2-0.24)	0.51 (0.48-0.56)	0.41 (0.39-0.45)
		**Other**	0.52 (0.49-0.58)	0.21 (0.2-0.23)	0.47 (0.45-0.5)	0.54 (0.53-0.57)	0.52 (0.48-0.6)	0.13 (0.12-0.15)	0.53 (0.51-0.59)	0.37 (0.35-0.41)
		**ALL**	0.59 (0.56-0.64)	0.18 (0.17-0.21)	0.49 (0.48-0.53)	0.43 (0.42-0.45)	0.42 (0.39-0.48)	0.22 (0.2-0.24)	0.52 (0.5-0.58)	0.39 (0.37-0.43)
	**IM1K**	**TUM**	0.65 (0.62-0.71)	0.19 (0.18-0.2)	0.61 (0.59-0.64)	0.19 (0.18-0.2)	0.6 (0.55-0.67)	0.13 (0.12-0.16)	0.53 (0.51-0.59)	0.21 (0.2-0.24)
		**STR**	0.63 (0.6-0.67)	0.18 (0.16-0.2)	0.6 (0.58-0.63)	0.2 (0.19-0.21)	0.54 (0.5-0.62)	0.11 (0.1-0.14)	0.53 (0.51-0.58)	0.23 (0.22-0.26)
		**TUM+STR**	0.69 (0.66-0.73)	0.16 (0.15-0.18)	0.6 (0.58-0.63)	0.19 (0.18-0.21)	0.55 (0.5-0.63)	0.14 (0.13-0.18)	0.55 (0.52-0.6)	0.22 (0.21-0.25)
		**Other**	0.61 (0.58-0.67)	0.18 (0.16-0.2)	0.59 (0.57-0.62)	0.2 (0.19-0.22)	0.55 (0.52-0.63)	0.12 (0.11-0.14)	0.52 (0.5-0.57)	0.24 (0.23-0.28)
		**ALL**	0.68 (0.65-0.72)	0.16 (0.15-0.18)	0.6 (0.58-0.63)	0.19 (0.18-0.21)	0.55 (0.5-0.63)	0.14 (0.13-0.18)	0.53 (0.5-0.58)	0.23 (0.22-0.26)
	**Cam**	**TUM**	0.67 (0.64-0.72)	0.19 (0.18-0.2)	0.57 (0.55-0.6)	0.19 (0.18-0.2)	0.62 (0.58-0.69)	0.11 (0.1-0.13)	0.51 (0.48-0.56)	0.22 (0.21-0.24)
		**STR**	0.65 (0.62-0.7)	0.17 (0.16-0.2)	0.55 (0.53-0.58)	0.2 (0.19-0.22)	0.58 (0.54-0.66)	0.11 (0.1-0.13)	0.56 (0.53-0.61)	0.2 (0.2-0.23)
		**TUM+STR**	0.66 (0.63-0.71)	0.16 (0.15-0.19)	0.57 (0.55-0.6)	0.19 (0.18-0.2)	0.58 (0.55-0.65)	0.12 (0.11-0.14)	0.51 (0.48-0.57)	0.23 (0.22-0.25)
		**Other**	0.64 (0.61-0.69)	0.17 (0.16-0.2)	0.53 (0.51-0.56)	0.21 (0.2-0.22)	0.59 (0.55-0.66)	0.1 (0.09-0.12)	0.53 (0.5-0.58)	0.21 (0.2-0.24)
		**ALL**	0.66 (0.63-0.71)	0.16 (0.15-0.19)	0.57 (0.55-0.6)	0.19 (0.18-0.2)	0.58 (0.54-0.65)	0.12 (0.11-0.14)	0.53 (0.5-0.58)	0.22 (0.21-0.24)
	**Sub**	**TUM**	0.7 (0.67-0.75)	0.19 (0.18-0.21)	0.64 (0.62-0.67)	0.19 (0.18-0.21)	0.58 (0.53-0.66)	0.13 (0.11-0.16)	0.56 (0.54-0.61)	0.22 (0.21-0.25)
		**STR**	0.67 (0.65-0.72)	0.15 (0.14-0.18)	0.62 (0.61-0.65)	0.21 (0.2-0.22)	0.58 (0.54-0.65)	0.12 (0.1-0.14)	0.59 (0.56-0.63)	0.23 (0.22-0.27)
		**TUM+STR**	0.71 (0.68-0.75)	0.15 (0.14-0.17)	0.64 (0.62-0.67)	0.2 (0.19-0.21)	0.62 (0.58-0.69)	0.12 (0.11-0.15)	0.57 (0.55-0.63)	0.23 (0.22-0.26)
		**Other**	0.46 (0.43-0.52)	0.6 (0.58-0.65)	0.49 (0.47-0.52)	0.52 (0.5-0.55)	0.43 (0.4-0.51)	0.28 (0.27-0.31)	0.47 (0.44-0.53)	0.53 (0.51-0.58)
		**ALL**	0.71 (0.68-0.75)	0.15 (0.14-0.17)	0.64 (0.62-0.67)	0.2 (0.19-0.21)	0.62 (0.58-0.69)	0.12 (0.11-0.15)	0.58 (0.55-0.63)	0.23 (0.22-0.26)
	**Ciga**	**TUM**	0.68 (0.66-0.73)	0.2 (0.2-0.21)	0.59 (0.57-0.62)	0.2 (0.2-0.21)	0.52 (0.48-0.61)	0.14 (0.13-0.16)	0.53 (0.5-0.58)	0.3 (0.29-0.34)
		**STR**	0.66 (0.63-0.71)	0.17 (0.16-0.19)	0.56 (0.55-0.59)	0.2 (0.19-0.22)	0.52 (0.48-0.6)	0.11 (0.1-0.13)	0.56 (0.53-0.61)	0.22 (0.2-0.24)
		**TUM+STR**	0.68 (0.65-0.73)	0.16 (0.15-0.18)	0.57 (0.55-0.6)	0.19 (0.18-0.2)	0.5 (0.46-0.59)	0.12 (0.11-0.14)	0.53 (0.5-0.58)	0.24 (0.23-0.27)

		**Other**	0.66 (0.63-0.71)	0.18 (0.16-0.2)	0.57 (0.55-0.6)	0.21 (0.2-0.23)	0.5 (0.46-0.59)	0.11 (0.1-0.13)	0.55 (0.52-0.6)	0.24 (0.22-0.27)
		**ALL**	0.67 (0.65-0.72)	0.16 (0.15-0.19)	0.56 (0.55-0.6)	0.19 (0.18-0.2)	0.5 (0.46-0.59)	0.12 (0.11-0.14)	0.54 (0.51-0.59)	0.23 (0.22-0.26)
	**Retccl**	**TUM**	0.66 (0.63-0.71)	0.18 (0.17-0.19)	0.61 (0.6-0.65)	0.18 (0.17-0.19)	0.55 (0.5-0.64)	0.12 (0.11-0.15)	0.57 (0.55-0.62)	0.25 (0.24-0.27)
		**STR**	0.66 (0.63-0.71)	0.17 (0.16-0.2)	0.57 (0.55-0.6)	0.19 (0.18-0.21)	0.56 (0.52-0.64)	0.12 (0.1-0.14)	0.55 (0.53-0.61)	0.21 (0.2-0.23)
		**TUM+STR**	0.67 (0.64-0.72)	0.17 (0.15-0.19)	0.61 (0.6-0.65)	0.18 (0.17-0.19)	0.55 (0.51-0.64)	0.12 (0.11-0.14)	0.55 (0.52-0.6)	0.21 (0.2-0.23)
		**Other**	0.62 (0.59-0.67)	0.18 (0.17-0.21)	0.57 (0.55-0.6)	0.2 (0.19-0.22)	0.53 (0.49-0.62)	0.12 (0.11-0.15)	0.52 (0.49-0.58)	0.21 (0.2-0.24)
		**ALL**	0.67 (0.65-0.72)	0.17 (0.15-0.19)	0.62 (0.6-0.64)	0.18 (0.17-0.19)	0.55 (0.5-0.63)	0.12 (0.11-0.15)	0.55 (0.52-0.6)	0.21 (0.2-0.23)
	**DINO-dachs**	**TUM**	0.68 (0.65-0.74)	0.17 (0.16-0.18)	0.65 (0.63-0.68)	0.17 (0.16-0.18)	0.62 (0.58-0.69)	0.11 (0.1-0.14)	0.51 (0.48-0.56)	0.51 (0.49-0.56)
		**STR**	0.64 (0.61-0.69)	0.17 (0.16-0.2)	0.61 (0.59-0.64)	0.18 (0.18-0.19)	0.56 (0.52-0.63)	0.11 (0.1-0.13)	0.5 (0.47-0.55)	0.51 (0.49-0.56)
		**TUM+STR**	0.69 (0.66-0.74)	0.16 (0.15-0.18)	0.65 (0.63-0.68)	0.17 (0.16-0.18)	0.58 (0.54-0.66)	0.11 (0.1-0.13)	0.51 (0.48-0.56)	0.49 (0.47-0.55)
		**Other**	0.64 (0.61-0.69)	0.17 (0.16-0.19)	0.62 (0.6-0.65)	0.18 (0.17-0.19)	0.56 (0.52-0.64)	0.11 (0.1-0.13)	0.48 (0.45-0.53)	0.49 (0.46-0.54)
		**ALL**	0.68 (0.66-0.73)	0.16 (0.15-0.18)	0.64 (0.63-0.67)	0.17 (0.17-0.18)	0.58 (0.54-0.66)	0.11 (0.1-0.13)	0.5 (0.48-0.56)	0.49 (0.47-0.54)
	**DINO-tcga**	**TUM**	0.67 (0.64-0.72)	0.17 (0.17-0.19)	0.63 (0.61-0.66)	0.17 (0.17-0.19)	0.57 (0.53-0.65)	0.12 (0.11-0.15)	0.55 (0.53-0.61)	0.22 (0.21-0.25)
		**STR**	0.66 (0.63-0.71)	0.17 (0.16-0.19)	0.59 (0.58-0.63)	0.19 (0.18-0.2)	0.55 (0.51-0.63)	0.12 (0.1-0.14)	0.56 (0.53-0.61)	0.23 (0.21-0.25)
		**TUM+STR**	0.65 (0.63-0.7)	0.17 (0.15-0.19)	0.61 (0.59-0.64)	0.18 (0.18-0.19)	0.55 (0.51-0.63)	0.13 (0.11-0.15)	0.54 (0.52-0.6)	0.23 (0.21-0.25)
		**Other**	0.64 (0.61-0.69)	0.17 (0.16-0.2)	0.6 (0.58-0.63)	0.19 (0.18-0.2)	0.55 (0.51-0.63)	0.12 (0.11-0.14)	0.53 (0.51-0.59)	0.24 (0.22-0.27)
		**ALL**	0.65 (0.63-0.71)	0.17 (0.15-0.19)	0.6 (0.59-0.64)	0.18 (0.18-0.19)	0.55 (0.51-0.62)	0.13 (0.11-0.15)	0.55 (0.53-0.6)	0.23 (0.22-0.27)
	**R26-ViT**	**TUM**	0.69 (0.66-0.73)	0.19 (0.18-0.21)	0.6 (0.58-0.63)	0.19 (0.18-0.21)	0.61 (0.57-0.68)	0.14 (0.13-0.17)	0.55 (0.52-0.6)	0.23 (0.21-0.26)
		**STR**	0.66 (0.63-0.71)	0.17 (0.16-0.19)	0.6 (0.58-0.63)	0.19 (0.19-0.21)	0.56 (0.52-0.64)	0.11 (0.1-0.14)	0.53 (0.51-0.59)	0.24 (0.23-0.27)
		**TUM+STR**	0.68 (0.65-0.72)	0.16 (0.15-0.18)	0.61 (0.59-0.64)	0.19 (0.18-0.2)	0.58 (0.55-0.66)	0.12 (0.11-0.14)	0.55 (0.52-0.6)	0.22 (0.21-0.25)
		**Other**	0.65 (0.62-0.7)	0.17 (0.16-0.2)	0.59 (0.57-0.62)	0.19 (0.19-0.21)	0.56 (0.52-0.63)	0.11 (0.1-0.13)	0.54 (0.52-0.59)	0.24 (0.22-0.26)
		**ALL**	0.67 (0.65-0.72)	0.16 (0.15-0.18)	0.61 (0.59-0.64)	0.19 (0.18-0.2)	0.58 (0.55-0.66)	0.12 (0.11-0.14)	0.55 (0.52-0.6)	0.23 (0.21-0.26)
	**Ensemble**	**TUM**	0.69 (0.67-0.74)	0.17 (0.17-0.18)	0.64 (0.62-0.67)	0.17 (0.17-0.18)	0.6 (0.56-0.69)	0.11 (0.09-0.12)	0.55 (0.53-0.61)	0.2 (0.19-0.21)
**CLR**	**Rand**	**TUM**	0.53 (0.48-0.62)	0.48 (0.47-0.49)	0.58 (0.55-0.63)	0.48 (0.47-0.49)	0.59 (0.53-0.71)	0.21 (0.19-0.23)	0.53 (0.49-0.61)	0.67 (0.64-0.74)
		**STR**	0.63 (0.59-0.71)	0.08 (0.07-0.1)	0.56 (0.53-0.61)	0.73 (0.72-0.74)	0.44 (0.36-0.59)	0.17 (0.16-0.19)	0.54 (0.5-0.62)	0.52 (0.49-0.6)
		**TUM+STR**	0.58 (0.53-0.66)	0.08 (0.07-0.1)	0.57 (0.54-0.62)	0.61 (0.6-0.63)	0.54 (0.47-0.66)	0.26 (0.25-0.29)	0.49 (0.45-0.58	0.49 (0.45-0.58)
		**Other**	0.45 (0.4-0.53)	0.22 (0.21-0.24)	0.45 (0.42-0.5)	0.74 (0.73-0.76)	0.54 (0.47-0.65)	0.14 (0.14-0.16)	0.53 (0.49-0.61)	0.48 (0.46-0.55)
		**ALL**	0.57 (0.52-0.66)	0.08 (0.07-0.1)	0.53 (0.51-0.59)	0.6 (0.59-0.61)	0.6 (0.53-0.72)	0.27 (0.25-0.3)	0.5 (0.46-0.58)	0.51 (0.49-0.59)

**IM1K**	**TUM**	0.64 (0.59-0.71)	0.09 (0.08-0.1)	0.58 (0.55-0.63)	0.09 (0.08-0.1)	0.45 (0.37-0.59)	0.05 (0.04-0.07)	0.49 (0.45-0.58)	0.16 (0.14-0.19)
	**STR**	0.54 (0.5-0.63)	0.08 (0.07-0.1)	0.53 (0.5-0.58)	0.1 (0.09-0.11)	0.55 (0.49-0.65)	0.05 (0.04-0.06)	0.57 (0.53-0.65)	0.16 (0.14-0.19)
	**TUM+STR**	0.6 (0.56-0.69)	0.09 (0.08-0.1)	0.58 (0.55-0.63)	0.09 (0.08-0.1)	0.49 (0.42-0.63)	0.05 (0.04-0.07)	0.56 (0.52-0.64)	0.16 (0.14-0.19)
	**Other**	0.55 (0.5-0.64)	0.08 (0.07-0.1)	0.54 (0.51-0.59)	0.09 (0.09-0.1)	0.48 (0.42-0.6)	0.05 (0.04-0.07)	0.58 (0.54-0.66)	0.16 (0.14-0.2)
	**ALL**	0.6 (0.56-0.68)	0.09 (0.08-0.1)	0.58 (0.55-0.62)	0.09 (0.08-0.1)	0.46 (0.4-0.59)	0.05 (0.04-0.07)	0.58 (0.54-0.66)	0.16 (0.14-0.19)
**Cam**	**TUM**	0.6 (0.56-0.68)	0.1 (0.1-0.11)	0.52 (0.49-0.58)	0.1 (0.1-0.11)	0.47 (0.4-0.6)	0.12 (0.11-0.14)	0.48 (0.43-0.57)	0.21 (0.2-0.24)
	**STR**	0.56 (0.51-0.64)	0.08 (0.07-0.1)	0.51 (0.48-0.57)	0.09 (0.09-0.1)	0.49 (0.42-0.62)	0.11 (0.1-0.12)	0.55 (0.51-0.63)	0.16 (0.15-0.19)
	**TUM+STR**	0.64 (0.6-0.72)	0.08 (0.07-0.1)	0.49 (0.47-0.55)	0.11 (0.1-0.12)	0.47 (0.41-0.59)	0.12 (0.11-0.14)	0.49 (0.44-0.57)	0.22 (0.21-0.25)
	**Other**	0.55 (0.51-0.64)	0.08 (0.07-0.1)	0.49 (0.46-0.54)	0.09 (0.08-0.1)	0.46 (0.4-0.6)	0.1 (0.09-0.12)	0.53 (0.49-0.61)	0.16 (0.15-0.18)
	**ALL**	0.65 (0.6-0.73)	0.08 (0.07-0.1)	0.49 (0.46-0.54)	0.1 (0.1-0.11)	0.47 (0.41-0.59)	0.12 (0.11-0.13)	0.49 (0.45-0.57)	0.2 (0.18-0.23)
**Sub**	**TUM**	0.62 (0.58-0.7)	0.08 (0.08-0.09)	0.6 (0.58-0.65)	0.08 (0.08-0.09)	0.46 (0.38-0.6)	0.05 (0.04-0.07)	0.5 (0.45-0.59)	0.16 (0.14-0.2)
	**STR**	0.57 (0.53-0.66)	0.08 (0.07-0.1)	0.55 (0.52-0.61)	0.08 (0.08-0.1)	0.44 (0.35-0.59)	0.06 (0.05-0.08)	0.5 (0.45-0.6)	0.17 (0.15-0.21)
	**TUM+STR**	0.61 (0.57-0.7)	0.08 (0.07-0.1)	0.58 (0.55-0.63)	0.08 (0.08-0.1)	0.45 (0.39-0.58)	0.05 (0.04-0.07)	0.52 (0.47-0.61)	0.17 (0.15-0.21)
	**Other**	0.47 (0.43-0.56)	0.76 (0.75-0.79)	0.5 (0.47-0.56)	0.71 (0.7-0.73)	0.45 (0.39-0.57)	0.35 (0.33-0.38)	0.45 (0.41-0.53)	0.67 (0.64-0.75)
	**ALL**	0.61 (0.57-0.69)	0.08 (0.07-0.1)	0.58 (0.55-0.63)	0.08 (0.08-0.1)	0.44 (0.38-0.57)	0.05 (0.04-0.07)	0.52 (0.48-0.61)	0.17 (0.15-0.21)
**Ciga**	**TUM**	0.6 (0.55-0.69)	0.16 (0.16-0.17)	0.55 (0.52-0.6)	0.16 (0.16-0.17)	0.51 (0.44-0.64)	0.1 (0.09-0.12)	0.54 (0.5-0.61)	0.34 (0.32-0.4)
	**STR**	0.63 (0.58-0.71)	0.08 (0.07-0.1)	0.58 (0.55-0.63)	0.09 (0.08-0.1)	0.51 (0.45-0.64)	0.07 (0.06-0.08)	0.57 (0.53-0.64)	0.17 (0.15-0.21)
	**TUM+STR**	0.6 (0.56-0.69)	0.08 (0.07-0.1)	0.53 (0.5-0.58)	0.14 (0.14-0.15)	0.53 (0.46-0.67)	0.08 (0.08-0.1)	0.56 (0.52-0.64)	0.24 (0.22-0.28)
	**Other**	0.65 (0.61-0.73)	0.07 (0.06-0.1)	0.58 (0.55-0.62)	0.09 (0.08-0.1)	0.55 (0.48-0.66)	0.06 (0.06-0.08)	0.57 (0.53-0.65)	0.17 (0.15-0.21)
	**ALL**	0.61 (0.56-0.69)	0.08 (0.07-0.1)	0.53 (0.5-0.57)	0.14 (0.14-0.15)	0.54 (0.46-0.68)	0.08 (0.07-0.1)	0.59 (0.55-0.68)	0.18 (0.16-0.21)
**Retccl**	**TUM**	0.62 (0.58-0.71)	0.11 (0.11-0.12)	0.6 (0.57-0.65)	0.11 (0.11-0.12)	0.5 (0.43-0.64)	0.07 (0.07-0.09)	0.53 (0.48-0.63)	0.28 (0.27-0.33)
	**STR**	0.59 (0.54-0.67)	0.08 (0.07-0.1)	0.57 (0.54-0.62)	0.09 (0.09-0.1)	0.54 (0.48-0.67)	0.07 (0.06-0.09)	0.57 (0.52-0.66)	0.2 (0.18-0.23)
	**TUM+STR**	0.63 (0.58-0.71)	0.08 (0.06-0.1)	0.59 (0.56-0.64)	0.1 (0.1-0.11)	0.48 (0.41-0.61)	0.07 (0.06-0.09)	0.6 (0.57-0.69)	0.19 (0.18-0.21)
	**Other**	0.62 (0.58-0.7)	0.08 (0.07-0.1)	0.56 (0.53-0.61)	0.09 (0.08-0.1)	0.52 (0.45-0.65)	0.07 (0.06-0.09)	0.56 (0.51-0.64)	0.18 (0.16-0.21)
	**ALL**	0.64 (0.59-0.71)	0.08 (0.07-0.1)	0.59 (0.56-0.64)	0.1 (0.1-0.11)	0.46 (0.39-0.59)	0.07 (0.06-0.09)	0.61 (0.57-0.69)	0.17 (0.16-0.2)
**DINO-dachs**	**TUM**	0.63 (0.59-0.7)	0.09 (0.09-0.1)	0.59 (0.56-0.64)	0.09 (0.09-0.1)	0.5 (0.43-0.62)	0.05 (0.04-0.07)	0.46 (0.42-0.55)	0.64 (0.61-0.73)
	**STR**	0.57 (0.53-0.65)	0.08 (0.07-0.1)	0.57 (0.55-0.63)	0.1 (0.09-0.11)	0.46 (0.4-0.58)	0.06 (0.05-0.07)	0.51 (0.47-0.6)	0.65 (0.61-0.73)

	**TUM+STR**	0.6 (0.56-0.68)	0.08 (0.07-0.1)	0.58 (0.56-0.63)	0.1 (0.1-0.11)	0.5 (0.44-0.63)	0.05 (0.04-0.07)	0.45 (0.42-0.53)	0.63 (0.6-0.71)
	**Other**	0.61 (0.56-0.69)	0.08 (0.07-0.1)	0.6 (0.58-0.65)	0.1 (0.09-0.11)	0.48 (0.42-0.59)	0.06 (0.05-0.07)	0.5 (0.45-0.58)	0.62 (0.59-0.71)
	**ALL**	0.62 (0.58-0.69)	0.08 (0.07-0.1)	0.6 (0.57-0.65)	0.11 (0.1-0.12)	0.49 (0.43-0.61)	0.06 (0.05-0.07)	0.44 (0.41-0.52)	0.62 (0.59-0.71)
**DINO-tcga**	**TUM**	0.61 (0.56-0.69)	0.1 (0.09-0.11)	0.57 (0.53-0.62)	0.1 (0.09-0.11)	0.52 (0.47-0.64)	0.05 (0.04-0.07)	0.51 (0.46-0.6)	0.16 (0.14-0.2)
	**STR**	0.57 (0.52-0.66)	0.08 (0.07-0.1)	0.59 (0.56-0.64)	0.09 (0.09-0.1)	0.46 (0.41-0.57)	0.05 (0.04-0.07)	0.53 (0.48-0.62)	0.16 (0.14-0.19)
	**TUM+STR**	0.6 (0.56-0.69)	0.08 (0.07-0.1)	0.56 (0.53-0.62)	0.1 (0.09-0.11)	0.52 (0.45-0.64)	0.05 (0.04-0.07)	0.57 (0.53-0.65)	0.16 (0.14-0.19)
	**Other**	0.62 (0.57-0.7)	0.08 (0.07-0.1)	0.6 (0.58-0.65)	0.09 (0.08-0.1)	0.55 (0.49-0.67)	0.05 (0.04-0.07)	0.58 (0.54-0.66)	0.15 (0.14-0.19)
	**ALL**	0.61 (0.57-0.7)	0.08 (0.07-0.1)	0.56 (0.54-0.62)	0.1 (0.09-0.11)	0.5 (0.44-0.63)	0.05 (0.04-0.07)	0.58 (0.54-0.66)	0.16 (0.14-0.2)
**R26-ViT**	**TUM**	0.61 (0.57-0.68)	0.09 (0.08-0.1)	0.53 (0.51-0.59)	0.09 (0.08-0.1)	0.49 (0.42-0.62)	0.05 (0.04-0.07)	0.47 (0.43-0.56)	0.16 (0.14-0.2)
	**STR**	0.59 (0.54-0.67)	0.08 (0.07-0.1)	0.57 (0.54-0.61)	0.09 (0.08-0.1)	0.55 (0.48-0.67)	0.05 (0.04-0.06)	0.55 (0.5-0.63)	0.16 (0.14-0.2)
	**TUM+STR**	0.64 (0.59-0.72)	0.08 (0.07-0.1)	0.53 (0.5-0.58)	0.09 (0.08-0.1)	0.48 (0.4-0.63)	0.05 (0.04-0.07)	0.48 (0.44-0.57)	0.16 (0.14-0.2)
	**Other**	0.58 (0.55-0.67)	0.08 (0.07-0.1)	0.56 (0.53-0.61)	0.09 (0.09-0.1)	0.5 (0.44-0.61)	0.05 (0.04-0.06)	0.58 (0.54-0.67)	0.16 (0.14-0.19)
	**ALL**	0.63 (0.58-0.7)	0.08 (0.07-0.1)	0.54 (0.51-0.59)	0.09 (0.08-0.1)	0.47 (0.39-0.61)	0.05 (0.04-0.06)	0.52 (0.47-0.6)	0.16 (0.14-0.2)
**Ensemble**	**TUM**	0.64 (0.59-0.71)	0.09 (0.09-0.1)	0.59 (0.56-0.64)	0.09 (0.09-0.1)	0.51 (0.44-0.65)	0.05 (0.05-0.07)	0.52 (0.47-0.61)	0.18 (0.18-0.2)

CHR=clinical high risk; CLR=clinical low risk; IBS=integrated Brier score; TUM=tumor; STR=stroma; TUM+STR=tumor and stroma; other=stroma, lymphocytes and mucus; ALL=tumor, stroma, lymphocytes and mucus.

Supplementary Table 4: Impact of feature extractor and input tissue type on the binary approach. For all metrics, the 95% confidence intervals are reported next to the expected values. Arrows indicate whether high or low values are better.

UICC risk group	Feature extractor	Input tissue type	DACHS		MCO		TCGA		Graz	
			C-index ↑	BS ↓	C-index ↑	BS ↓	C-index ↑	BS ↓	C-index ↑	BS ↓
**CHR**	**Rand**	**TUM**	0.57 (0.54-0.62)	0.21 (0.19-0.24)	0.55 (0.53-0.58)	0.33 (0.32-0.36)	0.44 (0.4-0.52)	0.16 (0.14-0.19)	0.48 (0.45-0.53)	0.29 (0.27-0.32)
		**STR**	0.55 (0.51-0.6)	0.21 (0.19-0.24)	0.51 (0.5-0.55)	0.21 (0.2-0.22)	0.47 (0.44-0.54)	0.15 (0.13-0.18)	0.5 (0.47-0.56)	0.32 (0.3-0.35)
		**TUM+STR**	0.59 (0.56-0.64)	0.21 (0.19-0.23)	0.52 (0.5-0.55)	0.25 (0.24-0.27)	0.53 (0.49-0.61)	0.12 (0.11-0.15)	0.53 (0.49-0.58)	0.34 (0.32-0.38)
		**Other**	0.53 (0.5-0.59)	0.21 (0.2-0.25)	0.51 (0.49-0.54)	0.18 (0.17-0.19)	0.49 (0.45-0.57)	0.15 (0.13-0.18)	0.5 (0.47-0.56)	0.25 (0.23-0.27)
		**ALL**	0.54 (0.51-0.59)	0.21 (0.19-0.24)	0.5 (0.48-0.53)	0.24 (0.22-0.25)	0.44 (0.4-0.52)	0.16 (0.14-0.19)	0.53 (0.5-0.58)	0.29 (0.27-0.33)
	**IM1K**	**TUM**	0.66 (0.64-0.71)	0.16 (0.15-0.19)	0.6 (0.58-0.63)	0.16 (0.16-0.18)	0.61 (0.56-0.68)	0.11 (0.1-0.13)	0.55 (0.52-0.61)	0.19 (0.18-0.22)
		**STR**	0.68 (0.65-0.72)	0.18 (0.17-0.21)	0.6 (0.58-0.63)	0.19 (0.18-0.21)	0.51 (0.46-0.59)	0.12 (0.11-0.15)	0.57 (0.54-0.62)	0.21 (0.2-0.24)
		**TUM+STR**	0.68 (0.65-0.73)	0.16 (0.15-0.19)	0.59 (0.57-0.62)	0.13 (0.13-0.15)	0.57 (0.52-0.65)	0.09 (0.08-0.11)	0.55 (0.52-0.61)	0.15 (0.14-0.17)
		**Other**	0.65 (0.62-0.7)	0.18 (0.16-0.2)	0.6 (0.58-0.63)	0.14 (0.14-0.16)	0.53 (0.48-0.61)	0.12 (0.1-0.14)	0.55 (0.52-0.6)	0.18 (0.17-0.21)
		**ALL**	0.67 (0.64-0.71)	0.17 (0.15-0.19)	0.6 (0.58-0.63)	0.15 (0.14-0.16)	0.58 (0.54-0.67)	0.1 (0.09-0.12)	0.55 (0.51-0.6)	0.18 (0.16-0.2)
	**Cam**	**TUM**	0.66 (0.64-0.71)	0.16 (0.15-0.19)	0.59 (0.57-0.62)	0.16 (0.15-0.17)	0.56 (0.52-0.63)	0.12 (0.1-0.14)	0.55 (0.52-0.61)	0.21 (0.2-0.24)
		**STR**	0.66 (0.63-0.71)	0.18 (0.17-0.21)	0.55 (0.53-0.58)	0.25 (0.24-0.27)	0.58 (0.54-0.66)	0.12 (0.11-0.15)	0.54 (0.51-0.59)	0.24 (0.22-0.27)
		**TUM+STR**	0.65 (0.62-0.7)	0.17 (0.15-0.2)	0.57 (0.55-0.6)	0.15 (0.14-0.16)	0.57 (0.52-0.65)	0.12 (0.1-0.14)	0.54 (0.51-0.59)	0.18 (0.17-0.21)
		**Other**	0.63 (0.6-0.68)	0.19 (0.17-0.22)	0.55 (0.53-0.58)	0.2 (0.19-0.22)	0.58 (0.54-0.66)	0.12 (0.1-0.14)	0.53 (0.5-0.58)	0.22 (0.2-0.25)
		**ALL**	0.65 (0.62-0.69)	0.17 (0.16-0.2)	0.57 (0.55-0.6)	0.14 (0.13-0.15)	0.56 (0.52-0.63)	0.12 (0.1-0.14)	0.54 (0.5-0.59)	0.18 (0.17-0.2)
	**Sub**	**TUM**	0.7 (0.67-0.75)	0.15 (0.14-0.18)	0.64 (0.62-0.67)	0.21 (0.2-0.23)	0.58 (0.53-0.66)	0.13 (0.11-0.16)	0.56 (0.53-0.61)	0.25(0.23-0.29)
		**STR**	0.68 (0.65-0.73)	0.15 (0.14-0.18)	0.62 (0.6-0.65)	0.21 (0.2-0.23)	0.58 (0.55-0.65)	0.09 (0.08-0.11)	0.58 (0.56-0.63)	0.23(0.21-0.26)
		**TUM+STR**	0.7 (0.68-0.75)	0.15 (0.13-0.17)	0.64 (0.62-0.67)	0.23 (0.21-0.25)	0.58 (0.53-0.66)	0.12 (0.11-0.15)	0.59 (0.56-0.64)	0.25 (0.23-0.28)
		**Other**	0.68 (0.65-0.73)	0.15 (0.14-0.18)	0.63 (0.61-0.66)	0.19 (0.18-0.21)	0.56 (0.52-0.64)	0.08 (0.07-0.1)	0.61 (0.58-0.66)	0.23 (0.21-0.26)
		**ALL**	0.7 (0.67-0.75)	0.15 (0.14-0.18)	0.64 (0.62-0.67)	0.22 (0.21-0.24)	0.59 (0.54-0.66)	0.11 (0.1-0.14)	0.59 (0.56-0.64)	0.25 (0.23-0.28)
	**Ciga**	**TUM**	0.68 (0.65-0.72)	0.15 (0.14-0.18)	0.57 (0.55-0.6)	0.09 (0.09-0.1)	0.5 (0.45-0.58)	0.12 (0.1-0.14)	0.55 (0.52-0.6)	0.17 (0.16-0.2)
		**STR**	0.68 (0.65-0.73)	0.18 (0.16-0.2)	0.58 (0.55-0.61)	0.2 (0.19-0.21)	0.5 (0.45-0.58)	0.11 (0.1-0.13)	0.55 (0.52-0.6)	0.27 (0.25-0.3)
		**TUM+STR**	0.66 (0.63-0.71)	0.15 (0.14-0.18)	0.57 (0.55-0.6)	0.09 (0.08-0.1)	0.51 (0.46-0.59)	0.11 (0.09-0.14)	0.55 (0.52-0.61)	0.2 (0.19-0.23)
		**Other**	0.63 (0.6-0.67)	0.18 (0.17-0.21)	0.59 (0.57-0.62)	0.18 (0.17-0.19)	0.5 (0.45-0.57)	0.11 (0.1-0.13)	0.55 (0.52-0.6)	0.25 (0.23-0.27)
		**ALL**	0.65 (0.63-0.7)	0.16 (0.15-0.19)	0.56 (0.54-0.6)	0.09 (0.08-0.1)	0.5 (0.45-0.59)	0.1 (0.08-0.12)	0.56 (0.53-0.62)	0.24 (0.22-0.27)

	**Retccl**	**TUM**	0.68 (0.65-0.72)	0.15 (0.14-0.18)	0.61 (0.59-0.64)	0.12 (0.11-0.13)	0.53 (0.48-0.61)	0.09 (0.08-0.12)	0.59 (0.57-0.64)	0.08 (0.07-0.09)
		**STR**	0.66 (0.63-0.71)	0.18 (0.16-0.21)	0.59 (0.57-0.62)	0.17 (0.16-0.18)	0.52 (0.47-0.61)	0.1 (0.09-0.13)	0.57 (0.54-0.62)	0.15 (0.13-0.17)
		**TUM+STR**	0.69 (0.66-0.74)	0.16 (0.14-0.18)	0.61 (0.6-0.64)	0.12 (0.11-0.13)	0.53 (0.49-0.62)	0.09 (0.08-0.12)	0.56 (0.53-0.61)	0.12 (0.11-0.13)
		**Other**	0.64 (0.61-0.69)	0.17 (0.15-0.2)	0.59 (0.57-0.62)	0.15 (0.14-0.17)	0.55 (0.5-0.62)	0.1 (0.09-0.13)	0.55 (0.52-0.6)	0.14 (0.13-0.17)
		**ALL**	0.69 (0.66-0.74)	0.16 (0.14-0.18)	0.6 (0.58-0.63)	0.12 (0.11-0.13)	0.54 (0.49-0.62)	0.09 (0.08-0.11)	0.57 (0.54-0.62)	0.12 (0.11-0.13)
	**DINO-dachs**	**TUM**	0.69 (0.66-0.74)	0.16 (0.14-0.18)	0.64 (0.62-0.67)	0.12 (0.12-0.13)	0.6 (0.56-0.68)	0.1 (0.09-0.12)	0.53 (0.5-0.59)	0.18 (0.17-0.2)
		**STR**	0.66 (0.63-0.71)	0.18 (0.16-0.21)	0.6 (0.58-0.63)	0.17 (0.16-0.18)	0.53 (0.48-0.61)	0.11 (0.1-0.13)	0.51 (0.48-0.56)	0.25 (0.23-0.27)
		**TUM+STR**	0.7 (0.67-0.74)	0.16 (0.15-0.19)	0.63 (0.61-0.66)	0.12 (0.12-0.13)	0.57 (0.53-0.65)	0.1 (0.09-0.13)	0.53 (0.5-0.59)	0.19 (0.17-0.21)
		**Other**	0.63 (0.6-0.68)	0.17 (0.16-0.2)	0.62 (0.6-0.65)	0.14 (0.13-0.16)	0.55 (0.51-0.62)	0.1 (0.08-0.12)	0.49 (0.46-0.55)	0.21 (0.2-0.24)
		**ALL**	0.68 (0.65-0.73)	0.16 (0.15-0.19)	0.63 (0.61-0.66)	0.11 (0.1-0.12)	0.58 (0.53-0.65)	0.1 (0.09-0.12)	0.51 (0.48-0.56)	0.17 (0.16-0.19)
	**DINO-tcga**	**TUM**	0.68 (0.65-0.73)	0.15 (0.14-0.18)	0.62 (0.61-0.65)	0.13 (0.13-0.15)	0.55 (0.5-0.64)	0.14 (0.12-0.16)	0.54 (0.51-0.59)	0.23 (0.21-0.26)
		**STR**	0.67 (0.64-0.72)	0.18 (0.16-0.2)	0.59 (0.58-0.62)	0.18 (0.17-0.2)	0.54 (0.49-0.62)	0.12 (0.11-0.15)	0.55 (0.52-0.6)	0.26 (0.24-0.29)
		**TUM+STR**	0.67 (0.64-0.72)	0.16 (0.14-0.18)	0.62 (0.6-0.65)	0.13 (0.12-0.14)	0.53 (0.48-0.61)	0.14 (0.12-0.17)	0.54 (0.51-0.59)	0.22 (0.2-0.25)
		**Other**	0.63 (0.6-0.68)	0.18 (0.16-0.2)	0.6 (0.58-0.63)	0.19 (0.18-0.2)	0.53 (0.49-0.61)	0.13 (0.11-0.16)	0.54 (0.52-0.59)	0.26 (0.24-0.3)
		**ALL**	0.65 (0.62-0.7)	0.17 (0.15-0.19)	0.61 (0.59-0.64)	0.14 (0.13-0.15)	0.53 (0.49-0.61)	0.13 (0.12-0.16)	0.55 (0.52-0.6)	0.26 (0.24-0.29)
	**R26-ViT**	**TUM**	0.67 (0.64-0.72)	0.17 (0.15-0.19)	0.6 (0.58-0.63)	0.18 (0.17-0.19)	0.58 (0.54-0.66)	0.14 (0.13-0.17)	0.54 (0.51-0.59)	0.27 (0.25-0.3)
		**STR**	0.67 (0.64-0.72)	0.19 (0.17-0.21)	0.59 (0.57-0.62)	0.2 (0.19-0.22)	0.56 (0.51-0.64)	0.12 (0.11-0.15)	0.57 (0.53-0.62)	0.3 (0.28-0.34)
		**TUM+STR**	0.67 (0.64-0.72)	0.17 (0.15-0.19)	0.6 (0.58-0.63)	0.18 (0.17-0.19)	0.57 (0.52-0.65)	0.15 (0.13-0.18)	0.55 (0.52-0.6)	0.28 (0.26-0.31)
		**Other**	0.64 (0.61-0.69)	0.18 (0.17-0.21)	0.6 (0.58-0.63)	0.19 (0.18-0.2)	0.56 (0.52-0.64)	0.14 (0.13-0.17)	0.57 (0.54-0.62)	0.35 (0.33-0.39)
		**ALL**	0.66 (0.63-0.71)	0.17 (0.16-0.2)	0.6 (0.58-0.63)	0.18 (0.17-0.19)	0.57 (0.52-0.64)	0.15 (0.13-0.17)	0.55 (0.52-0.6)	0.29 (0.27-0.32)
	**Ensemble**	**TUM**	0.69 (0.66-0.74)	0.16 (0.14-0.18)	0.63 (0.61-0.65)	0.14 (0.14-0.15)	0.57 (0.51-0.65)	0.12 (0.1-0.14)	0.56 (0.53-0.61)	0.19 (0.18-0.22)
**CLR**	**Rand**	**TUM**	0.54 (0.49-0.61)	0.08 (0.07-0.11)	0.59 (0.56-0.64)	0.14 (0.13-0.16)	0.6 (0.54-0.69)	0.06 (0.05-0.08)	0.47 (0.43-0.55)	0.2 (0.17-0.24)
		**STR**	0.51 (0.47-0.59)	0.08 (0.07-0.1)	0.55 (0.52-0.6)	0.09 (0.08-0.1)	0.43 (0.35-0.58)	0.05 (0.04-0.08)	0.53 (0.49-0.61)	0.21 (0.19-0.26)
		**TUM+STR**	0.55 (0.49-0.63)	0.08 (0.07-0.1)	0.56 (0.53-0.61)	0.1 (0.09-0.12)	0.53 (0.46-0.65)	0.05 (0.04-0.07)	0.44 (0.4-0.52)	0.23 (0.2-0.28)
		**Other**	0.56 (0.51-0.64)	0.08 (0.07-0.11)	0.52 (0.48-0.57)	0.07 (0.07-0.08)	0.53 (0.47-0.63)	0.04 (0.03-0.06)	0.49 (0.44-0.57)	0.17 (0.15-0.2)
		**ALL**	0.55 (0.5-0.64)	0.08 (0.07-0.11)	0.54 (0.51-0.58)	0.1 (0.09-0.11)	0.56 (0.5-0.68)	0.05 (0.04-0.07)	0.44 (0.39-0.52)	0.2 (0.17-0.24)
	**IM1K**	**TUM**	0.61 (0.56-0.69)	0.08 (0.07-0.1)	0.56 (0.53-0.6)	0.08 (0.07-0.09)	0.44 (0.38-0.56)	0.04 (0.03-0.06)	0.51 (0.46-0.59)	0.15 (0.13-0.19)
		**STR**	0.6 (0.56-0.68)	0.08 (0.07-0.1)	0.56 (0.53-0.6)	0.09 (0.08-0.1)	0.44 (0.38-0.57)	0.04 (0.03-0.06)	0.56 (0.5-0.65)	0.15 (0.13-0.18)

	**TUM+STR**	0.64 (0.59-0.71)	0.08 (0.07-0.1)	0.53 (0.5-0.58)	0.07 (0.06-0.08)	0.42 (0.36-0.54)	0.04 (0.03-0.05)	0.53 (0.48-0.61)	0.11 (0.1-0.14)
	**Other**	0.64 (0.6-0.71)	0.08 (0.07-0.1)	0.6 (0.57-0.64)	0.07 (0.06-0.08)	0.4 (0.32-0.55)	0.05 (0.04-0.06)	0.57 (0.52-0.65)	0.13 (0.11-0.16)
	**ALL**	0.66 (0.62-0.74)	0.08 (0.07-0.1)	0.58 (0.55-0.63)	0.07 (0.07-0.08)	0.42 (0.36-0.51)	0.04 (0.03-0.06)	0.53 (0.48-0.61)	0.13 (0.11-0.16)
**Cam**	**TUM**	0.63 (0.59-0.71)	0.08 (0.07-0.11)	0.54 (0.51-0.59)	0.08 (0.07-0.09)	0.54 (0.47-0.67)	0.05 (0.04-0.07)	0.51 (0.46-0.6)	0.17 (0.15-0.21)
	**STR**	0.59 (0.54-0.67)	0.08 (0.07-0.1)	0.52 (0.49-0.57)	0.11 (0.1-0.13)	0.48 (0.41-0.6)	0.04 (0.03-0.06)	0.51 (0.46-0.59)	0.18 (0.15-0.22)
	**TUM+STR**	0.63 (0.59-0.71)	0.08 (0.07-0.11)	0.5 (0.47-0.55)	0.07 (0.06-0.08)	0.52 (0.45-0.64)	0.05 (0.04-0.06)	0.54 (0.49-0.62)	0.15 (0.13-0.18)
	**Other**	0.62 (0.56-0.71)	0.08 (0.07-0.1)	0.56 (0.53-0.61)	0.09 (0.08-0.1)	0.5 (0.42-0.62)	0.04 (0.03-0.06)	0.55 (0.5-0.62)	0.16 (0.13-0.19)
	**ALL**	0.67 (0.63-0.74)	0.08 (0.07-0.1)	0.52 (0.49-0.57)	0.07 (0.06-0.08)	0.53 (0.46-0.65)	0.04 (0.03-0.06)	0.54 (0.49-0.63)	0.14 (0.12-0.17)
**Sub**	**TUM**	0.6 (0.55-0.68)	0.09 (0.07-0.11)	0.58 (0.56-0.63)	0.11 (0.1-0.13)	0.46 (0.39-0.6)	0.06 (0.04-0.08)	0.49 (0.45-0.58)	0.21 (0.18-0.26)
	**STR**	0.56 (0.51-0.65)	0.09 (0.07-0.11)	0.55 (0.52-0.61)	0.11 (0.1-0.13)	0.41 (0.33-0.54)	0.04 (0.03-0.06)	0.48 (0.43-0.57)	0.19 (0.16-0.24)
	**TUM+STR**	0.6 (0.55-0.68)	0.09 (0.07-0.11)	0.58 (0.55-0.63)	0.12 (0.11-0.14)	0.44 (0.38-0.55)	0.06 (0.04-0.08)	0.49 (0.44-0.58)	0.21 (0.18-0.26)
	**Other**	0.61 (0.56-0.69)	0.08 (0.07-0.11)	0.57 (0.54-0.62)	0.1 (0.09-0.12)	0.46 (0.4-0.57)	0.04 (0.03-0.05)	0.51 (0.45-0.6)	0.19 (0.16-0.23)
	**ALL**	0.6 (0.54-0.68)	0.09 (0.07-0.11)	0.57 (0.54-0.62)	0.12 (0.11-0.14)	0.43 (0.37-0.54)	0.05 (0.04-0.07)	0.51 (0.46-0.59)	0.21 (0.18-0.25)
**Ciga**	**TUM**	0.61 (0.56-0.69)	0.08 (0.07-0.11)	0.53 (0.5-0.58)	0.06 (0.05-0.07)	0.48 (0.41-0.62)	0.04 (0.03-0.06)	0.57 (0.52-0.66)	0.13 (0.11-0.16)
	**STR**	0.64 (0.6-0.72)	0.08 (0.07-0.1)	0.57 (0.55-0.62)	0.09 (0.08-0.11)	0.45 (0.39-0.57)	0.04 (0.03-0.05)	0.53 (0.49-0.62)	0.18 (0.16-0.23)
	**TUM+STR**	0.62 (0.58-0.7)	0.08 (0.07-0.1)	0.52 (0.5-0.57)	0.05 (0.05-0.06)	0.5 (0.43-0.62)	0.04 (0.03-0.06)	0.54 (0.5-0.62)	0.16 (0.14-0.2)
	**Other**	0.64 (0.59-0.72)	0.08 (0.06-0.1)	0.59 (0.57-0.64)	0.09 (0.08-0.1)	0.5 (0.45-0.6)	0.04 (0.03-0.05)	0.56 (0.51-0.64)	0.17 (0.15-0.21)
	**ALL**	0.62 (0.57-0.71)	0.08 (0.07-0.11)	0.53 (0.5-0.58)	0.05 (0.05-0.06)	0.49 (0.42-0.61)	0.04 (0.03-0.05)	0.55 (0.5-0.64)	0.19 (0.16-0.23)
**Retccl**	**TUM**	0.63 (0.59-0.71)	0.08 (0.07-0.11)	0.57 (0.54-0.62)	0.07 (0.06-0.08)	0.48 (0.41-0.59)	0.04 (0.03-0.05)	0.53 (0.48-0.61)	0.07 (0.06-0.09)
	**STR**	0.57 (0.52-0.65)	0.09 (0.07-0.11)	0.58 (0.55-0.62)	0.09 (0.08-0.1)	0.45 (0.38-0.56)	0.04 (0.03-0.06)	0.55 (0.5-0.64)	0.11 (0.09-0.14)
	**TUM+STR**	0.64 (0.6-0.71)	0.08 (0.07-0.1)	0.58 (0.55-0.62)	0.07 (0.06-0.08)	0.49 (0.43-0.62)	0.04 (0.03-0.05)	0.56 (0.52-0.64)	0.09 (0.08-0.12)
	**Other**	0.65 (0.61-0.73)	0.08 (0.07-0.1)	0.58 (0.55-0.62)	0.08 (0.08-0.1)	0.48 (0.42-0.6)	0.04 (0.03-0.06)	0.58 (0.54-0.67)	0.1 (0.09-0.13)
	**ALL**	0.65 (0.6-0.72)	0.08 (0.07-0.1)	0.58 (0.55-0.63)	0.07 (0.06-0.08)	0.48 (0.41-0.6)	0.04 (0.03-0.05)	0.59 (0.54-0.67)	0.09 (0.08-0.11)
**DINO-dachs**	**TUM**	0.61 (0.57-0.69)	0.09 (0.07-0.11)	0.58 (0.56-0.63)	0.07 (0.07-0.09)	0.49 (0.42-0.63)	0.04 (0.03-0.06)	0.51 (0.46-0.58)	0.12 (0.1-0.15)
	**STR**	0.6 (0.55-0.67)	0.08 (0.07-0.1)	0.58 (0.55-0.63)	0.08 (0.08-0.1)	0.45 (0.37-0.58)	0.04 (0.03-0.06)	0.55 (0.5-0.63)	0.17 (0.14-0.2)
	**TUM+STR**	0.62 (0.57-0.7)	0.08 (0.07-0.11)	0.59 (0.56-0.64)	0.07 (0.06-0.08)	0.54 (0.47-0.67)	0.04 (0.03-0.06)	0.49 (0.44-0.57)	0.13 (0.11-0.16)
	**Other**	0.62 (0.57-0.7)	0.08 (0.07-0.1)	0.61 (0.58-0.66)	0.08 (0.07-0.09)	0.52 (0.45-0.65)	0.04 (0.03-0.05)	0.51 (0.47-0.6)	0.14 (0.13-0.18)
	**ALL**	0.63 (0.58-0.7)	0.08 (0.07-0.11)	0.6 (0.57-0.65)	0.06 (0.06-0.07)	0.53 (0.46-0.64)	0.04 (0.03-0.05)	0.45 (0.4-0.53)	0.12 (0.1-0.14)

**DINO-tcga**	**TUM**	0.61 (0.56-0.69)	0.09 (0.07-0.11)	0.54 (0.51-0.59)	0.08 (0.07-0.1)	0.51 (0.44-0.64)	0.05 (0.04-0.07)	0.52 (0.47-0.61)	0.17 (0.15-0.22)
	**STR**	0.58 (0.53-0.66)	0.09 (0.07-0.11)	0.58 (0.55-0.63)	0.09 (0.08-0.1)	0.45 (0.39-0.56)	0.05 (0.04-0.07)	0.54 (0.49-0.62)	0.18 (0.16-0.23)
	**TUM+STR**	0.6 (0.55-0.68)	0.08 (0.07-0.11)	0.55 (0.52-0.6)	0.08 (0.07-0.09)	0.51 (0.44-0.64)	0.05 (0.04-0.07)	0.53 (0.48-0.61)	0.17 (0.14-0.21)
	**Other**	0.63 (0.58-0.7)	0.08 (0.07-0.1)	0.6 (0.57-0.65)	0.09 (0.08-0.11)	0.53 (0.47-0.65)	0.05 (0.04-0.07)	0.58 (0.53-0.65)	0.18 (0.16-0.23)
	**ALL**	0.61 (0.56-0.7)	0.09 (0.07-0.11)	0.55 (0.52-0.61)	0.08 (0.07-0.09)	0.5 (0.43-0.63)	0.05 (0.04-0.07)	0.57 (0.52-0.66)	0.19 (0.16-0.24)
**R26-ViT**	**TUM**	0.62 (0.58-0.7)	0.08 (0.07-0.11)	0.55 (0.52-0.59)	0.09 (0.09-0.11)	0.51 (0.44-0.65)	0.05 (0.04-0.07)	0.53 (0.48-0.62)	0.21 (0.18-0.26)
	**STR**	0.62 (0.57-0.69)	0.08 (0.07-0.1)	0.57 (0.54-0.62)	0.09 (0.08-0.11)	0.53 (0.46-0.63)	0.04 (0.03-0.06)	0.57 (0.52-0.66)	0.21 (0.18-0.26)
	**TUM+STR**	0.63 (0.59-0.71)	0.08 (0.07-0.1)	0.55 (0.52-0.59)	0.09 (0.08-0.11)	0.49 (0.43-0.62)	0.05 (0.04-0.07)	0.54 (0.48-0.63)	0.21 (0.18-0.27)
	**Other**	0.63 (0.58-0.71)	0.08 (0.07-0.1)	0.6 (0.57-0.65)	0.09 (0.08-0.11)	0.51 (0.44-0.63)	0.05 (0.04-0.07)	0.59 (0.54-0.67)	0.24 (0.21-0.29)
	**ALL**	0.63 (0.59-0.71)	0.08 (0.07-0.1)	0.54 (0.51-0.59)	0.09 (0.08-0.11)	0.5 (0.43-0.61)	0.05 (0.04-0.07)	0.53 (0.48-0.62)	0.22 (0.19-0.27)
**Ensemble**	**TUM**	0.64 (0.59-0.72)	0.08 (0.07-0.11)	0.56 (0.54-0.61)	0.08 (0.07-0.09)	0.49 (0.42-0.61)	0.05 (0.04-0.07)	0.54 (0.48-0.63)	0.15 (0.13-0.18)

CHR=clinical high risk; CLR=clinical low risk; BS=Brier score; TUM=tumor; STR=stroma; TUM+STR=tumor and stroma; other=stroma, lymphocytes and mucus; ALL=tumor, stroma, lymphocytes and mucus.

Supplementary Table 5: Discrimination and calibration of the ensemble model of the survival curve approach with and without recalibration. For all metrics, the 95% confidence intervals are reported next to the expected values. Arrows indicate whether high or low values are better.

UICC risk group	Re-cali-braton	DACHS		MCO		TCGA		Graz	
		C-index ↑	IBS ↓	C-index ↑	IBS ↓	C-index ↑	IBS ↓	C-index ↑	IBS ↓
**CHR**	no	0.69 (0.67-0.74)	0.17 (0.17-0.18)	0.64 (0.62-0.67)	0.17 (0.17-0.18)	0.6 (0.56-0.69)	0.11 (0.09-0.12)	0.55 (0.53-0.61)	0.2 (0.19-0.21)
	yes	0.69 (0.67-0.74)	0.17 (0.17-0.18)	0.64 (0.62-0.67)	0.18 (0.17-0.19)	0.6 (0.56-0.69)	0.11 (0.1-0.13)	0.55 (0.53-0.61)	0.2 (0.19-0.22)
**CLR**	no	0.64 (0.59-0.71)	0.09 (0.09-0.1)	0.59 (0.56-0.64)	0.09 (0.09-0.1)	0.51 (0.44-0.65)	0.05 (0.05-0.07)	0.52 (0.47-0.61)	0.18 (0.18-0.2)
	yes	0.64 (0.59-0.71)	0.09 (0.09-0.1)	0.59 (0.56-0.64)	0.09 (0.09-0.1)	0.51 (0.44-0.65)	0.05 (0.05-0.07)	0.52 (0.47-0.61)	0.16 (0.15-0.19)

CHR=clinical high risk; CLR=clinical low risk; IBS=integrated Brier score.

Supplementary Table 6: Coefficients, P-values and hazard ratios with 95% confidence intervals of all input variables of the clinical and combined models including the mortality score.

UICC risk group	Covariates	Clinical models			Combined models		
		Coefficient	P-value	Hazard ratio (95% CI)	Coefficient	P-value	Hazard ratio (95% CI)
**CHR**	Age < 60	-0.446	0.002	0.640 (0.484-0.848)	-0.433	0.003	0.649 (0.490-0.859)
	Age 60-69	-0.135	0.300	0.873 (0.676-1.128)	-0.111	0.398	0.895 (0.693-1.157)
	Age 70-79	+0.036	0.780	1.037 (0.804-1.337)	-0.004	0.977	0.996 (0.772-1.285)
	Age > 80	+0.690	<0.0005	1.994 (1.511-2.631)	+0.700	<0.0005	2.013 (1.525-2.657)
	T1	-0.031	0.939	0.970 (0.438-2.145)	+0.114	0.781	1.121 (0.500-2.514)
	T2	-0.481	0.026	0.618 (0.405-0.945)	-0.364	0.097	0.695 (0.452-1.068)
	T3	-0.008	0.957	0.992 (0.746-1.319)	+0.013	0.928	1.013 (0.762-1.348)
	T4	+0.192	0.200	1.212 (0.903-1.626)	+0.112	0.456	1.119 (0.833-1.503)
	N0	-0.330	0.050	0.719 (0.517-1.000)	-0.248	0.143	0.780 (0.560-1.087)
	N1	-0.145	0.289	0.865 (0.662-1.131)	-0.119	0.385	0.888 (0.680-1.161)
	N2	+0.297	0.029	1.346 (1.031-1.758)	+0.234	0.087	1.264 (0.967-1.652)
	M+	+1.240	<0.0005	3.455 (2.895-4.124)	+1.189	<0.0005	3.282 (2.746-3.924)
	Mortality score	NA	NA	NA	0.032	<0.0005	1.033 (1.024-1.042)
**CLR**	Age < 60	-0.351	0.056	0.704 (0.491-1.009)	-0.330	0.074	0.719 (0.501-1.032)
	Age 60-69	-0.345	0.025	0.708 (0.524-0.958)	-0.342	0.026	0.711 (0.526-0.961)
	Age 70-79	-0.002	0.989	0.998 (0.756-1.317)	-0.001	0.997	0.999 (0.757-1.319)
	Age > 80	0.783	<0.0005	2.187 (1.617-2.960)	+0.758	<0.0005	2.133 (1.575-2.888)
	T1	-0.203	0.316	0.817 (0.549-1.214)	-0.151	0.461	0.860 (0.576-1.284)
	T2	-0.126	0.423	0.882 (0.648-1.199)	-0.108	0.493	0.898 (0.660-1.222)
	T3	0.197	0.182	1.217 (0.912-1.625)	+0.158	0.286	1.171 (0.876-1.566)
	Mortality score	NA	NA	NA	+0.032	0.005	1.032 (1.009-1.056)

CI=confidence interval; CHR=clinical high risk; CLR=clinical low risk.

Supplementary Table 7: Ablation study for the clinical and combined model including the mortality score: variants of the clinical model (left) and its combination with the mortality score derived from the ensemble image model with recalibration on external test sets (right). C-indices with 95% confidence intervals are shown. As in univariate (Supplementary Table 2) and multivariable analyses (Supplementary Figure 20), age was provided in groups (<60, 60-69, 70-79, >80) and TNM stages as T1, T2, N1 etc.

UICC risk group	Covariates	Clinical model				+ Mortality score			
		DACHS	MCO	TCGA	Graz	DACHS	MCO	TCGA	Graz
CHR	Age groups, TNM stages	0.752 (0.73-0.793)	0.722 (0.707-0.747)	0.703 (0.664-0.769)	0.654 (0.63-0.708)	0.773 (0.751-0.812)	0.741 (0.728-0.765)	0.704 (0.663-0.784)	0.646 (0.620-0.700)
	TNM stages	0.707 0.684-0.75)	0.7 (0.684-0.728)	0.69 (0.656-0.756)	0.636 (0.609-0.695)	0.741 (0.719-0.783)	0.721 (0.706-0.75)	0.684 (0.638-0.762)	0.625 (0.595-0.682)
	Age groups	0.628 (0.601-0.68)	0.557 (0.538-0.588)	0.618 (0.579-0.685)	0.568 (0.539-0.626)	0.717 (0.693-0.762)	0.653 (0.639-0.685)	0.635 (0.589-0.717)	0.593 (0.565-0.652)
CLR	Age groups, T stage	0.651 (0.6-0.731)	0.677 (0.652-0.723)	0.68 0.617-0.788)	0.64 (0.599-0.727)	0.677 (0.63-0.759)	0.683 (0.657-0.727)	0.639 (0.578-0.752)	0.683 (0.638-0.762)
	T stage	0.56 (0.526-0.622)	0.544 (0.52-0.583)	0.515 (0.463-0.615)	0.478 (0.44-0.555)	0.627 (0.584-0.705)	0.593 (0.567-0.64)	0.492 (0.424-0.631)	0.521 (0.479-0.612)
	Age groups	0.641 (0.598-0.725)	0.683 (0.659-0.723)	0.701 (0.649-0.782)	0.68 (0.637-0.756)	0.672 (0.626-0.754)	0.688 (0.661-0.733)	0.645 (0.587-0.756)	0.69 (0.649-0.759)

CHR=clinical high-risk; CLR=clinical low-risk.

Supplementary Table 8: Comparison of clinical, image and combined models of the survival curve approach complemented by the image and combined models of the binary approach. C-indices with 95% confidence intervals on internal and external test sets are shown. Best results are highlighted in bold.

UICC risk group	Model	DACHS	MCO	TCGA	Graz
**CHR**	Clinical model (Age groups, TNM stages)	0.75 (0.73-0.79)	0.72 (0.71-0.75)	**0.70 (0.66-0.77)**	**0.65 (0.63-0.71)**
	Image model (Ensemble, Curve)	0.69 (0.67-0.74)	0.64 (0.62-0.67)	0.60 (0.56-0.69)	0.55 (0.53-0.61)
	Image model (Ensemble, Binary)	0.69 (0.64-0.72)	0.63 (0.58-0.64)	0.57 (0.54-0.66)	0.56 (0.51-0.59)
	Combined model (Mortality score, age groups, TNM stages)	**0.77 (0.75-0.81)**	**0.74 (0.73-0.76)**	**0.70 (0.66-0.78)**	**0.65 (0.62-0.70)**
	Combined model (Ensemble risk score, age groups, TNM stages)	**0.77 (0.75-0.81)**	**0.74 (0.72-0.76)**	0.69 (0.65-0.77)	**0.65 (0.62-0.7)**
**CLR**	Clinical model (Age groups, T stage)	0.65 (0.60-0.73)	**0.68 (0.65-0.72)**	**0.68 (0.63-0.79)**	0.64 (0.60-0.73)
	Image model (Ensemble, Curve)	0.64 (0.59-0.71)	0.59 (0.56-0.64)	0.51 (0.44-0.65)	0.52 (0.47-0.61)
	Image model (Ensemble, Binary)	0.64 (0.58-0.7)	0.56 (0.52-0.59)	0.49 (0.44-0.64)	0.54 (0.48-0.62)
	Combined model (Mortality score, age groups, T stage)	0.68 (0.63-0.76)	**0.68 (0.66-0.73)**	0.64 (0.58-0.75)	0.68 (0.64-0.76)
	Combined model (Ensemble risk score, age groups, T stage)	**0.69 (0.64-0.77)**	0.67 (0.65-0.72)	0.67 (0.61-0.78)	**0.7 (0.65-0.79)**

CHR=clinical high risk; CLR=clinical low risk.

Supplementary Table 9: TRIPOD Checklist: Prediction Model Development and Validation

Section/Topic			Checklist Item	Page
**Title and abstract**				
Title	1	D;V	Identify the study as developing and/or validating a multivariable prediction model, the target population, and the outcome to be predicted.	1
Abstract	2	D;V	Provide a summary of objectives, study design, setting, participants, sample size, predictors, outcome, statistical analysis, results, and conclusions.	1
**Introduction**				
Background and objectives	3a	D;V	Explain the medical context (including whether diagnostic or prognostic) and rationale for developing or validating the multivariable prediction model, including references to existing models.	1
	3b	D;V	Specify the objectives, including whether the study describes the development or validation of the model or both.	1-2
**Methods**				
Source of data	4a	D;V	Describe the study design or source of data (e.g., randomized trial, cohort, or registry data), separately for the development and validation data sets, if applicable.	2-3,7-8
	4b	D;V	Specify the key study dates, including start of accrual; end of accrual; and, if applicable, end of follow-up.	8
Participants	5a	D;V	Specify key elements of the study setting (e.g., primary care, secondary care, general population) including number and location of centres.	8
	5b	D;V	Describe eligibility criteria for participants.	8
	5c	D;V	Give details of treatments received, if relevant.	NA
Outcome	6a	D;V	Clearly define the outcome that is predicted by the prediction model, including how and when assessed.	9
	6b	D;V	Report any actions to blind assessment of the outcome to be predicted.	10
Predictors	7a	D;V	Clearly define all predictors used in developing or validating the multivariable prediction model, including how and when they were measured.	8, 10
	7b	D;V	Report any actions to blind assessment of predictors for the outcome and other predictors.	10
Sample size	8	D;V	Explain how the study size was arrived at.	8, Supp. Figure 1
Missing data	9	D;V	Describe how missing data were handled (e.g., complete-case analysis, single imputation, multiple imputation) with details of any imputation method.	10
Statistical analysis methods	10a	D	Describe how predictors were handled in the analyses.	10
	10b	D	Specify type of model, all model-building procedures (including any predictor selection), and method for internal validation.	7-10
	10c	V	For validation, describe how the predictions were calculated.	9
	10d	D;V	Specify all measures used to assess model performance and, if relevant, to compare multiple models.	10
	10e	V	Describe any model updating (e.g., recalibration) arising from the validation, if done.	9,7
Risk groups	11	D;V	Provide details on how risk groups were created, if done.	9-10, Supp. Figure 13 and 18
Development vs. validation	12	V	For validation, identify any differences from the development data in setting, eligibility criteria, outcome, and predictors.	Table 1, Figure 1, Supp. Table 2
**Results**				
Participants	13a	D;V	Describe the flow of participants through the study, including the number of participants with and without the outcome and, if applicable, a summary of the follow-up time. A diagram may be helpful.	Supp. Figure 1, Figure 1, Table 1

	13b	D;V	Describe the characteristics of the participants (basic demographics, clinical features, available predictors), including the number of participants with missing data for predictors and outcome.	Figure 1, Supp. Table 2
	13c	V	For validation, show a comparison with the development data of the distribution of important variables (demographics, predictors and outcome).	Figure 1, Supp. Table 2
Model development	14a	D	Specify the number of participants and outcome events in each analysis.	Figure 1
	14b	D	If done, report the unadjusted association between each candidate predictor and outcome.	Supp. Table 2
Model specification	15a	D	Present the full prediction model to allow predictions for individuals (i.e., all regression coefficients, and model intercept or baseline survival at a given time point).	Supp. Table 6
	15b	D	Explain how to the use the prediction model.	8-10
Model performance	16	D;V	Report performance measures (with CIs) for the prediction model.	Figure 3, Supp. Table 3 and 4, Table 2
Model-updating	17	V	If done, report the results from any model updating (i.e., model specification, model performance).	Supp. Table 5, Supp. Figure 15 and 16
**Discussion**				
Limitations	18	D;V	Discuss any limitations of the study (such as nonrepresentative sample, few events per predictor, missing data).	7
Interpretation	19a	V	For validation, discuss the results with reference to performance in the development data, and any other validation data.	6-7
	19b	D;V	Give an overall interpretation of the results, considering objectives, limitations, results from similar studies, and other relevant evidence.	7
Implications	20	D;V	Discuss the potential clinical use of the model and implications for future research.	6-7
**Other information**				
Supplementary information	21	D;V	Provide information about the availability of supplementary resources, such as study protocol, Web calculator, and data sets.	10-11
Funding	22	D;V	Give the source of funding and the role of the funders for the present study.	11

*Items relevant only to the development of a prediction model are denoted by D, items relating solely to a validation of a prediction model are denoted by V, and items relating to both are denoted D;V. We recommend using the TRIPOD Checklist in conjunction with the TRIPOD Explanation and Elaboration document.

Supplementary Table 10: Performance of the subtyper. Metrics are reported with 95% confidence intervals. Due to blinded validation of the Graz cohort, the Digital Biomarkers for Oncology group had no access to the slides/tiles and the subtyper could not be evaluated on this cohort.

Test sets	N_{ALL}/N_{TUM}	Acc_{TUM} [%]	Acc_{ALL} [%]
NCT-CRC-HE-100K (internal test set)	16,667/2,386	99.25 (98.89-99.58)	92.61 (92.22-93.02)
CRC-VAL-HE-7K	7,180/1,233	99.03 (98.43-99.53)	81.89 (81.00-82.79)
DACHS	4,114/1,419	99.58 (99.21-99.86)	98.47 (98.08-98.83)
TCGA-H	13,693/1,832	91.65 (90.35-92.90)	91.62 (91.15-92.06)
TCGA-GPCC	7,138/1,332	97.30 (96.40-98.14)	87.80 (87.04-88.54)
TCGA-CH	5,052/1,383	83.73 (81.82-85.65)	92.76 (92.04-93.47)
MCO	33,221/14,211	79.35 (78.69-80.01)	82.91 (82.52-83.31)

TCGA submitting site codes: H=Harvard; GPCC=Greater Poland Cancer Center; CH=Christiana Healthcare; N_{ALL}/N_{TUM}=number of tiles of all tissue types/tumor type; Acc_{TUM}/Acc_{ALL}=accuracy of tumor/all tissue type classes.

Supplementary Figures

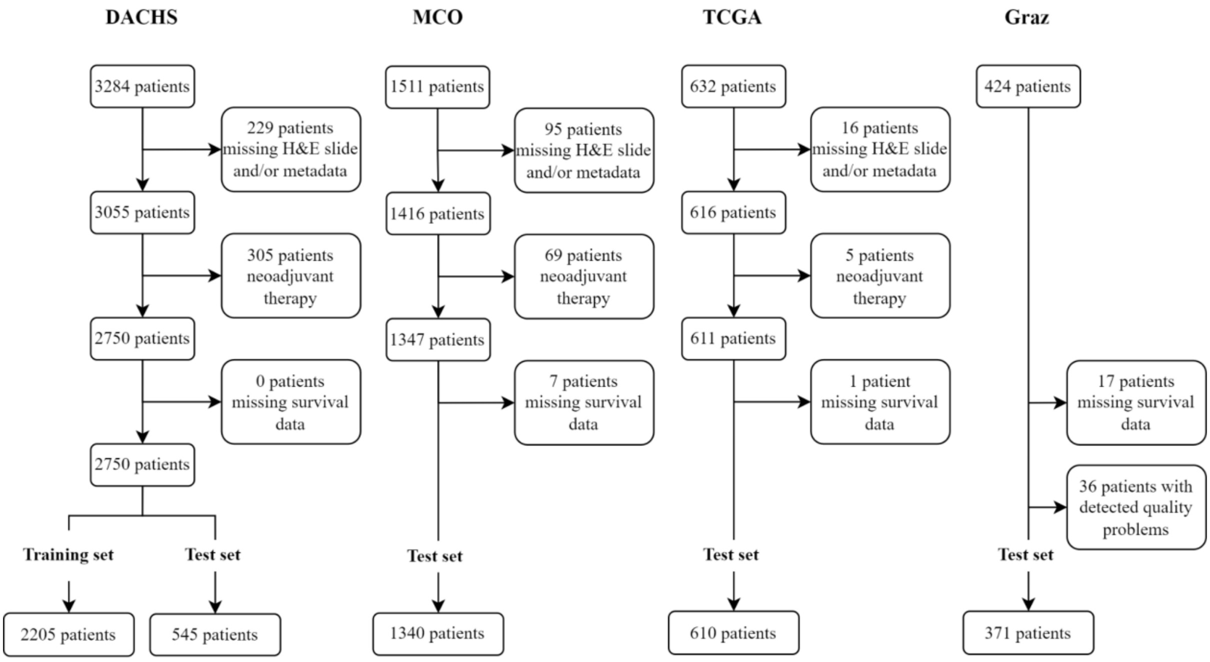

Supplementary Figure 1: Flowchart of patient inclusion. The Graz cohort was evaluated in a blinded manner. All provided 424 patients had a corresponding H&E slide; however, the information of neoadjuvant therapy was not available. We further had to exclude 36 patients due to pipeline problems that were detected by automatic sanity checks in the blinded validation: tiling problems (30 patients) and problems in the tissue type classification (e.g. no tumor detected, 6 patients).

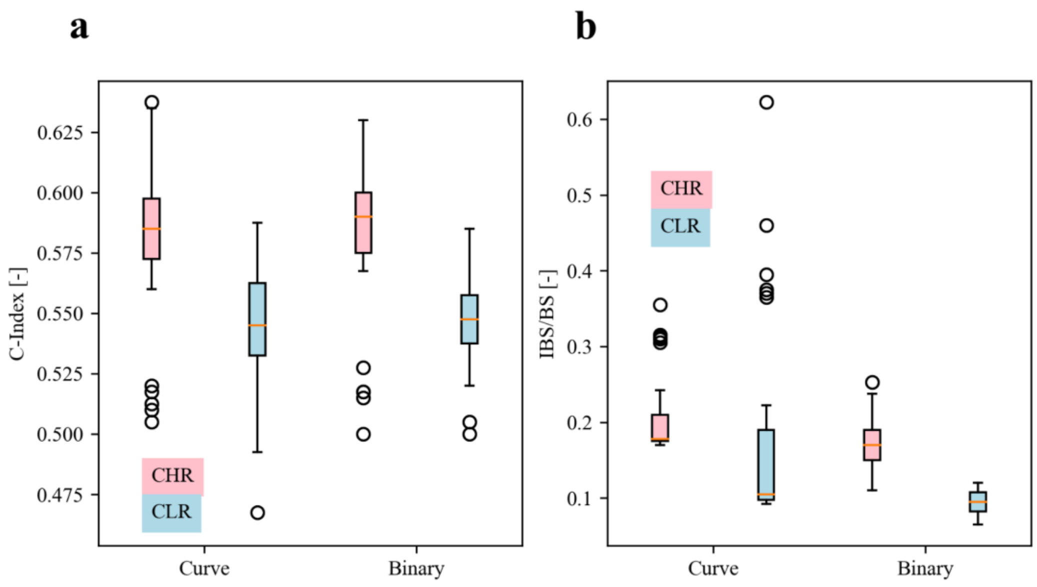

Supplementary Figure 2: Impact of risk prediction. a) Impact of the risk prediction on the discrimination (C-index) and **b)** of the calibration (IBS/BS) for the CHR and CLR subcohorts. For both approaches, the survival curve based (Curve) and the binary approach (Binary), we trained models with five different input tissue types and eight pre-trained feature extractors (5x8=40 models for each approach). For each model we calculated the mean C-index and mean IBS/BS across the four CHR and CLR subcohorts, separately. One box plot shows the performance distribution of 40 models. For the results of all individual models on all test sets we refer to Supplementary Table 3 and 4. Note that IBS and BS are not directly comparable. Box plot elements: the box extends from the first quartile to the third quartile of the data, with a line at the median. The whiskers extend from the box by 1.5x the inter-quartile range. Flier points are those past the end of the whiskers.
CHR=clinical high risk; CLR=clinical low risk; IBS=integrated Brier score; BS=Brier score.

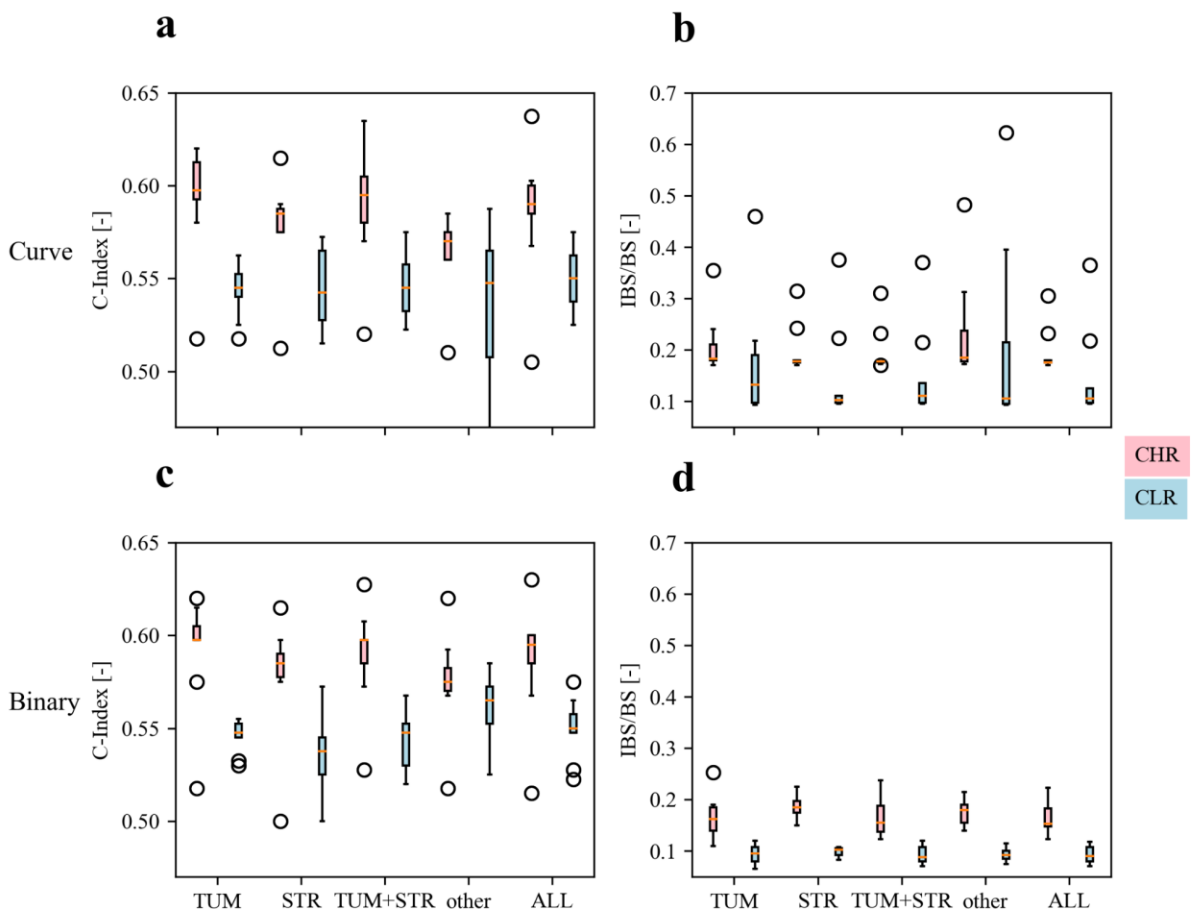

Supplementary Figure 3: Impact of input tissue type. a) Impact on the discrimination (C-index) and **b)** on the calibration (IBS/BS) of the survival curve approach for the CHR and CLR subcohorts, separately. **c)** Impact on the discrimination (C-index) and **d)** on the calibration (IBS/BS) of the binary approach for the CHR and CLR subcohorts, separately. For both approaches, the survival curve (Curve) and the binary approach (Binary), we trained models with five different input tissue types and eight pre-trained feature extractors (5x8=40 models for each approach) and for each model we calculated the mean C-index and mean IBS/BS across the four CHR and CLR subcohorts, separately. One box plot shows the performance distribution of eight models (the eight different feature extractors). For the results of all individual models on all test sets we refer to Supplementary Table 3 and 4. Note that IBS and BS are not directly comparable. Box plot elements: The box extends from the first quartile to the third quartile of the data, with a line at the median. The whiskers extend from the box by 1.5x the inter-quartile range. Flier points are those past the end of the whiskers.
CHR=clinical high risk; CLR=clinical low risk; IBS=integrated Brier score; BS=Brier score; TUM=tumor; STR= stroma; TUM+STR=tumor and stroma; other=stroma, lymphocytes and mucus; ALL=tumor, stroma, lymphocytes and mucus.

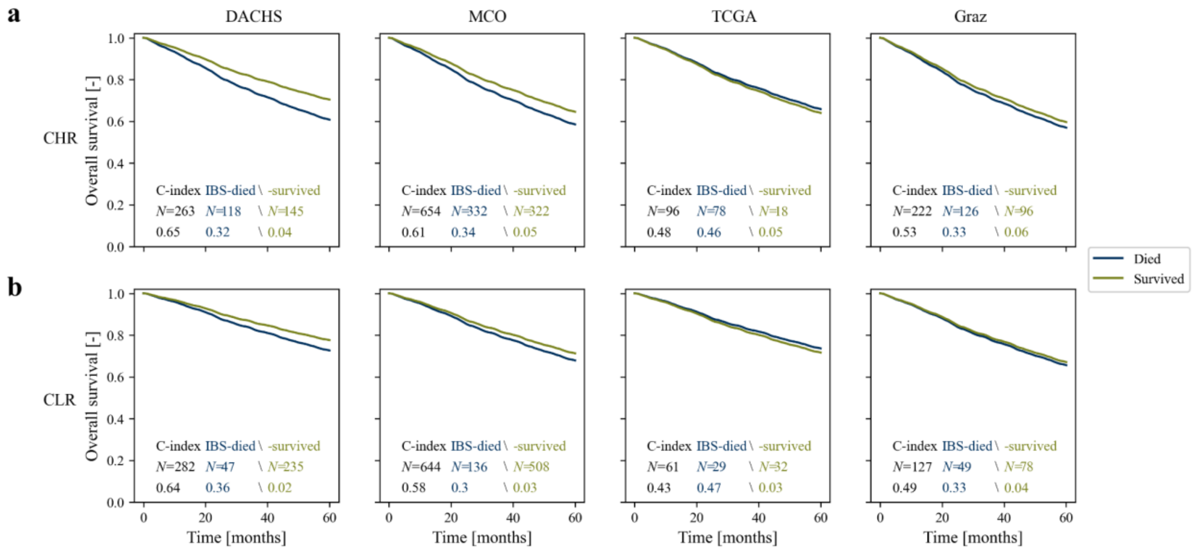

Supplementary Figure 4: Mean predicted survival curves of the IM1K model (without recalibration). a, Mean predicted survival curves of the CHR subcohorts. **b,** Mean predicted survival curves of the CLR subcohorts. Note that all curves and metrics reported in the subfigures were calculated without censored cases. CHR=clinical high risk; CLR=clinical low risk; IBS=integrated Brier score.

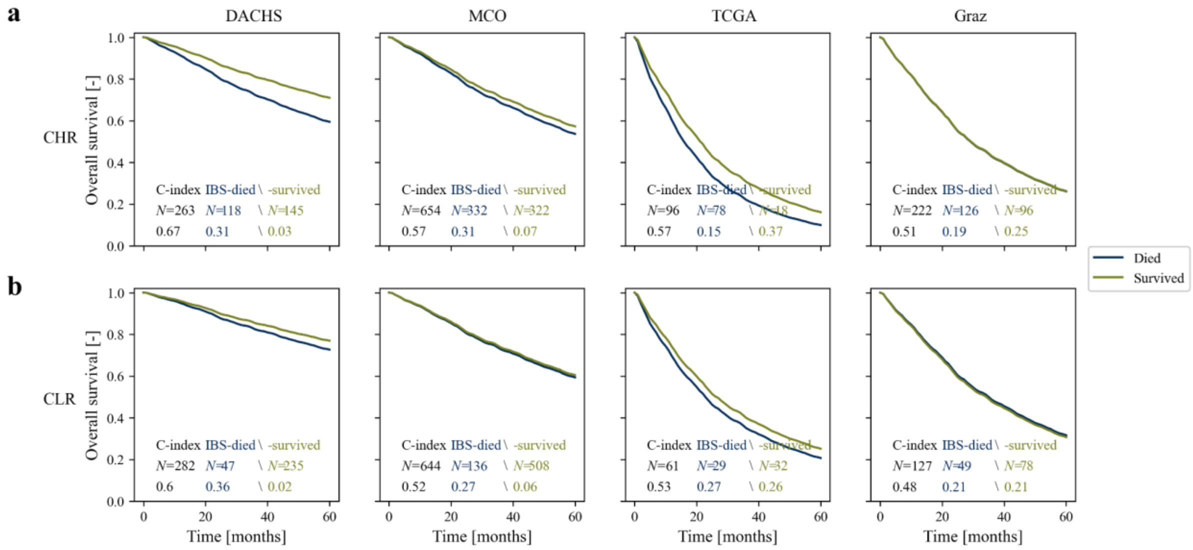

Supplementary Figure 5: Mean predicted survival curves of the Cam model (without recalibration). a, Mean predicted survival curves of the CHR subcohorts. **b,** Mean predicted survival curves of the CLR subcohorts. Note that all curves and metrics reported in the subfigures were calculated without censored cases. CHR=clinical high risk; CLR=clinical low risk; IBS=integrated Brier score.

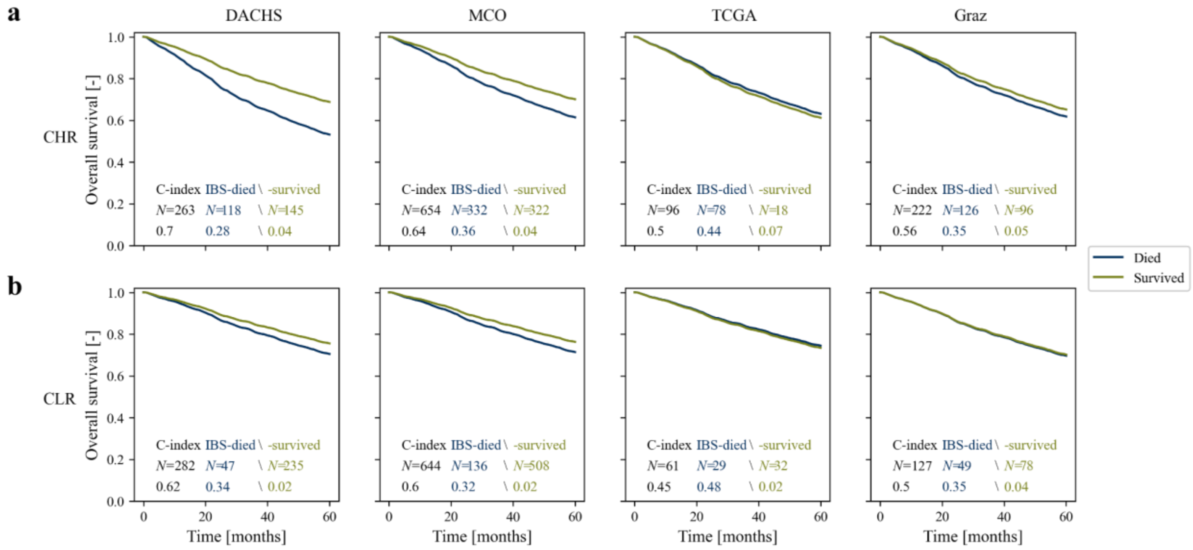

Supplementary Figure 6: Mean predicted survival curves of the Sub model (without recalibration). a, Mean predicted survival curves of the CHR subcohorts. **b,** Mean predicted survival curves of the CLR subcohorts. Note that all curves and metrics reported in the subfigures were calculated without censored cases. CHR=clinical high risk; CLR=clinical low risk; IBS=integrated Brier score.

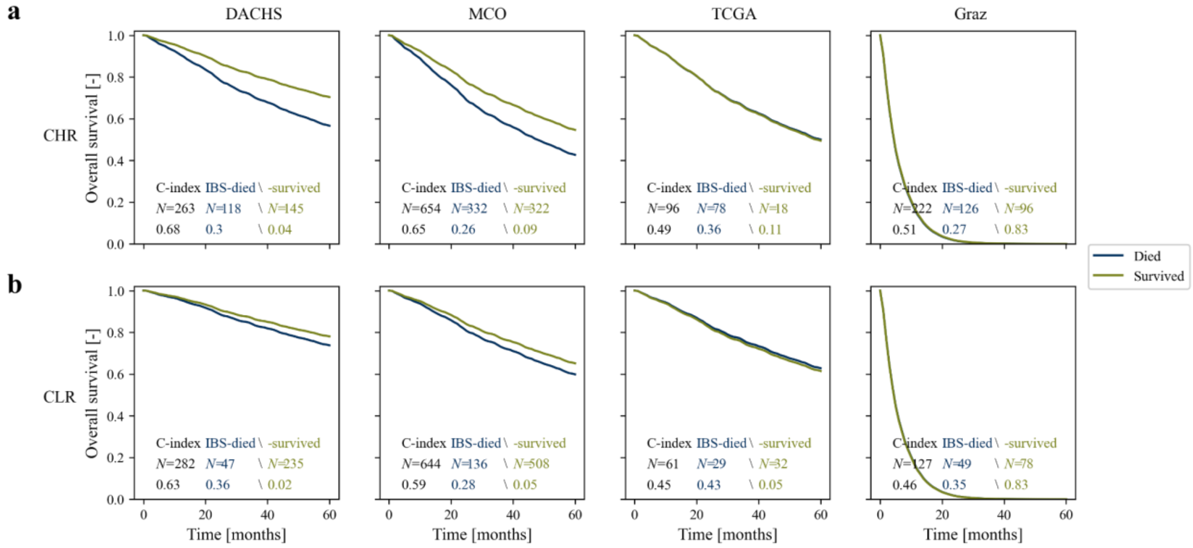

Supplementary Figure 7: Mean predicted survival curves of the DINO-dachs model (without recalibration). a, Mean predicted survival curves of the CHR subcohorts. **b,** Mean predicted survival curves of the CLR subcohorts. Note that all curves and metrics reported in the subfigures were calculated without censored cases. CHR=clinical high risk; CLR=clinical low risk; IBS=integrated Brier score.

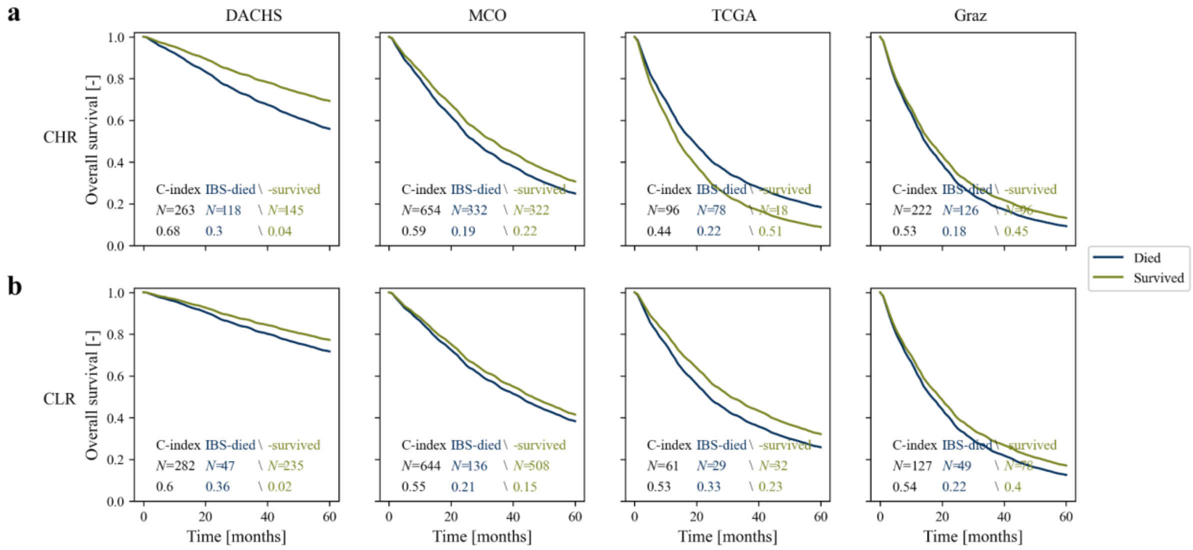

Supplementary Figure 8: Mean predicted survival curves of the Ciga model (without recalibration). a, Mean predicted survival curves of the CHR subcohorts. **b,** Mean predicted survival curves of the CLR subcohorts. Note that all curves and metrics reported in the subfigures were calculated without censored cases. CHR=clinical high risk; CLR=clinical low risk; IBS=integrated Brier score.

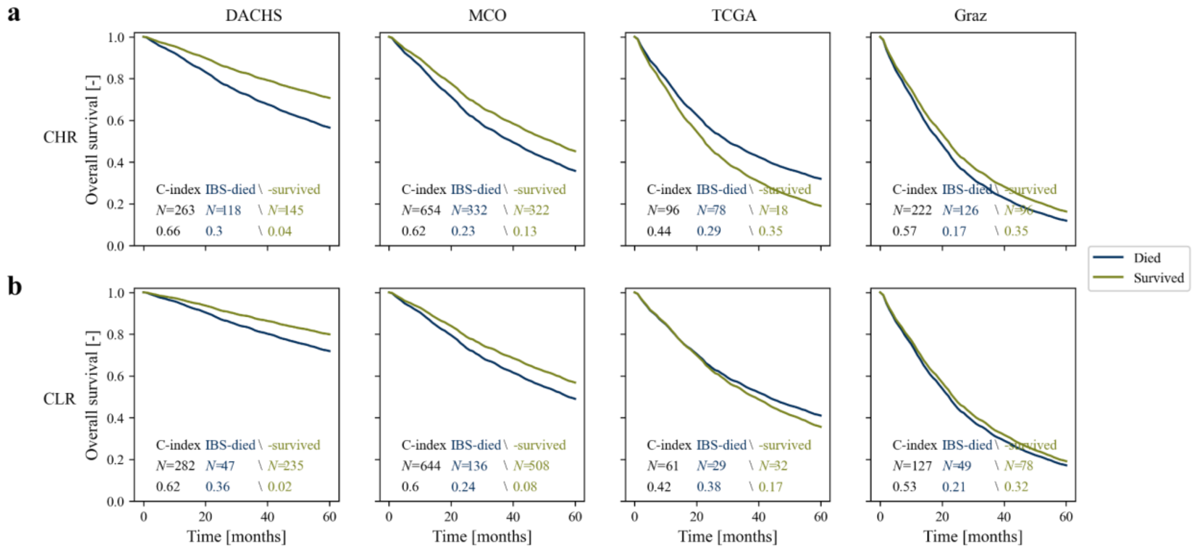

Supplementary Figure 9: Mean predicted survival curves of the Retccl model (without recalibration). a, Mean predicted survival curves of the CHR subcohorts. **b,** Mean predicted survival curves of the CLR subcohorts. Note that all curves and metrics reported in the subfigures were calculated without censored cases. CHR=clinical high risk; CLR=clinical low risk; IBS=integrated Brier score.

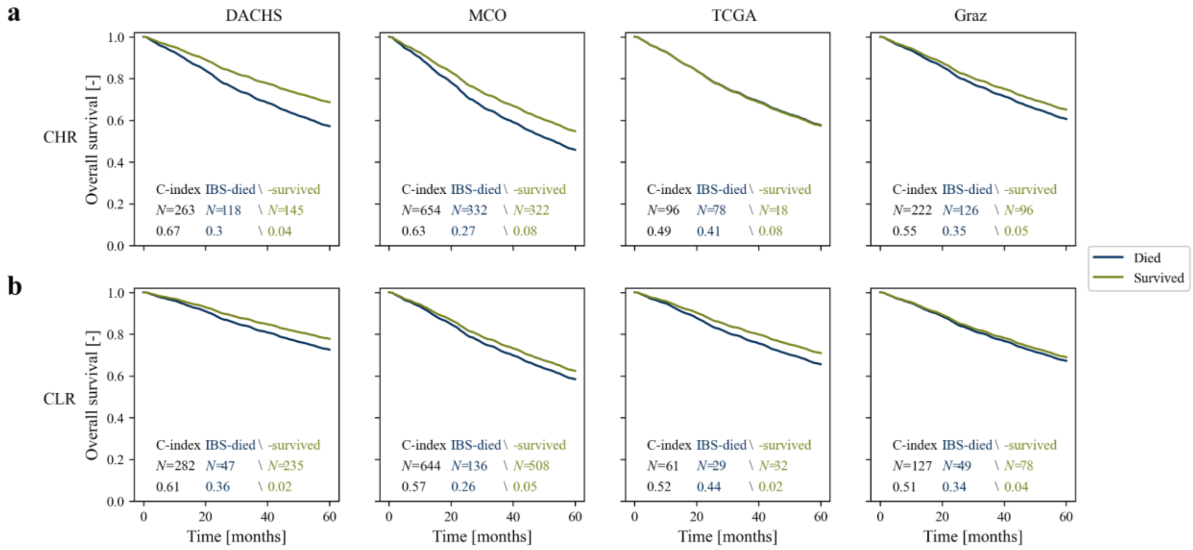

Supplementary Figure 10: Mean predicted survival curves of the DINO-tcga model (without recalibration). **a,** Mean predicted survival curves of the CHR subcohorts. **b,** Mean predicted survival curves of the CLR subcohorts. Note that all curves and metrics reported in the subfigures were calculated without censored cases. CHR=clinical high risk; CLR=clinical low risk; IBS=integrated Brier score.

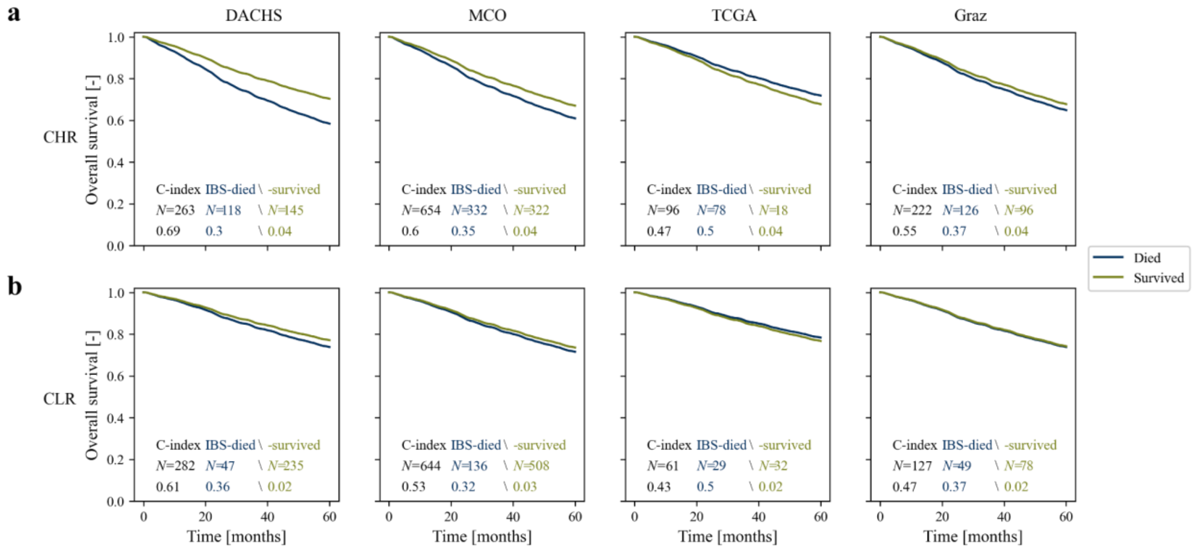

Supplementary Figure 11: Mean predicted survival curves of the R26-ViT model (without recalibration). **a,** Mean predicted survival curves of the CHR subcohorts. **b,** Mean predicted survival curves of the CLR subcohorts. Note that all curves and metrics reported in the subfigures were calculated without censored cases. CHR=clinical high risk; CLR=clinical low risk; IBS=integrated Brier score.

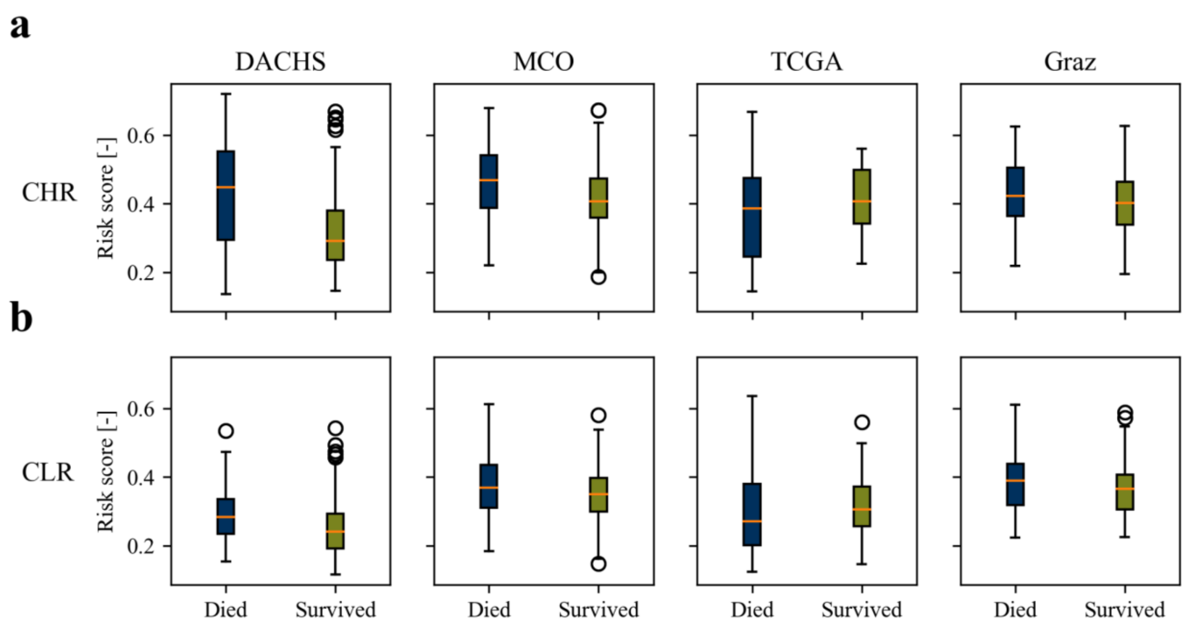

Supplementary Figure 12: Risk scores of the binary approach for the patients that died or survived the first five years. a, Risk scores within the CHR subcohorts. **b,** Risk scores within the CLR subcohorts. The binary approach uses tumor tissue only as input and is an ensemble risk score of all eight models with different feature extractors that were trained to predict a single risk score. Note that, similar to Figure 4, only the risk scores of the uncensored cases could be considered in the subfigures (for sample sizes see Figure 4). Box plot elements: the box extends from the first quartile to the third quartile of the data, with a line at the median. The whiskers extend from the box by 1.5x the inter-quartile range. Flier points are those past the end of the whiskers.
CHR=clinical high risk; CLR=clinical low risk.

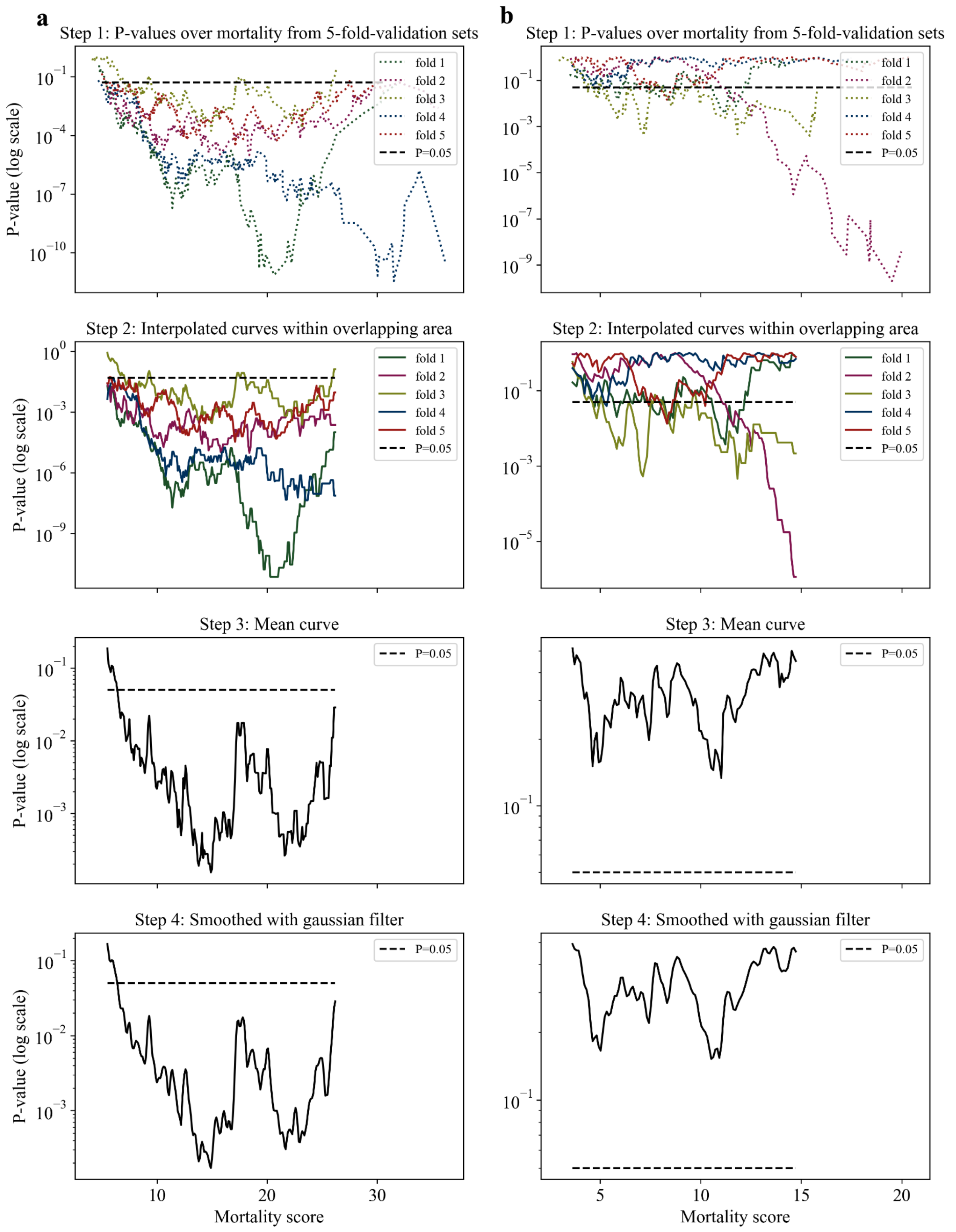

Supplementary Figure 13: Cutoff determination for the clinical risk groups on five-fold cross-validation sets of ensemble of the survival curve approach. **a,** Cutoff determination for the clinical high risk (CHR) subcohort. **b,** Cutoff determination for the clinical low risk (CLR) subcohort.

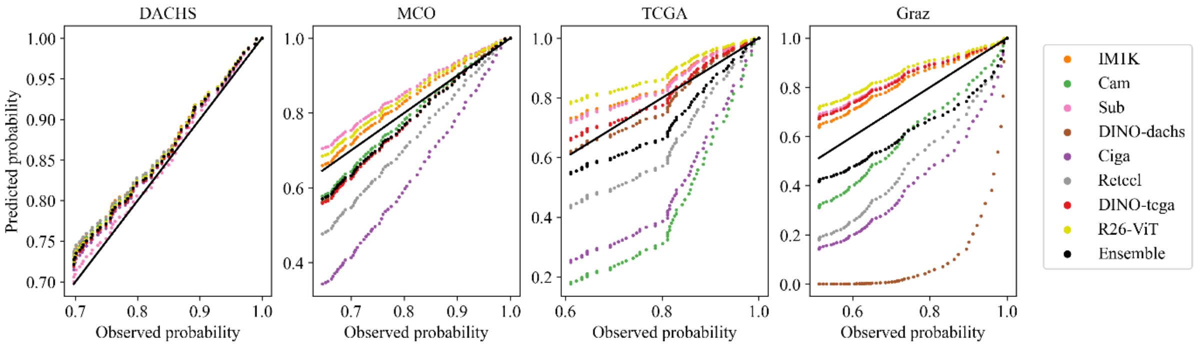

Supplementary Figure 14: Calibration curves of the all investigated image models of the survival curve approach on the test sets. Solid line represents perfect calibration.

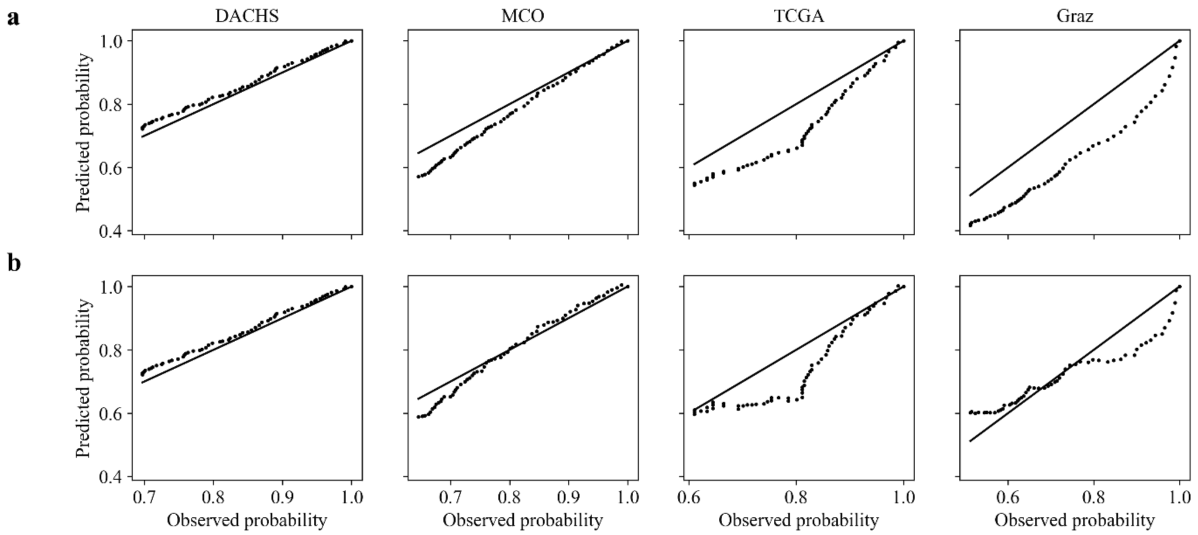

Supplementary Figure 15: Effect of recalibration on the ensemble of the survival curve approach. a, Calibration curves of the ensemble without recalibration. **b,** Calibration curves of the ensemble with recalibration. Solid line represents perfect calibration.

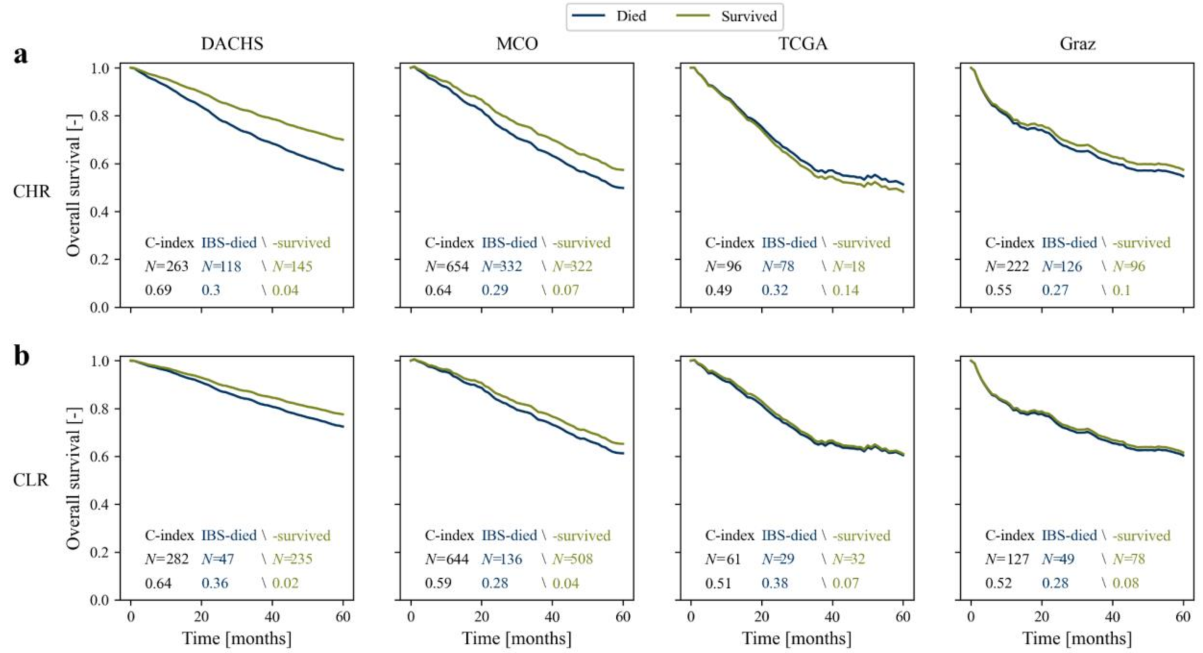

Supplementary Figure 16: Mean predicted survival curves of the ensemble of the survival curve approach with recalibration. a, Mean predicted survival curves of the CHR subcohorts. **b,** Mean predicted survival curves of the CLR subcohorts. Note that all curves and metrics reported in the subfigures were calculated without censored cases. CHR=clinical high risk; CLR=clinical low risk; IBS=integrated Brier score.

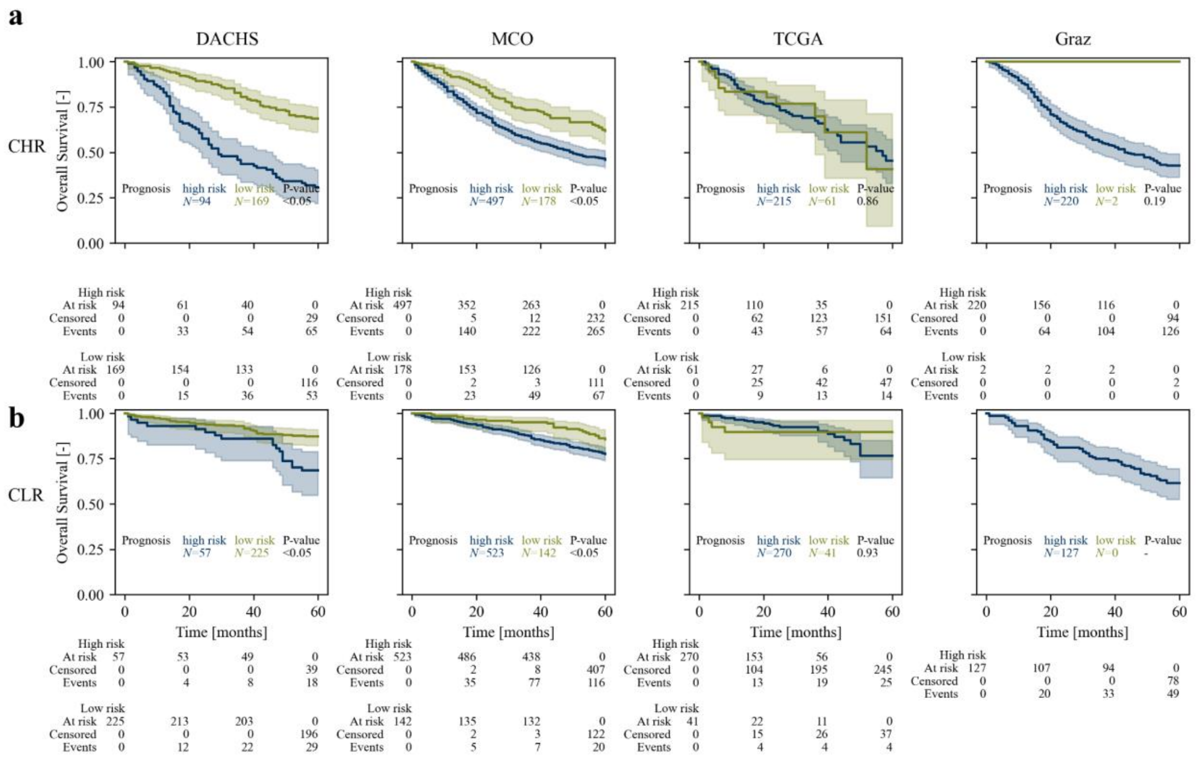

Supplementary Figure 17: Kaplan-Meier curves of "nested" refined risk groups as defined by the ensemble of the survival curve approach within clinical risk groups without recalibration on test sets. a, Nested risk groups in the CHR subcohorts. **b,** Nested risk groups in the CLR subcohorts. Mortality score cutoffs were determined according to the procedure described in Supplementary Figure 13 ($M_{Ensemble\ Cut_{CHR}} = 14.9,\ M_{Ensemble\ Cut_{CLR}} = 10.5$). CHR=clinical high risk; CLR=clinical low risk.

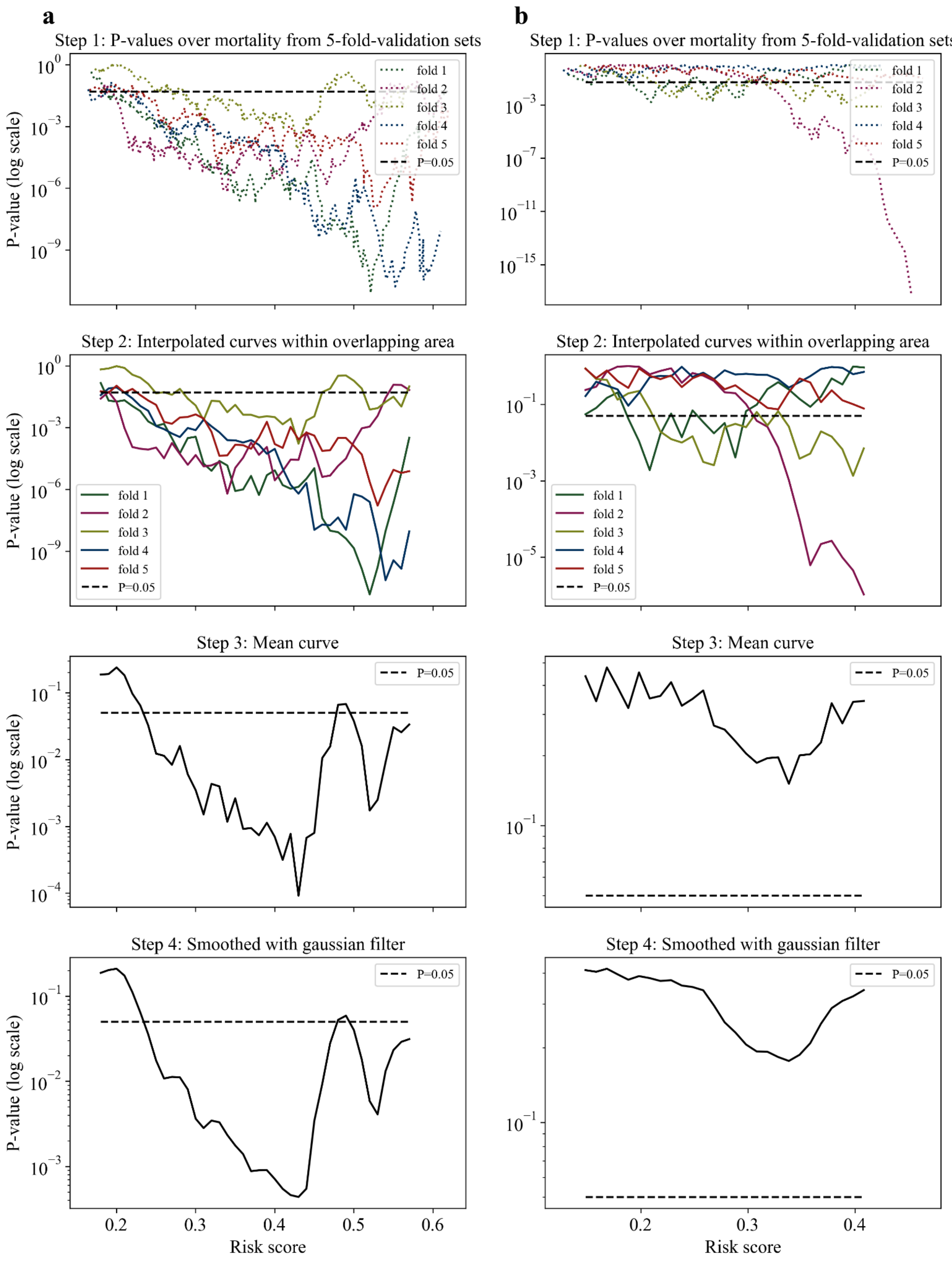

Supplementary Figure 18: Cutoff determination for the clinical risk groups on five-fold cross-validation sets of ensemble of the binary approach. **a,** Cutoff determination for the clinical high risk subcohort. **b,** Cutoff determination for the clinical low risk subcohort.

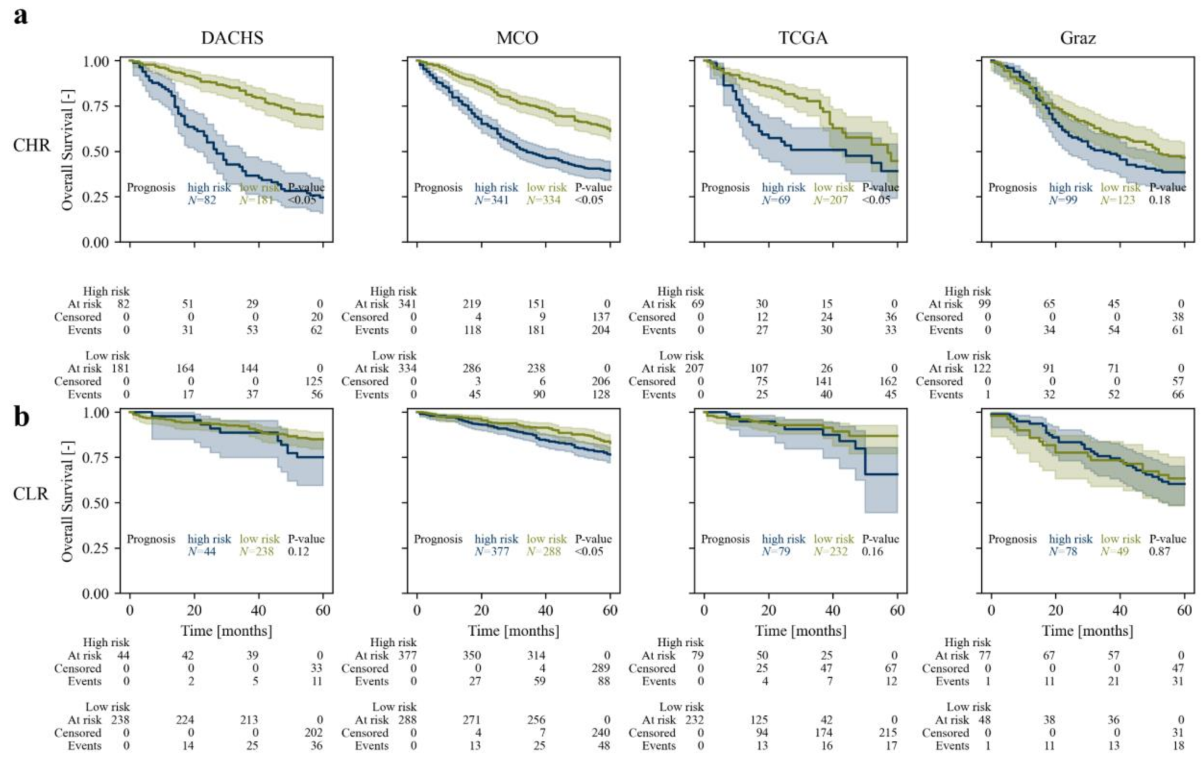

Supplementary Figure 19: Kaplan-Meier curves of “nested” refined risk groups as defined by the ensemble of the binary approach within clinical risk groups on test sets. a, Nested risk groups in the CHR subcohorts. **b,** Nested risk groups in the CLR subcohorts. Ensemble risk score cutoffs were determined according to the procedure similar as described in Supplementary Figure 18 ($Score_{Benchmark\ Cut_{CHR}} = 0.43$, $Score_{Benchmark\ Cut_{CLR}} = 0.338$). CHR=clinical high risk; CLR=clinical low risk.

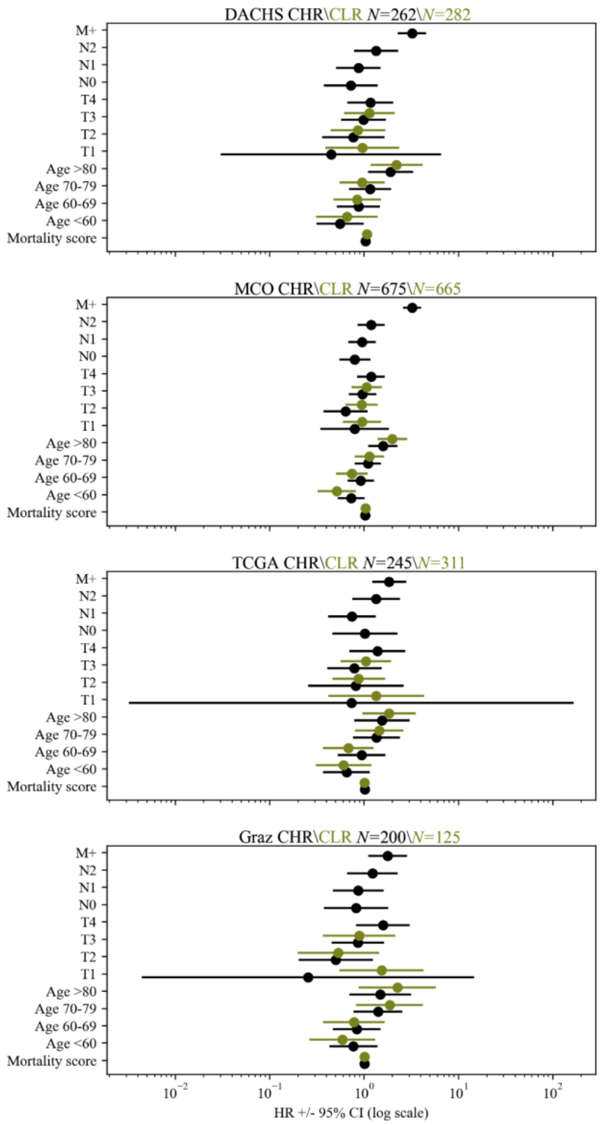

DACHS CHR\CLR N=262\N=282

	HR	P-value	HR	P-value
M+	3.23 (2.28-4.58)	<0.05	-	-
N2	1.34 (0.78-2.31)	0.282	-	-
N1	0.87 (0.51-1.5)	0.621	-	-
N0	0.72 (0.37-1.4)	0.337	-	-
T4	1.17 (0.67-2.05)	0.58	-	-
T3	0.99 (0.57-1.71)	0.972	1.15 (0.62-2.12)	0.664
T2	0.77 (0.36-1.65)	0.5	0.86 (0.44-1.68)	0.662
T1	0.45 (0.03-6.61)	0.559	0.96 (0.39-2.37)	0.929
Age >80	1.92 (1.1-3.34)	<0.05	2.21 (1.17-4.18)	<0.05
Age 70-79	1.17 (0.7-1.95)	0.56	0.96 (0.55-1.66)	0.874
Age 60-69	0.87 (0.52-1.48)	0.616	0.85 (0.48-1.52)	0.58
Age <60	0.56 (0.31-1.0)	<0.05	0.66 (0.31-1.4)	0.28
Mortality score	1.04 (1.02-1.06)	<0.05	1.07 (1.01-1.13)	<0.05

MCO CHR\CLR N=675\N=665

	HR	P-value	HR	P-value
M+	3.25 (2.6-4.06)	<0.05	-	-
N2	1.2 (0.86-1.67)	0.292	-	-
N1	0.96 (0.69-1.33)	0.788	-	-
N0	0.8 (0.55-1.17)	0.25	-	-
T4	1.19 (0.85-1.67)	0.315	-	-
T3	0.97 (0.69-1.35)	0.841	1.07 (0.74-1.55)	0.72
T2	0.64 (0.37-1.09)	0.1	0.95 (0.64-1.41)	0.792
T1	0.8 (0.34-1.84)	0.596	0.95 (0.6-1.52)	0.837
Age >80	1.59 (1.12-2.27)	<0.05	2.0 (1.39-2.88)	<0.05
Age 70-79	1.1 (0.8-1.52)	0.56	1.14 (0.8-1.63)	0.475
Age 60-69	0.93 (0.67-1.28)	0.638	0.75 (0.51-1.09)	0.134
Age <60	0.73 (0.53-1.02)	0.062	0.51 (0.32-0.82)	<0.05
Mortality score	1.03 (1.02-1.05)	<0.05	1.04 (1.01-1.07)	<0.05

TCGA CHR\CLR N=245\N=311

	HR	P-value	HR	P-value
M+	1.85 (1.23-2.8)	<0.05	-	-
N2	1.34 (0.75-2.4)	0.317	-	-
N1	0.75 (0.42-1.33)	0.324	-	-
N0	1.02 (0.46-2.27)	0.952	-	-
T4	1.39 (0.7-2.74)	0.35	-	-
T3	0.79 (0.41-1.54)	0.496	1.05 (0.57-1.95)	0.869
T2	0.82 (0.25-2.63)	0.738	0.88 (0.46-1.67)	0.696
T1	0.74 (0.0-167.14)	0.912	1.35 (0.42-4.34)	0.617
Age >80	1.55 (0.79-3.07)	0.203	1.85 (0.97-3.54)	0.064
Age 70-79	1.35 (0.77-2.4)	0.297	1.45 (0.8-2.61)	0.216
Age 60-69	0.95 (0.53-1.69)	0.851	0.68 (0.37-1.27)	0.227
Age <60	0.65 (0.37-1.15)	0.143	0.61 (0.31-1.2)	0.151
Mortality score	1.02 (1.0-1.04)	0.061	1.01 (0.98-1.05)	0.402

Graz CHR\CLR N=200\N=125

	HR	P-value	HR	P-value
M+	1.78 (1.11-2.87)	<0.05	-	-
N2	1.23 (0.66-2.28)	0.51	-	-
N1	0.87 (0.47-1.62)	0.661	-	-
N0	0.82 (0.38-1.81)	0.631	-	-
T4	1.59 (0.83-3.06)	0.165	-	-
T3	0.86 (0.45-1.63)	0.642	0.89 (0.37-2.15)	0.793
T2	0.5 (0.2-1.24)	0.137	0.54 (0.2-1.45)	0.218
T1	0.26 (0.0-14.8)	0.511	1.54 (0.55-4.31)	0.412
Age >80	1.49 (0.7-3.16)	0.297	2.26 (0.88-5.82)	0.091
Age 70-79	1.41 (0.78-2.55)	0.256	1.87 (0.83-4.23)	0.131
Age 60-69	0.84 (0.47-1.51)	0.563	0.78 (0.37-1.67)	0.525
Age <60	0.77 (0.43-1.39)	0.389	0.59 (0.26-1.32)	0.199
Mortality score	1.02 (0.99-1.04)	0.167	1.01 (0.97-1.05)	0.528

Supplementary Figure 20: Multivariable Cox regression analyses including mortality score and known risk factors for clinical risk groups. Multivariable Cox proportional hazard models were fitted on mortality score of ensemble image model with recalibration on test sets (continuous variable, unnormalized) and known risk factors (categorical) for both clinical risk groups separately. Cases with missing risk factors were excluded. CHR=clinical high risk; CLR=clinical low risk; HR=hazard ratio.

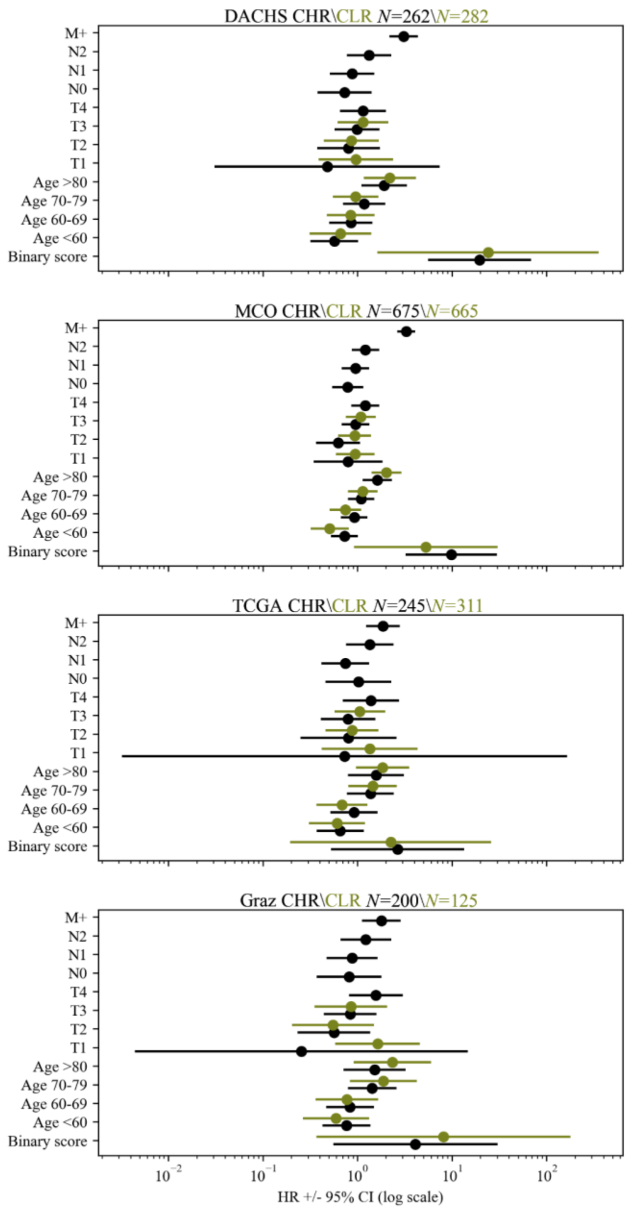

DACHS

	HR	P-value	HR	P-value
M+	3.09 (2.18-4.37)	<0.05	-	-
N2	1.32 (0.77-2.27)	0.307	-	-
N1	0.88 (0.51-1.51)	0.646	-	-
N0	0.73 (0.38-1.42)	0.353	-	-
T4	1.15 (0.65-2.01)	0.634	-	-
T3	0.99 (0.57-1.72)	0.974	1.14 (0.62-2.12)	0.674
T2	0.8 (0.38-1.73)	0.578	0.87 (0.44-1.69)	0.672
T1	0.48 (0.03-7.42)	0.599	0.96 (0.39-2.38)	0.932
Age >80	1.92 (1.1-3.34)	<0.05	2.2 (1.16-4.17)	<0.05
Age 70-79	1.17 (0.7-1.96)	0.541	0.96 (0.55-1.66)	0.877
Age 60-69	0.85 (0.5-1.44)	0.551	0.85 (0.48-1.52)	0.579
Age <60	0.57 (0.32-1.02)	0.057	0.66 (0.31-1.4)	0.281
Binary score	19.58 (5.58-68.64)	<0.05	24.06 (1.62-356.87)	<0.05

MCO

	HR	P-value	HR	P-value
M+	3.28 (2.63-4.1)	<0.05	-	-
N2	1.21 (0.87-1.69)	0.258	-	-
N1	0.95 (0.68-1.32)	0.761	-	-
N0	0.79 (0.54-1.16)	0.226	-	-
T4	1.21 (0.86-1.7)	0.269	-	-
T3	0.95 (0.68-1.34)	0.785	1.09 (0.75-1.57)	0.661
T2	0.63 (0.37-1.07)	0.086	0.93 (0.63-1.39)	0.733
T1	0.8 (0.34-1.85)	0.596	0.95 (0.59-1.51)	0.821
Age >80	1.62 (1.14-2.31)	<0.05	2.03 (1.41-2.91)	<0.05
Age 70-79	1.09 (0.79-1.51)	0.583	1.14 (0.8-1.63)	0.473
Age 60-69	0.92 (0.67-1.27)	0.632	0.74 (0.51-1.09)	0.129
Age <60	0.73 (0.53-1.01)	0.059	0.51 (0.32-0.81)	<0.05
Binary score	9.8 (3.23-29.69)	<0.05	5.28 (0.92-30.45)	0.063

TCGA

	HR	P-value	HR	P-value
M+	1.86 (1.23-2.81)	<0.05	-	-
N2	1.35 (0.76-2.41)	0.308	-	-
N1	0.74 (0.42-1.33)	0.317	-	-
N0	1.02 (0.46-2.27)	0.957	-	-
T4	1.39 (0.7-2.75)	0.344	-	-
T3	0.8 (0.41-1.54)	0.498	1.06 (0.57-1.96)	0.857
T2	0.8 (0.25-2.58)	0.715	0.88 (0.46-1.67)	0.685
T1	0.73 (0.0-164.93)	0.91	1.35 (0.42-4.33)	0.618
Age >80	1.58 (0.8-3.11)	0.19	1.85 (0.96-3.54)	0.064
Age 70-79	1.37 (0.78-2.43)	0.278	1.45 (0.8-2.61)	0.218
Age 60-69	0.92 (0.52-1.64)	0.783	0.68 (0.37-1.27)	0.231
Age <60	0.66 (0.37-1.16)	0.149	0.61 (0.31-1.2)	0.15
Binary score	2.65 (0.52-13.38)	0.238	2.25 (0.19-25.89)	0.516

Graz

	HR	P-value	HR	P-value
M+	1.79 (1.11-2.87)	<0.05	-	-
N2	1.22 (0.66-2.27)	0.525	-	-
N1	0.88 (0.47-1.63)	0.684	-	-
N0	0.82 (0.37-1.79)	0.614	-	-
T4	1.57 (0.81-3.02)	0.178	-	-
T3	0.84 (0.44-1.59)	0.585	0.85 (0.35-2.07)	0.726
T2	0.56 (0.23-1.36)	0.204	0.55 (0.2-1.49)	0.239
T1	0.26 (0.0-14.72)	0.51	1.63 (0.58-4.58)	0.351
Age >80	1.52 (0.71-3.22)	0.279	2.33 (0.91-5.98)	0.079
Age 70-79	1.43 (0.79-2.59)	0.234	1.87 (0.83-4.24)	0.131
Age 60-69	0.83 (0.47-1.49)	0.539	0.77 (0.36-1.65)	0.503
Age <60	0.77 (0.43-1.38)	0.375	0.59 (0.26-1.32)	0.2
Binary score	4.1 (0.56-30.27)	0.166	8.12 (0.37-178.38)	0.184

Supplementary Figure 21: Multivariable Cox regression analyses including the score of the binary approach and known risk factors for clinical risk groups. Multivariable Cox proportional hazard models were fitted on the score of the ensemble image model of the binary approach on test sets (continuous variable, unnormalized) and known risk factors (categorical) for both clinical risk groups separately. Cases with missing risk factors were excluded. CHR=clinical high risk; CLR=clinical low risk; HR=hazard ratio.
