# Supplementary material for: Colorectal cancer risk stratification on histological slides based on survival curves predicted by deep learning
Source: NPJ Precis Oncol. 2023 Sep 26;7:98. doi: 10.1038/s41698-023-00451-3 (PMC10522577; doi:10.1038/s41698-023-00451-3)
Supplement: Supplementary file 3 — Reporting summary [file 41698_2023_451_MOESM3_ESM.pdf]

Corresponding author(s): Titus J. Brinker
NPJPRECISIONONCOLOGY-03430R

Last updated by author(s): Sep 1, 2023

Reporting Summary

Nature Portfolio wishes to improve the reproducibility of the work that we publish. This form provides structure for consistency and transparency in reporting. For further information on Nature Portfolio policies, see our Editorial Policies and the Editorial Policy Checklist.

Statistics

For all statistical analyses, confirm that the following items are present in the figure legend, table legend, main text, or Methods section.

n/a	Confirmed	
☐	☒	The exact sample size (*n*) for each experimental group/condition, given as a discrete number and unit of measurement
☐	☒	A statement on whether measurements were taken from distinct samples or whether the same sample was measured repeatedly
☐	☒	The statistical test(s) used AND whether they are one- or two-sided<br>*Only common tests should be described solely by name; describe more complex techniques in the Methods section.*
☐	☒	A description of all covariates tested
☒	☐	A description of any assumptions or corrections, such as tests of normality and adjustment for multiple comparisons
☐	☒	A full description of the statistical parameters including central tendency (e.g. means) or other basic estimates (e.g. regression coefficient) AND variation (e.g. standard deviation) or associated estimates of uncertainty (e.g. confidence intervals)
☐	☒	For null hypothesis testing, the test statistic (e.g. *F*, *t*, *r*) with confidence intervals, effect sizes, degrees of freedom and *P* value noted<br>*Give P values as exact values whenever suitable.*
☒	☐	For Bayesian analysis, information on the choice of priors and Markov chain Monte Carlo settings
☒	☐	For hierarchical and complex designs, identification of the appropriate level for tests and full reporting of outcomes
☒	☐	Estimates of effect sizes (e.g. Cohen's *d*, Pearson's *r*), indicating how they were calculated

Our web collection on statistics for biologists contains articles on many of the points above.

Software and code

Policy information about availability of computer code

Data collection	There was no new data collected in the study.
Data analysis	All code was written in Python (3.10.6). The image analysis pipeline used PyTorch (1.13.0+cu117), Pytorch Lightning (1.7.7), NumPy (1.23.3), Pandas (1.5.0) and Scipy (1.9.1). The negative log likelihood loss and functions for the piecewise constant hazard method and to transform the models' output into a survival curve as well as the time-dependent C-index were taken from pycox (0.2.3). The integrated Brier score and (conventional) Brier score were calculated using sksurv (0.18.0). For the C-index calculation of the binary approach, the Kaplan Meier fitter, Cox proportional hazard fitter and the logrank tests we used the respective functions of lifelines (0.27.2). For visualizations we used matplotlib (3.7.2). Image augmentations (training of the subtyper) were done with torchvision (0.14.0+cu117). Accuracy of the subtyper was calculated with torchmetrics (0.9.3). Pre-trained models were taken from timm (0.6.11), if not stated otherwise.

For manuscripts utilizing custom algorithms or software that are central to the research but not yet described in published literature, software must be made available to editors and reviewers. We strongly encourage code deposition in a community repository (e.g. GitHub). See the Nature Portfolio guidelines for submitting code & software for further information.

Data

Policy information about availability of data

All manuscripts must include a data availability statement. This statement should provide the following information, where applicable:

- Accession codes, unique identifiers, or web links for publicly available datasets
- A description of any restrictions on data availability
- For clinical datasets or third party data, please ensure that the statement adheres to our policy

This study used archived pseudonymized pathology slides, clinicopathologic variables and corresponding outcome data from the DACHS, MCO, TCGA and Graz cohorts. The DACHS, MCO and Graz cohorts cannot be made publicly available due to general data protection regulations and institutional guidelines. Interested researchers should contact M.H. in case of the DACHS patient cohort and K.Z. in case of the Graz cohort. For access to the MCO cohort visit https://doi.org/10.4225/53/5559205bea135. The TCGA cohort used in this study consists of the TCGA COAD and READ cohorts (both dbGaP accession: phs000178.v11.p8) and original data of both cohorts are publicly available under https://portal.gdc.cancer.gov/ and http://www.cbioportal.org/. Included case IDs of the TCGA cohort are provided in Supplementary Data 1.

Research involving human participants, their data, or biological material

Policy information about studies with human participants or human data. See also policy information about sex, gender (identity/presentation), and sexual orientation and race, ethnicity and racism.

Reporting on sex and gender	Sex was a variable examined in this study and data from both sexes are reported in the study. Information on gender was not available. Sample size was too small to perform meaningful sex-based analysis separately.
Reporting on race, ethnicity, or other socially relevant groupings	Information on race, ethnicity or other socially relevant groupings was not examined in this study.
Population characteristics	The population studied is patients with stage I-IV colorectal cancer with available histological tissue samples of primary colorectal cancer.
Recruitment	The DACHS patient cohort was recruited within a population-based case-control study from southwestern Germany between 2003 and 2014. The MCO cohort is a collection of imaging, specimen, clinical and genetic data from Australian individuals who underwent curative resection for CRC from 1994 to 2010. The Cancer Genome Atlas (TCGA) cohort (TCGA-READ and -COAD) is an international multicentre cohort mainly from the United States with cancers diagnosed between 1998 and 2013. The Graz cohort consists of pathology slides from the Institute of Pathology and the BioBank at the Medical University in Graz of CRC resection cases between 1985 and 2016, while 80% of the resections were diagnosed after 1997.
Ethics oversight	Data and digitized slides were provided in accordance with the approval of the ethics committees of the Medical Faculty of the University of Heidelberg and the Medical Chambers of Baden-Württemberg and Rhineland-Palatinate for DACHS and in accordance with the approval of the Secure Research Environment for Digital Health (SREDH) Consortium for MCO. TCGA is open source. For Graz, Institutional Review Board approval for this retrospective study using de-identified slides was obtained from the Medical University of Graz (Protocol now. 30-184 ex 17/18).

Note that full information on the approval of the study protocol must also be provided in the manuscript.

Field-specific reporting

Please select the one below that is the best fit for your research. If you are not sure, read the appropriate sections before making your selection.

☒ Life sciences ☐ Behavioural & social sciences ☐ Ecological, evolutionary & environmental sciences

For a reference copy of the document with all sections, see nature.com/documents/nr-reporting-summary-flat.pdf

Life sciences study design

All studies must disclose on these points even when the disclosure is negative.

Sample size	Sample size is based on all patients in the DACHS, MCO, TCGA and Graz cohort with stage I-IV colorectal cancer, survival data and at least one representative pre-treatment diagnostic slide available. No sample-size calculation was performed. We used all available cases to enable optimal training and model building. Sample sizes for the different data sets are reported in the manuscript.
Data exclusions	Cases were eligible if at least one representative pre-treatment diagnostic slide and information on an event or censoring time was available. No (other) image quality checks to exclude cases were undertaken. The resulting patient numbers are also reported in the manuscript.
Replication	We split the DACHS cohort which served as the training cohort of all our models into five folds and performed five-fold cross-validation to develop the models. All models were therefore trained five times with different training/validation set distributions but identical hyperparamters.

Randomization	The DACHS cohort was randomly split into five folds for five-fold cross-validation prior to model training. There was no randomized intervention in the study.
Blinding	For the Graz cohort, the DKFZ group that did the testing was blinded to the patient outcome, as the DKFZ-trained algorithm was executed on the Graz test slides by the collaborators in Graz and the DKFZ group had no access to the Graz data. For the other cohorts, the DKFZ group had direct access to the data. The algorithm was trained on the training and validation set, without further adjustments after testing on the respective test sets in all cases.

Reporting for specific materials, systems and methods

We require information from authors about some types of materials, experimental systems and methods used in many studies. Here, indicate whether each material, system or method listed is relevant to your study. If you are not sure if a list item applies to your research, read the appropriate section before selecting a response.

Materials & experimental systems

n/a	Involved in the study
☒	☐ Antibodies
☒	☐ Eukaryotic cell lines
☒	☐ Palaeontology and archaeology
☒	☐ Animals and other organisms
☐	☒ Clinical data
☒	☐ Dual use research of concern
☒	☐ Plants

Methods

n/a	Involved in the study
☒	☐ ChIP-seq
☒	☐ Flow cytometry
☒	☐ MRI-based neuroimaging

Clinical data

Policy information about clinical studies

All manuscripts should comply with the ICMJE guidelines for publication of clinical research and a completed CONSORT checklist must be included with all submissions.

Clinical trial registration	n.a. This was a basic research study with archived data only.
Study protocol	n.a. This was a basic research study with archived data only.
Data collection	There was no new data collected for this study.
Outcomes	Our pre-defined endpoint was a significant difference between survival risk groups that patients were allocated to using the prognostic models trained in the study. To train the models overall survival was used as patient outcome, because that prognostic information was uniformly and reliably available across cohorts.
